# Regulation of intestinal senescence during cholestatic liver disease modulates barrier function and liver disease progression

**DOI:** 10.1016/j.jhepr.2024.101159

**Published:** 2024-06-29

**Authors:** Mar Moreno-Gonzalez, Katherine Hampton, Paula Ruiz, Gemma Beasy, Falk SP. Nagies, Aimee Parker, James Lazenby, Caitlin Bone, Ane Alava-Arteaga, Meha Patel, Charlotte Hellmich, Pablo Luri-Martin, Ece Silan, Mark Philo, David Baker, Simon M. Rushbrook, Falk Hildebrand, Stuart A. Rushworth, Naiara Beraza

**Affiliations:** 1 Gut Microbes and Health Institute Strategic Programme, Quadram Institute Bioscience, Norwich Research Park, Norwich, UK; 2 Food, Microbiome and Health Institute Strategic Programme, Quadram Institute Bioscience, Norwich Research Park, Norwich, UK; 3 Centre for Metabolic Health, Faculty of Medicine, University of East Anglia, Norwich Research Park, Norwich, UK; 4 Earlham Institute, Norwich Research Park, Norwich, UK; 5 Science Operations, Quadram Institute Bioscience, Norwich Research Park, Norwich, UK; 6 Department of Haematology, Norfolk and Norwich University Hospital, Norwich, UK; 7 Department of Gastroenterology, Norfolk and Norwich University Hospital, Norwich, UK; 8 Food Innovation and Health Institute Strategic Programme, Quadram Institute Bioscience, Norwich Research Park, Norwich, UK

**Keywords:** Senescence, intestine, cholestasis, liver, senolytics

## Abstract

**Background & Aims:**

Senescence has been reported to have differential functions in cholangiocytes and hepatic stellate cells (HSCs) during human and murine cholestatic disease, being detrimental in biliary cells and anti-fibrotic in HSCs. Cholestatic liver disease is associated with loss of intestinal barrier function and changes in the microbiome, the mechanistic cause of which is undetermined.

**Methods:**

Intestinal samples were analysed from controls and patients with primary sclerosing cholangitis, as well as wild-type (WT) and p16-3MR transgenic mice. Cholestatic liver disease was induced by bile duct ligation (BDL) and DDC diet feeding. Fexaramine was used as an intestinal-restricted FXR agonist and antibiotics were given to eliminate the intestinal microbiome. Senescent cells were eliminated in p16-3MR mice with ganciclovir and in WT mice with the senolytic drug ABT-263. *In vitro* studies were done in intestinal CaCo-2 cells and organoids were generated from intestinal crypts isolated from mice.

**Results:**

Herein, we show increased senescence in intestinal epithelial cells (IECs) in patients with primary sclerosing cholangitis and in mice after BDL and DDC diet feeding. Intestinal senescence was increased in response to reduced exposure to bile acids and increased presence of lipopolysaccharide *in vitro* and *in vivo* during cholestatic liver disease. Senescence of IECs was associated with lower proliferation but increased intestinal stem cell activation, as supported by increased organoid growth from intestinal stem cells. Elimination of senescent cells with genetic and pharmacological approaches exacerbated liver injury and fibrosis during cholestatic liver disease, which was associated with increased IEC apoptosis and permeability.

**Conclusions:**

Senescence occurs in IECs during cholestatic disease and the elimination of senescent cells has a detrimental impact on the gut-liver axis. Our results point to cell-specific rather than systemic targeting of senescence as a therapeutic approach to treat cholestatic liver disease.

**Impact and implications:**

Cholestatic liver disease associates with the dysregulation of intestinal barrier function, while the mechanisms mediating the disruption of the gut-liver axis remain largely undefined. Here, we demonstrate that senescence, a cellular response to stress, is activated in intestinal cells during cholestatic liver disease in humans and mice. Mechanistically, we demonstrate that the reduction of bile acids and the increased presence of bacterial products mediate the activation of intestinal senescence during cholestatic liver disease. Importantly, the elimination of these senescent cells promotes further damage to the intestine that aggravates liver disease, with increased tissue damage and fibrosis. Our results provide evidence that therapeutic strategies to treat cholestatic liver disease by eliminating senescent cells may have unwanted effects in the intestine and support the need to develop cell/organ-specific approaches.

## Highlights

• Senescence is induced in the intestine during human and murine cholestatic disease.
• Increased presence of bacteria mediate senescence in the intestine during cholestatic liver disease.
• Senescence regulates the intestine regenerative response during cholestatic disease.
• Elimination of senescence aggravates liver injury and fibrosis by disrupting barrier integrity during cholestatic liver disease.

## Graphical abstract

**Figure:**
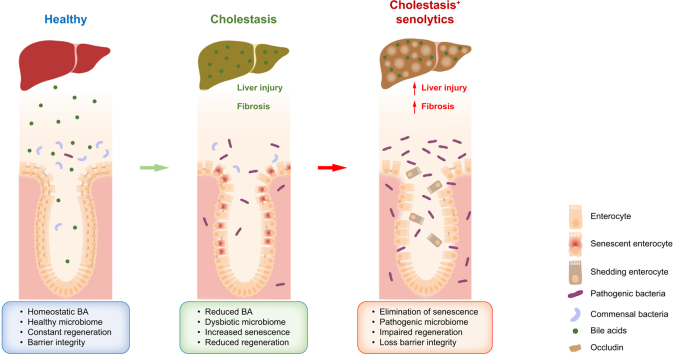

## Introduction

Senescence is a cellular response to stress and damage that prevents growth of transformed cells^1^ and improves tissue renewal and wound repair.^2^ Nonetheless, senescence also has detrimental effects when persistent or unresolved and is implicated in the development of pathologies including liver disease.[3], [4], [5], [6], [7], [8] In particular, biliary cell senescence has been described in livers from patients with primary biliary cholangitis^3^^,^^4^^,^^7^ and primary sclerosing cholangitis (PSC),^3^^,^^5^^,^^6^^,^^8^ where senescence is associated with disease severity.^3^^,^^8^^,^^9^ Liver senescence has also been described in preclinical mouse models of cholestatic liver disease, including 3,5-diethoxycarbonyl-1,4-dihydrocollidine (DDC) diet^10^ and bile duct ligation (BDL),[11], [12], [13] where senescence was detrimental when found in cholangiocytes,[12], [13], [14] but had antifibrotic properties when expressed in hepatic stellate cells (HSCs).[11], [12], [13] Therapeutic attempts focused on inhibition of senescence in biliary cells/cholangiocytes have been shown to reduce fibrosis during preclinical cholestatic disease.^13^^,^[15], [16], [17], [18]

The intestinal epithelia of the small intestine consists of a monolayer of columnar cells (intestinal epithelial cells; IECs) composed of Lgr5+-intestinal stem cells (ISCs) and Paneth cells, with antimicrobial activity, residing in the crypt base whereas the villus is composed of enteroendocrine cells (<1% prevalent), goblet cells (mucus-producing cells comprising 4-16% of the epithelia), tuft cells (<1%) and absorptive enterocytes, which represent the vast majority of IECs in the villi.^19^ Colonic structure and cell composition are similar while the epithelial surface is flat and lacking Paneth cells. Enterocytes/colonocytes preserve intestinal permeability by expressing tight junction proteins (*i.e.* occludin) that impair the translocation of large molecules and bacteria across the epithelial barrier.^20^ During homeostasis the intestine is constantly regenerating, with crypt-base ISCs proliferating, differentiating, and migrating up through the villi every 2-5 days, repopulating the epithelia as IECs.^21^^,^^22^ After injury, ISC self-renewal is tightly regulated to ensure the regeneration of the intestinal epithelia.^22^

It is well established that chronic liver disease, including cholestatic disease, is associated with profound changes in intestinal microbiome composition and disruption of intestinal (barrier) function, leading to increased permeability, unresolved inflammation, and disease progression.^23^ However, the mechanisms mediating these functional changes observed in the intestine during cholestatic liver disease remain largely undefined.

Here, we describe the regulation of cellular senescence in the small and large intestine during cholestatic liver disease, with the reduction in intestinal bile acids and bacterial growth as mechanistic mediators. Using genetic and pharmacological approaches to eliminate senescent cells, we define the functional relevance of senescence as a cellular stress response in the intestinal epithelia during cholestatic liver disease.

## Materials and methods

Please see the supplementary materials and methods provided in the supplementary information.

## Results

### Senescence is activated in the intestine during human and murine cholestatic liver disease

**Fig. 1:**
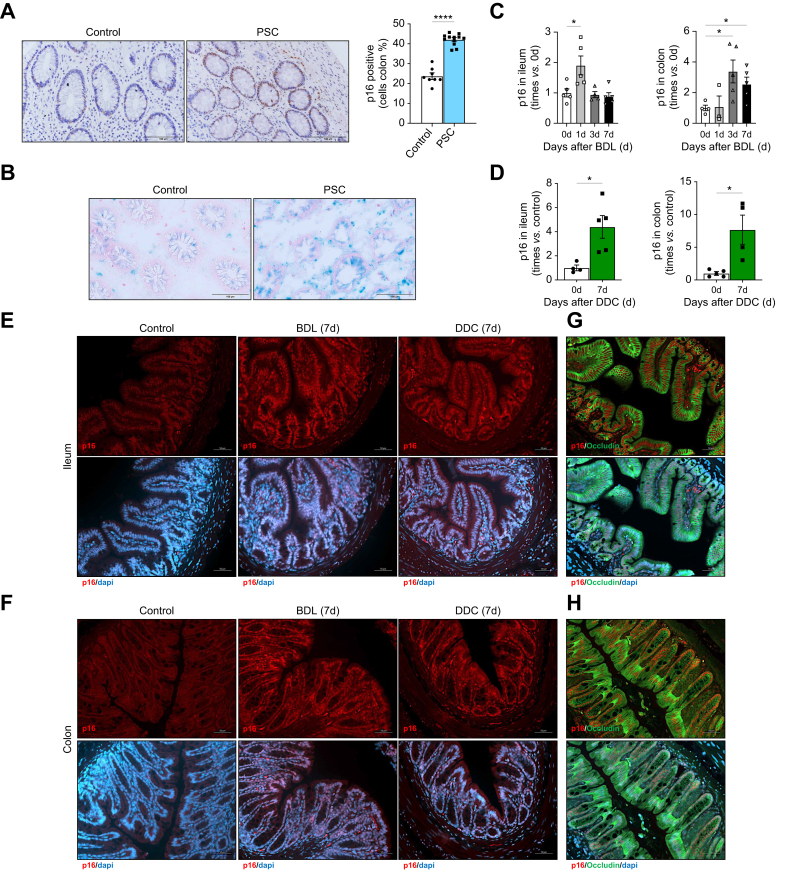
Senescence is increased in the intestine during human and murine cholestasis after BDL and DDC feeding. (A) Immunohistochemistry using a p16-antibody, quantification of positive cells (control *vs.* PSC, ∗∗∗∗*p <*0.0001) and (B) SA-β-Gal staining in colonic biopsies from patients with PSC and controls. qPCR of p16 gene expression in ileum and colon from (C) BDL (Ileum 0 h *vs.* 1 d, ∗*p* = 0.0427; Colon 0 h *vs.* 3 d, ∗*p* = 0.0287; Colon 0 h *vs*. 7 d, ∗*p =* 0.0318) and (D) DDC-fed mice (after 7 days) (ileum, ∗*p* = 0.0185; colon, ∗*p* = 0.0452). (E) p16 immunofluorescent staining in ileum and (F) colon samples from BDL and DDC-fed mice. (G, H) Immunofluorescence co-staining with p16 (red), occludin (green) and dapi (blue) in ileum and colon after BDL. Analyses were done from n = 8 control and n = 12 PSC colon biopsies and n = 5-6 mice. Representative microscopical images are shown at 40x (A, B), 20x (E-H) magnification. Values are mean ± SEM. Statistical differences were determined using Welch’s t-test. BDL, bile duct ligation; DDC, 3,5-diethoxycarbonyl-1,4-dihydrocollidine; PSC, primary sclerosing cholangitis.

Here, we show increased numbers of p16-positive cells (Fig. 1A) and increased SA-β-galactosidase staining, the gold standard for detection of senescent cells (Fig. 1B), in colonic samples from patients with PSC compared with control individuals. The clinical characteristics of the disease cohort are shown in Table S1.

Increased intestinal senescence was confirmed in two preclinical mouse models of cholestatic liver disease: BDL and DDC feeding. We found increased p16 gene expression (Fig. 1C,D), p16 immunofluorescence staining (Fig. 1E,F) and SA-β-Galactosidase staining (Fig. S1A) in the small intestine (ileum) and colon of cholestatic mice. Immunofluorescence of p16 and occludin, a tight junction protein expressed in IECs,^20^ confirmed these as the main senescent cells in the intestine during cholestatic liver disease (Fig. 1G,H and Fig. S1B).

### Intestinal senescence is associated with lower regenerative capacity despite increased stemness during cholestatic liver disease

**Fig. 2:**
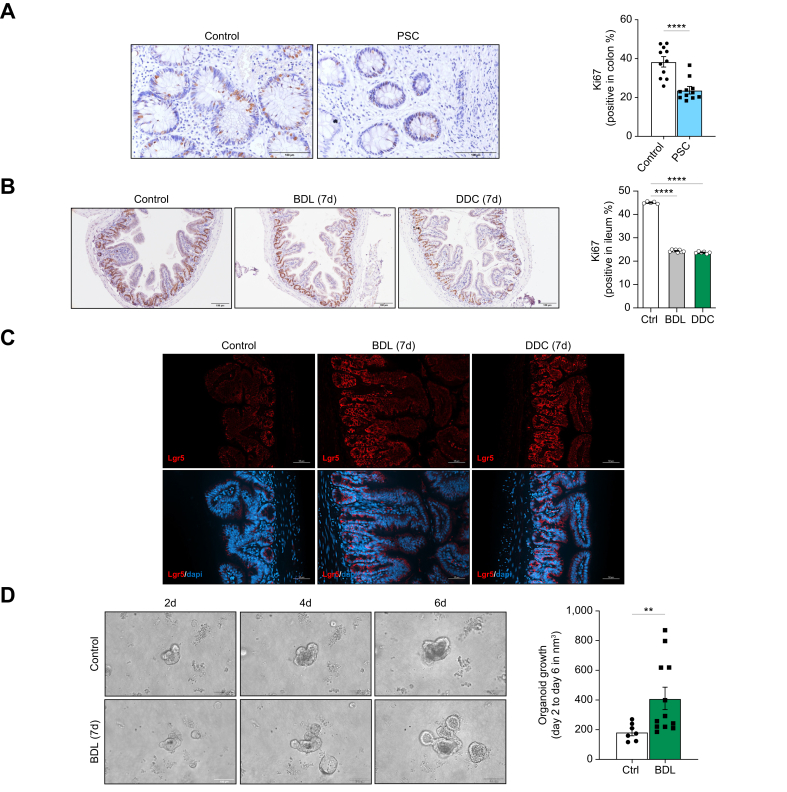
Reduced proliferation in the intestine from patients with PSC and cholestatic mice with increased ISC activation. Ki67 immunohistochemistry and quantification of Ki67-positive IECs in colon biopsies from (A) n = 11 patients with PSC and n = 9 controls (control *vs.* PSC, ∗∗∗∗*p* <0.0001; Welch’s t-test) and (B) BDL and DDC-fed mice (control *vs.* BDL *vs.* DDC, ∗∗∗∗*p* <0.0001; Brown-Forsythe and Welch one-way ANOVA). (C) Lg5+ immunofluorescence in ileums from BDL and DDC-fed mice. (D) Organoids grown from ISCs isolated from ileum crypts from control (n = 2) and BDL mice (n = 3) at days 2, 4 and 6 in culture and further quantification (control *vs.* BDL, ∗∗*p* = 0.0099; Welch’s t-test). Analyses were done from n = 5-6 mice. Representative microscopical images are shown at 40x (A), 10x (B) and 20x (C) magnification. Values are mean ± SEM. BDL, bile duct ligation; DDC, 3,5-diethoxycarbonyl-1,4-dihydrocollidine; IECs, intestinal epithelial cells; ISCs, intestinal stem cells; PSC, primary sclerosing cholangitis.

In line with the antiproliferative characteristic of senescent cells, we found significantly fewer Ki67-positive cells in the colon from patients with PSC (Fig. 2A) and the small intestine (Fig. 2B and Fig. S2A,B) and colon (Fig. S2C and D) of mice after BDL and DDC feeding.

Intestinal cell proliferation and regeneration is tightly regulated by the activation of self-renewing Lgr5^+^ ISCs at the crypt base^19^ (Fig. S3A). We found increased expression of Lgr5 in the small (Fig. 2C) and large intestine (Fig. S3B) in BDL and DDC-fed mice. Further qPCR analysis on IECs isolated from the ileum from BDL and DDC mice confirmed the increased expression of Lgr5 in cells outside the basal crypt compartment (Fig. S3C). These results point to an increased expansion and activation of ISCs during cholestatic disease after BDL and DDC. This was confirmed by the increased growth of organoids *in vitro* from intestinal crypts isolated from BDL and DDC mice when compared to those isolated from control mice (Fig. 2D and Fig. S3D, respectively).

These results show that activation of senescence in IECs is associated with decreased cell proliferation during human and murine cholestatic disease but increased ISC activation in mice.

### Reduced bile acids and increased bacterial endotoxin contribute to senescence in intestinal cells *in vitro* and *in vivo* during cholestatic liver disease

**Fig. 3:**
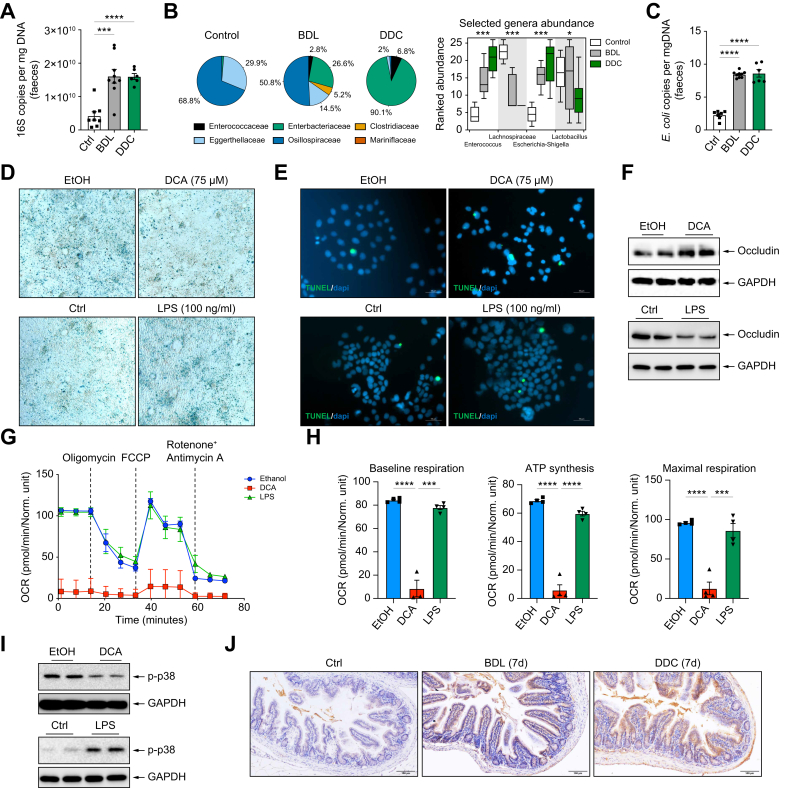
DCA reduces while LPS increases senescence, which is associated with increased OXPHOS in intestinal cells *in vitro*. (A) 16s qPCR in faecal samples from control, BDL and DDC-fed mice (control *vs.* BDL, ∗∗∗*p =* 0.0009; control *vs.* DDC, ∗∗∗∗*p* <0.0001; Brown-Forsythe and Welch one-way ANOVA). (B) Community composition at family level (pie charts) and genus level (boxplot) analysis after 16s rRNA sequencing of faecal samples. (C) qPCR using *E. coli*-specific primers (∗∗∗∗*p* <0.0001; Brown-Forsythe and Welch one-way ANOVA). (D) SA-β-Gal and (E) TUNEL (green) and dapi (blue) staining on CaCo-2 cells incubated with EtOH, EtOH+DCA (75 μM), control and LPS (100 ng/ml) for 24 h. (F) Immunoblotting on protein extracts using anti-occludin and GAPDH antibodies. (G) OCR detection in CaCo-2 cells 24 h after EtOH, DCA and LPS stimulation using Seahorse technology, followed by (H) detailed baseline respiration, ATP synthesis and maximal respiration analyses (∗∗∗∗*p* <0.0001; t-test; EtOH vs DCA; DCA vs LPS; ∗∗∗*p* <0.001; t-test; DCA vs LPS). (I) Immunoblotting showing p38 phosphorylation 24 h after treatments. (J) 4-HNE immunostaining on ileal sections from mice 7 days post BDL and DDC diet. Representative images are shown from 10x magnification. *In vitro* experiments were repeated 2-3x with n = 3-4 replicates. Analyses were done from n = 6-9 mice. Values are mean ± SEM. DCA, Deoxycholic acid; LPS, Lipopolysaccharide; OXPHOS, Oxidative phosphorylation; DDC, 3,5-diethoxycarbonyl-1,4-dihydrocollidine; BDL, Bile duct ligation; 4-HNE, 4-hydroxynonenal.

We hypothesised that intestinal senescence during cholestatic liver disease could be a result of a decreased delivery of bile acids (BAs) and bacterial overgrowth (Fig. 3A) and dysbiosis that occurs in the intestine during murine cholestatic disease in BDL and DDC-fed mice, the latter confirmed by 16s rRNA sequencing. Hence, the microbiome composition in both cholestatic models is dominated by an increase in *Enterobacteriaceae* (Fig. 3B, Table S2) that include potentially pathogenic genera like *Enterococcus* and *Escherichia*, while showing reduced *Lactobacillus* and *Lachnospiraceae* (Fig. 3B). Increased presence of pathogenic *Escherichia coli* (*E. coli*) in BDL and DDC mice was confirmed by qPCR of faecal samples (Fig. 3C).

To test our hypothesis, we performed *in vitro* experiments where we induced senescence in CaCo-2 cells with ethanol treatment for 24 h, as previously described in endothelial cells.^24^ We found that deoxycholic acid (DCA) (75 μM diluted in EtOH) reduced cellular senescence as evidenced by attenuated SA-β-Gal staining (Fig. 3D and Fig. S4A) that was not associated with increased apoptotic cell death (Fig. 3E). Bacterial endotoxin (LPS) promotes senescence in various cell systems including cholangiocytes *in vitro*.^5^ Here, we show LPS can similarly induce senescence in intestinal cells as evidenced by increased SA-β-Gal staining, without impacting on cell death (Fig. 3D,E and Fig. S4B).

Increased senescence in EtOH-treated cells was associated with a reduction in the expression of occludin, whose presence was restored by DCA (Fig. 3F and Fig. S4C). Similarly, LPS-treated cells showed reduced occludin expression compared to controls (Fig. 3F and Fig. S4D), overall suggesting that senescence negatively correlates with intestinal permeability.

Senescence is associated with increased mitochondrial respiration and oxidative stress via p-p38 signalling.^4^^,^^25^ Metabolic analysis using Seahorse technology in CaCo-2 cells showed that senescent EtOH- and LPS-treated cells had significantly higher oxygen consumption rate values compared with DCA-treated cells (Fig. 3G), indicating a significant reduction in mitochondrial respiration, supported by lower basal respiration, ATP synthesis and maximal respiration (Fig. 3H). Immunoblotting evidenced the increased p-p38 expression associated with senescence in EtOH- and LPS-treated cells compared to DCA and control cells (Fig. 3I). Ultimately, 4-HNE immunostaining in intestinal tissues confirmed increased oxidative stress after BDL and DDC feeding compared to control mice (Fig. 3J).

**Fig. 4:**
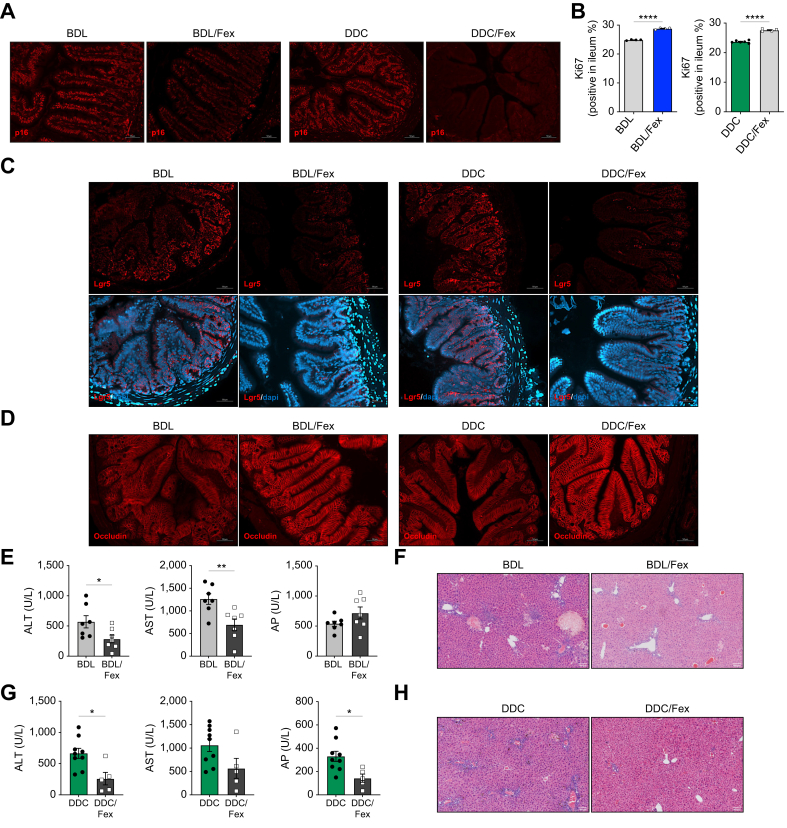
Pharmacological activation of FXR with Fex reduces intestinal senescence and restores intestinal function after BDL and DDC diet. (A) p16 immunofluorescence, (B) quantification of Ki67-positive cells (∗∗∗∗*p* <0.0001), (C) Lgr5 immunofluorescence and (D) occludin immunofluorescence in ileums from Fex/BDL and Fex/DDC. P16, Lgr5 and occludin (red) and dapi (blue). (E, G) Serum transaminases and AP (BDL *vs.* BDL/Fex: ALT, ∗*p =* 0.0412; AST, ∗∗*p =* 0.0071; AP, n.s. = 0.1438. DDC *vs.* DDC/Fex: ALT, ∗*p =* 0.0122; AST, n.s. = 0.093; AP, ∗*p* = 0.0146), and (F, H) H&E staining on liver samples from Fex/BDL and Fex/DDC mice. Analyses were done from n = 5-7 mice. Representative images are shown from 20x (A, C, D) and 10x (E, H) magnification. Values are mean ± SEM. Statistical differences were determined using Welch’s t-test. BDL, Bile duct ligation; 4-HNE, 4-hydroxynonenal; DCA, Deoxycholic acid; DDC, 3,5-diethoxycarbonyl-1,4-dihydrocollidine.

To confirm our observations *in vitro*, we performed *in vivo* studies where we activated intestinal BA signalling with the intestinal-specific FXR agonist fexaramine (Fex) in BDL and DCC models. Treatment with Fex reduced p16 staining in the small and large intestine (Fig. 4A and Fig. S5A,B) from BDL and DDC mice, which was associated with improved Ki67-positive IEC numbers (Fig. 4B). Interestingly, reduced senescence was associated with a lower presence of Lgr5^+^ cells outside the crypt base in BDL/Fex and DDC/Fex mice (Fig. 4C), supporting the association between senescence and Lgr5^+^-ISC expansion we observed (Fig. 2C).

Accordingly with the previously described effects of pharmacological modulation of FXR with Fex in restoring intestinal barrier function,^26^ occludin expression was improved in BDL/Fex and DDC/Fex mice (Fig. 4D and Fig. S5C). Ultimately, supporting previous studies in alcohol-related liver disease (ALD) and cirrhotic mice,^26^^,^^27^ Fex treatment significantly reduced liver injury (necrosis) in BDL and DDC mice (Fig. 4E,F and Fig. S5D,E,G,H) and was associated with reduced liver BA content (Fig. S5F), supporting recovery of FXR signalling in the gut-liver axis.

Next, to determine the impact of intestinal bacteria in regulating senescence, we treated mice with a cocktail of broad-spectrum antibiotics (ABx) for 1 week prior to DDC feeding and for the duration of the experiment (1 week). We confirmed that ABx treatment significantly reduced the presence of p16-positive senescent cells in the small and large intestine of DDC mice (Fig. S6A).

**Fig. 5:**
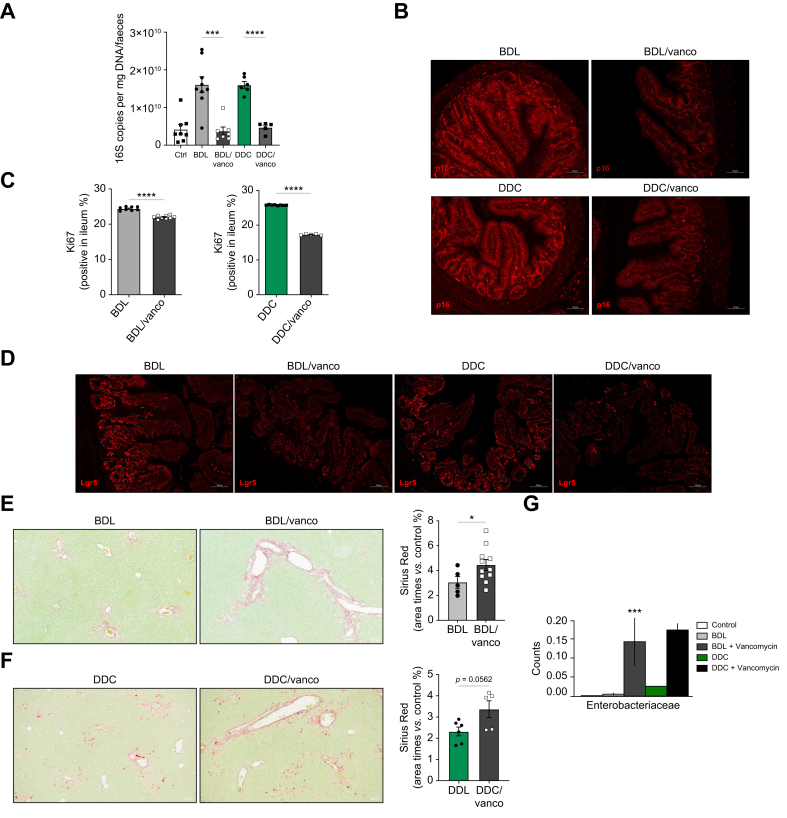
Vancomycin treatment reduces intestinal senescence in mice after BDL and DDC diet. (A) 16s qPCR in faecal samples (BDL *vs.* BDL/vanco, ∗∗∗*p =* 0.0002; DDC *vs.* DDC/vanco, ∗∗∗∗*p* <0.0001; Brown-Forsythe and Welch one-way ANOVA), (B) p16 immunofluorescence, (C) quantification of Ki67-positive cells (BDL *vs.* BDL/vanco, ∗∗∗∗*p <*0.0001; DDC *vs.* DDC/vanco, ∗∗∗∗*p* <0.0001; Welch’s t-test) (D) Lgr5 immunofluorescence on ileal samples from BDL and DDC-fed mice pre-treated with vancomycin (50 mg/kg) for 1 week before intervention and throughout the experiment. (E, F) Sirius red staining and quantification in livers (BDL *vs.* BDL/vanco, ∗*p =* 0.0442; DDC *vs.* DDC/vanco, n.s. *p* = 0.0562; Welch’s t-test) (G) Community composition at genus level from 16srRNA sequencing of faecal samples. Analyses were done from n = 5-11 mice. Representative images are shown from 20x (B, D) and 10x (E, F) magnification. BDL, Bile duct ligation; 4-HNE, 4-hydroxynonenal; DDC, 3,5-diethoxycarbonyl-1,4-dihydrocollidine.

In parallel, we performed *in vivo* experiments using vancomycin, a non-absorbable antibiotic that significantly reduced the presence of intestinal bacteria (Fig. 5A) and was associated with a reduction of senescence in the intestines from BDL/vancomycin and DDC/vancomycin mice (Fig. 5B and Fig. S6B), in agreement with our results in ABx-treated mice. Decreased senescence of IECs was associated with a further reduction in their proliferative capacity (Fig. 5C and Fig. S7A), supporting the key role of BA signalling in preserving intestinal cell function. Reduced Lgr5 expression (Fig. 5D and Fig. S7B) in the small intestine from BDL/vancomycin and DDC/vancomycin mice further supported the positive correlation between senescence and ISC stemness that was confirmed by the reduced organoid growth capacity of crypt base cells isolated from DDC/vancomycin mice (Fig. S7C). Ultimately, vancomycin significantly aggravated liver fibrosis in mice after BDL and DDC-feeding (Fig. 5E,F), an effect that was also observed in DDC/ABx mice (Fig. S8A) in accordance with previous studies,^28^ where increased disease progression in antibiotic-treated mice correlated with an increased abundance of pathogenic *Enterobacteria*, as we observed in BDL/vancomycin and DDC/vancomycin mice (Fig. 5G).

Overall, our results demonstrate the absence of BA-FXR signalling and the increased presence of bacterial endotoxin as mechanisms mediating senescence in IECs during cholestatic liver disease.

### Elimination of senescent cells exacerbates liver injury and fibrosis during cholestatic disease in mice and is associated with disruption of intestinal barrier integrity

Previous studies have shown increased senescence in cholangiocytes during cholestatic disease^5^^,^^6^ and suggested the elimination of senescence as a potential therapeutic approach.^13^^,^[15], [16], [17], [18] Our results showing that the overall expression of p16 was higher in the intestinal epithelia compared to the liver from BDL and DDC mice (Fig. S9A), where senescence was mainly restricted to cholangiocytes (Fig. S9B), support the intestine as a relevant organ targeted by systemic senolytic treatments.

To test the effects of elimination of senescence in the gut-liver axis during cholestatic disease, we used p16-3MR mice in which p16-expressing cells are killed by ganciclovir (GCV) treatment.^29^ In our initial experiments, GCV-treated p16-3MR mice showed high mortality, particularly at day 6 after BDL (data not shown). The surviving BDL/GCV mice showed profuse necrosis and increased fibrosis in the liver at 7 days after surgery (Fig. S10A and B).

To confirm the detrimental effects of elimination of p16-senescent cells during cholestatic disease we performed additional experiments with p16-3MR mice treated with GCV, which we culled 5 days after BDL, to reduce mortality and increase our sample size.

Firstly, we confirmed GCV treatment was not cytotoxic to the liver, as demonstrated by the absence of changes in serum transaminase and alkaline phosphatase levels (Fig. S11A) and caspase 3 activity on whole liver extracts from p16-3MR mice (Fig. S11B), which showed normal histology and lack of fibrosis, as evidenced by H&E and Sirius Red staining on liver sections (Fig. S11C–E).

**Fig. 6:**
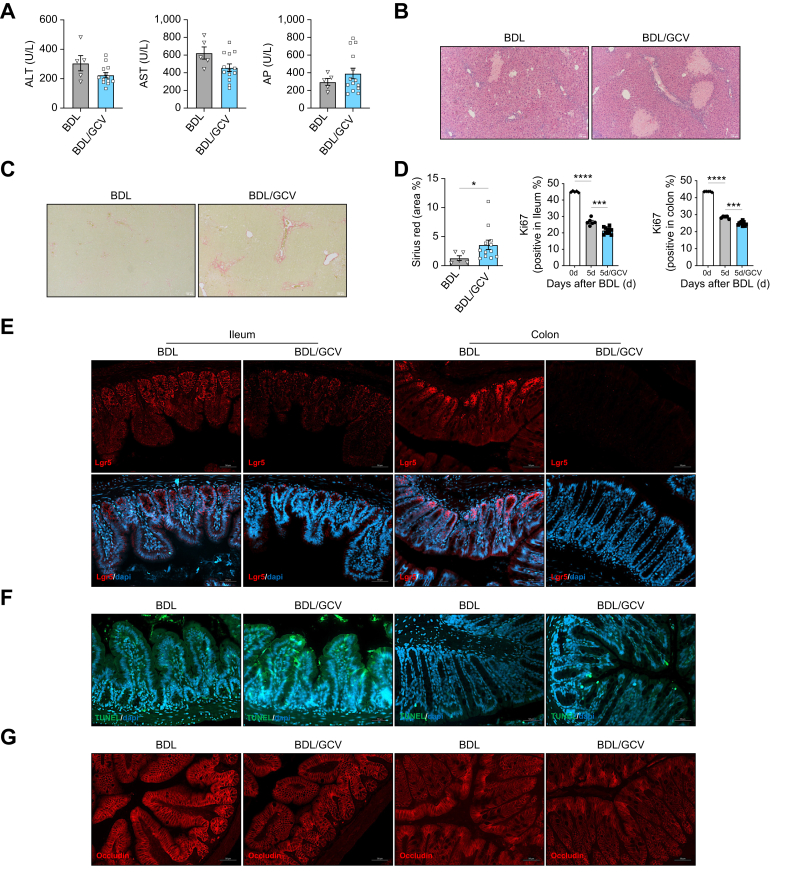
Elimination of senescent cells exacerbates liver injury and fibrosis that is associated with IEC death and reduced tight junction protein expression during murine cholestatic disease. (A) Serum transaminase and AP (BDL *vs.* BDL/GCV: ALT, *p =* 0.2036; AST, *p =* 0.0814; AP, *p =* 0.2083; Welch’s t-test), (B) H&E, (C) Sirius Red staining and quantification of positively stained area on liver sections from p16-3MR mice at 5 days post BDL and BDL/GCV (25 mg/kg) treatment (BDL *vs.* BDL/GCV, ∗*p =* 0.0268; Welch’s t-test). (D) Quantification of Ki67-positive cells (0 days *vs.* BDL 5 days, ∗∗∗∗*p <*0.0001; BDL 5 days *vs.* BDL 5 days/GCV, ∗∗∗*p <*0.001; Brown-Forsythe and Welch one-way ANOVA). (E) Lgr5^+^ immunofluorescence, (F) TUNEL assay and (G) Occludin immunofluorescence in ileum and colon from BDL and BDL/GCV mice. Analyses were done from n = 5-13 mice. Representative images are shown from 10x (B, C) and 20x magnification. BDL, Bile duct ligation; 4-HNE, 4-hydroxynonenal.

After BDL, whilst no significant changes in serum transaminases or alkaline phosphatase levels were observed (Fig. 6A), we found significantly increased liver necrosis and fibrosis in BDL/GCV mice compared with BDL animals (Fig. 6B,C and Fig. S11F).

Analysis of intestinal samples in BDL/GCV mice showed that elimination of senescent cells (Fig. S12A) was associated with a further reduction in IEC proliferation (Fig. 6D and Fig. S12B), pointing to an impaired repair response to damage. Accordingly, we found decreased expression of Lgr5^+^ in BDL/GCV mice (Fig. 6E) and a reduced growth capacity of crypt base cells, as all cysts were dead at day 6 of culture (data not shown). Increased apoptosis (Fig. 6F) and reduced expression of occludin, which relocated from the basal to the intercellular apical membrane (Fig. 6G and Fig. S12C), were evident in the small and large intestine in BDL/GCV mice. Analysis of circulating LPS-binding protein confirmed the increased intestinal leakiness in BDL/GCV mice compared to BDL animals (Fig. S12D).

Overall, our results point to a detrimental impact of eliminating senescent cells on the gut-liver axis, with reduced intestinal reparative capacity and increased leakiness, which could contribute to the observed aggravation of liver damage and fibrosis.

### Elimination of senescent cells with the senolytic ABT-263 exacerbates liver injury and fibrosis and is associated with disruption of intestinal barrier integrity during cholestatic disease in mice

**Fig. 7:**
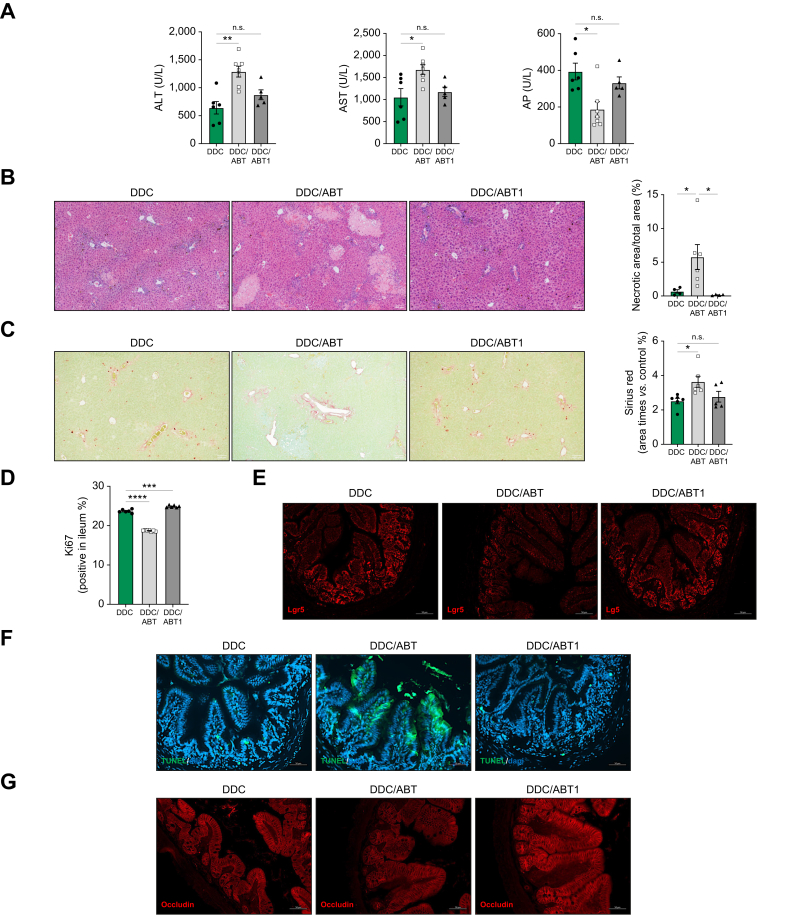
Persistent elimination of senescent cells after ABT-263 treatment aggravates liver damage and fibrosis after DDC diet. (A) Serum transaminases and AP (DDC *vs.* DDC/ABT: ALT, ∗∗*p =* 0.0030; DDC *vs.* DDC/ABT1: ALT, n.s. *p =* 0.2542; DDC *vs.* DDC/ABT: AST, ∗*p =* 0.0497; DDC *vs.* DDC/ABT1: AST, n.s. *p =* 0.8317; DDC *vs.* DDC/ABT: AP, ∗*p =* 0.0143; DDC *vs.* DDC/ABT1: AP, n.s. *p =* 0.5013), (B) H&E and quantification of the percentage of necrotic areas (DDC *vs.* DDC/ABT, ∗*p =* 0.0410; DDC *vs.* DDC/ABT1, ∗*p =* 0.0295), (C) Sirius Red staining and quantification of percentage of positive area on liver sections (DDC *vs.* DDC/ABT, ∗*p =* 0.0346; DDC *vs.* DDC/ABT1, n.s. *p =* 0.7464), (D) Quantification of Ki67-positive cells (DDC *vs.* DDC/ABT, ∗∗∗∗*p* <0.0001; DDC *vs.* DDC/ABT1, ∗∗∗*p* = 0.0007) (E) Lgr5 immunofluorescence, (F) TUNEL assay and (G) occludin immunofluorescence on ileal samples. All from DDC-fed mice treated with ABT-263 from day 1 to the end of the experiment (7 days; DDC/ABT) or only at day 1, 2 and 3 of DDC feeding (DDC/ABT1). Analyses were done from n = 5-7 mice. Representative images are shown from 10x (B, C) and 20x magnification. Values are mean ± SEM. Statistical differences were determined using Brown-Forsythe and Welch one-way ANOVA. DDC, 3,5-diethoxycarbonyl-1,4-dihydrocollidine.

Next, we evaluated the effects of the senolytic drug ABT-263 (Navitoclax), a Bcl-2, Bcl-XL inhibitor that eliminates senescent cells, on the gut-liver axis during cholestatic disease in two murine models. While ABT-263 treatment alone had no effects on liver injury and cell death or fibrosis (Fig. S13A–E), the elimination of senescence with ABT-263 (from day 1) in combination with DDC feeding (for 7 days; DDC/ABT) led to a significant increase in liver injury (Fig. 7A,B) and fibrosis (Fig. 7C), in line with our results in BDL/GCV mice.

Interestingly, the administration of ABT-263 for only the initial 3 days of DDC feeding (DDC/ABT1) allowed for restoration of senescence in the intestine (Fig. S14A) and was associated with a significant reduction in liver injury (Fig. 7A,B) and fibrosis (Fig. 7C) compared to DDC/ABT mice.

Further analyses confirmed the significant reduction in IEC proliferation in the small intestine of DDC/ABT compared to DDC and DDC/ABT1 mice (Fig. 7D and Fig. S15A). In accordance with our previous observations, DDC/ABT mice showed a reduced expression of Lgr5 (Fig. 7E and Fig. S15B) and profuse cell apoptosis (Fig. 7F), leading to cell shedding (Fig. S15C) and downregulation of occludin in the small intestine (Fig. 7G and Fig. S15D) compared to DDC and DDC/ABT1 mice. Analysis of LPS-binding protein in serum samples supported increased intestinal permeability in DDC/ABT mice (Fig. S15E) and increased presence of *E. coli*, although not reaching statistical significance (Fig. S15F).

**Fig. 8:**
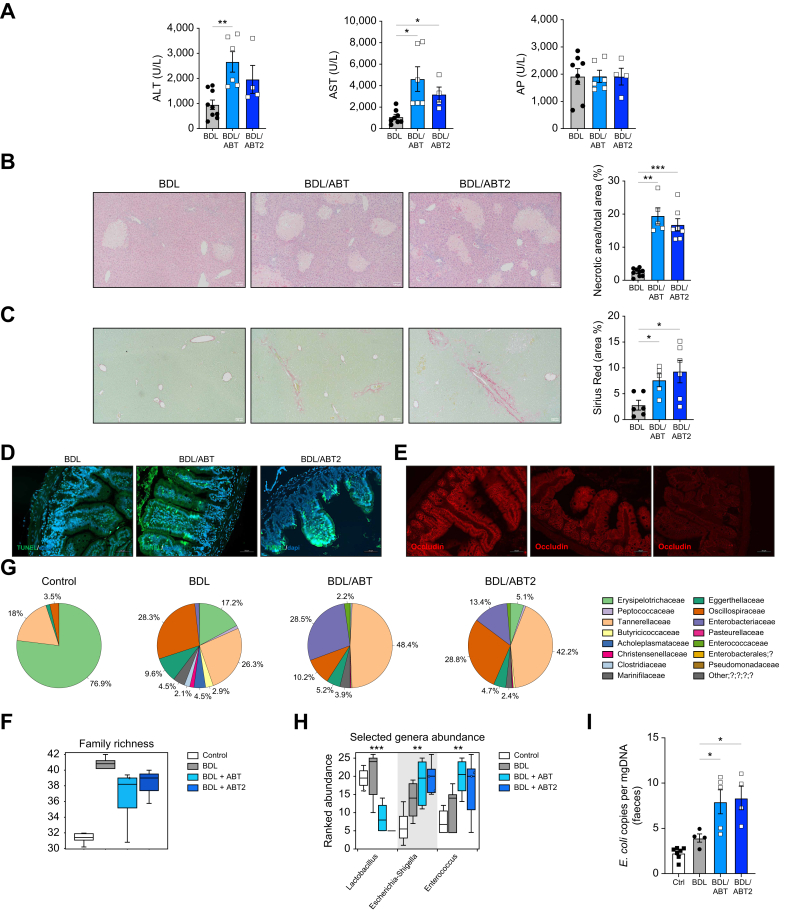
Elimination of senescent cells with the senolytic ABT-263 at later stage exacerbates liver injury and fibrosis after BDL. (A) Serum transaminases and AP (BDL *vs.* BDL/ABT: ALT, ∗∗*p =* 0.0060; BDL *vs.* BDL/ABT2: ALT, n.s. *p =* 0.1626; BDL *vs.* BDL/ABT: AST, ∗*p =* 0.0250; BDL *vs.* BDL/ABT2: ALT, ∗*p =* 0.0476; BDL *vs.* BDL/ABT: AP, n.s. *p =* 0.9900; BDL *vs.* BDL/ABT2: AP, n.s. *p =* 0.9912), (B) H&E and quantification of the percentage of necrotic areas (BDL *vs.* BDL/ABT, ∗∗*p =* 0.0017; BDL *vs.* BDL/ABT2, ∗∗∗*p =* 0.0002) (C) Sirius Red staining and quantification of percentage of positive area (BDL *vs.* BDL/ABT, ∗∗*p =* 0.0039; BDL *vs.* BDL/ABT2, ∗∗∗*p =* 0.0002) on liver sections, (D) TUNEL assay and (E) occludin immunofluorescence on ileal sections. (F) Community richness and (G) composition at family and (H) genus level analysis after 16s rRNA sequencing of faecal samples. (I) qPCR analysis detecting *E. coli* (BDL *vs.* BDL/ABT, ∗*p =* 0.0372; BDL *vs.* BDL/ABT2, ∗*p* = 0.0364) Analyses were done from n = 5-7 mice at 7 days for BDL, BDL/ABT and BDL/ABT2 treatment. Representative images are shown from 10x (B, C) and 20x magnification (D, E). Values are mean ± SEM. Statistical differences were determined using Brown-Forsythe and Welch one-way ANOVA. BDL, Bile duct ligation; 4-HNE, 4-hydroxynonenal.

Further *in vivo* studies using the BDL surgical model supported our observations in DDC/ABT mice, showing that persistent ABT-263 treatment from day 1 after BDL (BDL/ABT) led to increased liver injury (Fig. 8A,B) and fibrosis (Fig. 8C), as evidenced by an increased percentage of necrotic and Sirius Red-stained areas on liver sections.

Patients generally present to the clinic with established disease; thus, we tested the effects of the elimination of senescence from day 4 after BDL up to the duration of the experiment (7 days; BDL/ABT2). We found that later-senolytic treatment led to increased serum transaminases, profuse necrosis and increased fibrosis in BDL/ABT2 mice (Fig. 8A-C).

Further analyses confirmed that the elimination of senescent cells (Fig. S16A) with both treatment regimens led to the significant reduction of IEC proliferation in the small and large intestine of BDL/ABT mice (Fig. S16B), while the reduction in colonic IEC proliferation was less pronounced in BDL/ABT2 and higher in the small intestine compared to BDL/ABT (Fig. S16B). Both BDL/ABT and BDL/ABT2 groups showed reduced expression of Lgr5 (Figs. S16C and S17A), profuse cell apoptosis (Fig. 8D and Fig. S17B) and downregulation of occludin (Fig. 8E and Fig. S17C) in the small and large intestine. Lastly, we found that the elimination of senescent cells in BDL/ABT and BDL/ABT2 mice was associated with further intestinal bacterial growth (Fig. S17D) and a decrease in richness (Fig. 8F). Overall community composition was typical for mice, with a significant increase in *Enterobacteriaceae* and *Enterococcaceae* (Fig. 8G and Fig. S17E), both prevalent in PSC and murine cholestatic disease.^28^^,^^30^ At the genus level we could observe a decrease in *Lactobacillus* and an increase of *Escherichia* and *Enterococcus* (Fig. 8H, Table S3), reported to be enriched during cholestatic disease and associated with intestinal dysfunction.^28^^,^^30^ Further qPCR analysis confirmed the increased presence of *E. coli*, a pathobiont contributing to cholestatic disease progression,^28^ in BDL/ABT and BDL/ABT2 faecal samples compared to control and BDL samples (Fig. 8I).

Overall, our results show that pharmacological elimination of senescent cells hampered intestinal cell repair capacity, increased cell death, promoted barrier dysfunction and increased the presence of pathobionts, aggravating liver injury and fibrosis during cholestatic disease. These data suggest that whilst the use of the senolytics in liver disease may seem attractive from a liver perspective, they could have wider potentially detrimental consequences via the gut-liver axis.

## Discussion

Herein, we show that senescence is increased in IECs during cholestatic disease in patients with PSC and in two preclinical murine models, where senescence was associated with a reduced intestinal tissue repair capacity. Mechanistically, we show that the reduction in BAs and downstream FXR signalling along with the increased presence of bacteria promote intestinal senescence during cholestatic disease. Finally, we demonstrate that the genetic and pharmacological elimination of senescent cells has a detrimental impact on the gut-liver axis, hampering epithelial regeneration, promoting IEC death and increasing permeability, which are associated with aggravated liver injury and fibrosis.

Senescence is a cellular response to stress and damage that prevents cell death and growth of damaged cells,^1^ improving tissue renewal and repair,^2^ while being detrimental when persistent or unresolved. Senescent cells accumulate in patients with primary biliary cholangitis^3^^,^^4^^,^^7^ and PSC,^3^^,^^5^^,^^6^^,^^8^ where senescence is associated with poor prognosis.^3^^,^^8^^,^^9^ Still, the role of senescence during liver disease is complex and reportedly cell-specific, having an antifibrotic effect in HSCs,[11], [12], [13]^,^^31^^,^^32^ while being detrimental when active in cholangiocytes.^5^^,^[12], [13], [14], [15], [16]

Herein, we demonstrate that senescence is increased in IECs of the small and large intestine, mainly in enterocytes/colonocytes, during human and murine cholestatic disease. We propose that the activation of senescence is a repair response to stress aimed at preserving the intestinal epithelia from death and transformation that, when persistently activated, ultimately leads to reduced proliferative/regenerative response and the loss of tight junction protein expression.

Mechanistically, we demonstrated that the reduction in BAs along with the increased presence of bacteria in the intestine during cholestatic liver disease are contributors to IEC senescence.

Our results *in vitro* in intestinal cells, showing that BA treatment reduced senescence and modulated mitochondrial respiration, parallel the observations by *Gee et al.*, who showed that obeticholic acid attenuated senescence in the brain during cholestatic disease, which was associated with a metabolic shift and reduced mitochondrial respiration.^33^ Our *in vivo* studies using Fexaramine, an intestinal-restricted pharmacological FXR agonist, confirmed the negative association between BA signalling and senescence that we observed in mice after BDL and DDC. Our results support the previously reported role of the BA-FXR in preserving intestinal barrier function and protecting mice from liver injury during cholestatic liver disease,^34^ cirrhosis and ALD.^26^^,^^27^ Yet, establishing the role of senescence as a response to intestinal stress in the broader context of liver disease (*e.g*. ALD, cirrhosis) requires further investigation.

Cholestatic liver disease is associated with gut bacteria overgrowth and profound changes in the microbiome composition in humans and rodents, including the increase of pathobionts (*i.e. Enterobacteria/E. coli)* that actively contribute to disease progression.^28^^,^^35^^,^^36^ We propose that increased exposure of the intestinal epithelia to microbes during cholestatic disease could be a mechanism mediating IEC senescence. Our results in CaCo-2 cells exposed to LPS showing increased senescence, together with our studies in mice treated with vancomycin or a cocktail of antibiotics showing reduced senescence, confirmed the role of the intestinal microbiome in promoting intestinal senescence during cholestatic disease. Bacterial endotoxin promotes oxidative stress and senescence in various cell systems including cholangiocytes *in vitro*.^5^ Additionally, increased mitochondrial respiration and reactive oxygen species production via activation of p-p38 are potent inducers of senescence,^25^ including in biliary epithelial cells,^4^ which could be mediators of microbe-induced senescence.

Furthermore, we demonstrate that increased senescence in IECs during cholestatic liver disease hampers intestinal regeneration, while our results support a positive crosstalk between senescence and ISC stemness. During homeostasis, the intestinal epithelia regenerate every 2-5 days through a coordinated self-renewal and differentiation of Lgr5^+^-ISCs into absorptive (enterocytes) or secretory (goblet cells) lineages that repopulate the epithelia as cells migrate up the villi,^21^^,^^22^ ensuring integrity of intestinal (barrier) function. After injury, intestinal regeneration is achieved through profound ISC reprogramming or dedifferentiation of IECs (to expressing Lgr5+) that mediate restoration of the intestinal epithelium after injury.^22^ Dedifferentiation and reprogramming are key events in tissue repair after injury and p16-mediated senescence promotes reprogramming in response to injury *in vivo*.^37^ Our results showing an increase in Lgr5+ cells after BDL and DDC during cholestatic disease point to increased ISC stemness. This was supported by the improved capacity of crypt ISCs isolated from cholestatic mice to generate organoids *in vitro* that was otherwise reduced/impaired when using ISCs isolated from DDC/vancomycin and DDC/p16-3MR/GCV mice, where senescence was eliminated. Still, increased ISC stemness capacity/dedifferentiation during cholestatic liver disease did not correlate with recovery of IEC proliferation as these cells were senescent in response to absence of BAs and increased presence of bacteria. The negative correlation between IEC senescence and proliferation was confirmed in Fex-treated mice, where reduced senescence associated with restored IEC proliferative capacity, independently from ISC activation. The further reduction of IEC proliferation in vancomycin-treated mice despite reduction of senescence may be explained by the lack of BA-FXR signalling in the intestine.

Based on the detrimental role of senescence in cholangiocytes during cholangiopathies, some studies have tested the potential therapeutic effects of eliminating senescence using genetic or pharmacological (senolytics) approaches. Downregulation of p16 and reduction of senescence in the biliary epithelia^15^^,^^17^^,^^18^ and senolytics including A-1331852, a Bcl-XL-specific inhibitor, and the flavonoid Fisetin led to reduced senescence of biliary cells and fibrosis.^16^ The senolytic ABT-263 (Navitoclax) was also used to target senescent fibroblasts *in vitro*, while its efficacy *in vivo* was not tested.^16^

Approaches to eliminate senescence systemically in the treatment of cholangiopathies may have limitations due to the differential roles of senescence in different liver cell types during cholestatic liver disease, being detrimental in cholangiocytes,^5^^,^[12], [13], [14], [15], [16] but anti-fibrotic when activated in HSCs.[11], [12], [13] Previous studies showed that therapeutic approaches to attenuate cholestasis-related fibrosis in mice by reducing senescence had efficacy when targeting different cell types.^12^^,^^13^ Our results showing increased senescence in IECs during cholestatic disease add another layer of complexity to the application of therapeutics, where senescence is regulated systemically during cholestatic liver disease. Indeed, we showed that elimination of senescent cells using systemic genetic (p16-3MR mice) and pharmacological (senolytic) approaches led to aggravated liver damage and fibrosis, supporting the detrimental effects of systemic elimination of senescence cells during cholestatic disease. Previous work by Fickert *et al.*^38^ described the detrimental role of ursodeoxycholic acid in mediating liver tissue damage by increasing bile infarct and necrosis via disruption of biliary tree branches due to increased biliary pressure and bile leakage, which may also contribute to profuse presence of bile infarcts in BDL and DDC-fed mice after elimination of senescence.

Elimination of senescence was associated with loss of the intestinal barrier. We propose the profuse IEC death, causing cell shedding from the epithelia and leading to loss of tight junction protein-expressing cells after elimination of senescent cells, as potential mechanisms mediating increased permeability. Moreover, the changes we observe in the intestinal microbiome in mice after senolytic treatment that include a further increase in pathogenic bacteria like *E. coli*, with well-established effects in disrupting gut barrier function,^28^^,^^30^ may also contribute to the loss of barrier function and cholestatic disease progression.

The impact of senolytic drugs on the gut microbiome remains largely undefined.^39^ Previous work showed that the flavonoid quercetin alone and in combination with the tyrosine kinase inhibitor dasatinib, modulate the microbiome of old mice.^40^ Nonetheless, given the dietary component of these plant-derived flavonoids, it is difficult to dissect the direct effects of flavonoids on microbiome composition from the indirect effects mediated by their senolytic activity. Here we show that elimination of senescent cells with navitoclax promotes bacterial overgrowth and significant changes in microbiome composition, with an increase in pathogenic bacteria (*i.e*. *E. coli*), which points to a direct association between regulation of intestinal senescence and the gut microbiome. The mechanisms mediating the association between senescence and changes in the microbiome may involve the regulation of Paneth cells, the main antimicrobial IEC, via the crosstalk between senescence and ISCs as suggested by our preliminary observations showing attenuated lysozyme expression when senescence and ISC activation are reduced (not shown). To demonstrate this is beyond the scope of this study and warrants future work.

Overall, our results contribute to improve our fundamental understanding of the mechanisms regulating intestinal barrier function during cholestatic liver disease. Here, we show that intestinal senescence is activated as a stress response to the reduction of BAs and increased presence of LPS in the intestine that would protect IECs from death during cholestatic liver disease. Our work highlights the differential role of senescence in relevant cell types in the gut-liver axis, which should be considered when proposing senolytics as therapeutic approaches to treat cholestatic disease. Our current study suggests that future therapeutic approaches aiming at the elimination of senescent cells should be delivered in a cell-specific manner rather than systemically. Also, previous studies proposed that senolytics could be more effective and reduce side effects of long-term treatments if given intermittently.^41^ Herein, we tested this and found that persistent or late elimination of senescent cells had detrimental effects during cholestatic disease, while short senolytic treatment at early stages of the disease, allowing for the recovery of intestinal senescence, reduced liver damage and fibrosis compared to persistent treatment, but was still ineffective at alleviating liver injury and fibrosis. Future therapeutic approaches aimed at regulating intestinal senescence during cholestatic disease may include the modulation of the microbiome to control intestinal senescence and hence tissue repair and barrier function.

## Abbreviations

Abx, broad-spectrum antibiotics; ALD, alcohol-related liver disease; BAs, bile acids; BDL, bile duct ligation; DCA, deoxycholic acid; DDC, 3,5-diethoxycarbonyl-1,4-dihydrocollidine; Fex, fexaramine; IECs, intestinal epithelial cells; HSCs, hepatic stellate cells; ISCs, intestinal stem cells; LPS, lipopolysaccharide; PSC, primary sclerosing cholangitis; WT, wild-type.

## Conflict of interest

All authors declare that they have no competing financial interests with respect to this manuscript.

Please refer to the accompanying ICMJE disclosure forms for further details.

## Authors’ contributions

MMG, KH, PR, GB, JL, AP, CB, AAA, PLM, ES, MP, DB, FH, SAR, NB performed experiments and data analysis. NB, SAR designed and supervised experiments. MMG, NB and PR performed animal experiments. NB, MMG, KH, GB and PR performed sample processing and histological analyses. MMG performed *in vitro* experiments. AP and JL performed organoids experiments and image processing. FSPN, ES and FH performed bioinformatics analysis. MPh performed LC/MS and data analysis. MPa recruited, consented patients and analysed human samples. PR and GB analysed human samples. NB wrote the manuscript. SAR, SMR and JL critically revised the manuscript. NB, SMR and SAR conceived the study. NB, SAR, FH obtained funding.

## Data availability statement

The authors declare that all data generated from this study are available within the manuscript and the supplemental material provided. Any additional files or information can be provided upon request to the corresponding authors.
