# Supplementary material for: Regulation of intestinal senescence during cholestatic liver disease modulates barrier function and liver disease progression
Source: JHEP Rep. 2024 Jun 29;6(10):101159. doi: 10.1016/j.jhepr.2024.101159 (PMC11418120; doi:10.1016/j.jhepr.2024.101159)
Supplement: Multimedia component 1 [file mmc1.pdf]

Regulation of intestinal senescence during cholestatic liver disease modulates barrier function and disease progression

Mar Moreno-Gonzalez, Katherine Hampton, Paula Ruiz, Gemma Beasy, Falk SP Nagies, Aimee Parker, James Lazenby, Caitlin Bone, Ane Alava-Arteaga, Meha Patel, Charlotte Hellmich, Pablo Luri-Martin, Ece Silan, Mark Philo, David Baker, Simon M Rushbrook, Falk Hildebrand, Stuart A Rushworth, Naiara Beraza

Table of contents

Supplementary materials and methods 2

Supplementary figures...10

Supplementary figure legends..27

Supplementary tables…………………...………………………………….…………….....33

Supplementary references………………...………………………………………………73

Supplementary materials and methods

Experimental procedures in animals

All experimental procedures were performed in 8–12-week-old male mice at the Disease Modelling Unit (University of East Anglia, UK). All experiments were approved by the Animal Welfare and Ethical Review Body (AWERB, University of East Anglia, Norwich, UK). All procedures were carried out following the guidelines of the National Academy of Sciences (National Institutes of Health, publication 86-23, revised 1985) and were performed within the provisions of the Animals (Scientific Procedures) Act 1986 (ASPA) and the LASA Guiding Principles for Preparing for Undertaking Aseptic Surgery (2010) under UK Home Office approval (PP9417531).

Cholestasis was induced by ligating the common bile duct (bile duct ligation; BDL) as previously described[1] and fed a diet containing 0.1% 3,5-diethoxycarbonyl-1,4-dihydrocollidine (DDC) ad libitum for 1 week[1].

P16-3MR transgenic mice[2] were kindly provided by UNITY Biotechnology, Inc. (USA).

We performed BDL in P16-3MR transgenic mice and from day 1, GCV (25 mg/kg) (Sigma Aldrich - PHR1593) was injected daily via i.p. until termination of the studies (5 d or 7 d; BDL/GCV).

Groups of mice were treated with the intestine-restricted FXR pharmacological agonist Fexaramine (100 mg/kg) by daily oral gavage in corn oil from day 1 after BDL or 0.1% DDC-feeding until the completion of the experiments (7days).

Some groups of mice received a cocktail of broad-spectrum antibiotics: ampicillin (1g/L) in drinking water, and vancomycin (50 mg/kg), neomycin (100 mg/kg) and metronidazole (100 mg/kg) by oral gavage. This regimen was started at 1 day before BDL or 0.1%DDC feeding and continued daily throughout the duration of the experiment (7 days).

The intestine-restricted and non-absorbable vancomycin was administered (0.5 g/L in drinking water) to some groups of mice 1 week before BDL or 0.1%DDC feeding and throughout the duration of the experiments (7 days).

Another cohort of mice undergoing BDL or being fed with 0.1% DDC diet received ABT-263 (50 mg/kg/day) daily by oral gavage either from day 1 post intervention (7 d; BDL/ABT, DDC/ABT); only at day 1, 2 and 3 after initiation of DDC feeding (DDC/ABT1), or from day 4 post BDL until the completion of the experiment (BDL/ABT2).

Human intestinal tissue samples

Paraffin embedded and cryo-preserved colonic tissue from PSC patients obtained from the Norwich Research Park (NRP) BioRepository, Norwich and Norfolk University Hospital NHS foundation trust. (reference 19/EE/0089, East of England - Cambridge East Research Ethics Committee). Samples from control volunteers were obtained from the MOTION study, reviewed and agreed by the Human Research Governance Committee at the Quadram Institute Bioscience and the East Midlands - Nottingham 1 Research Ethics Committee (reference 19/EM/0055)

The use of PSC human tissue samples was approved by the Faculty of Medicine and Health Sciences Research Ethics committee (University of East Anglia, Norwich, UK), the NRP Biorepository Access Committee. Use of MOTION samples was approved by the Human Research Authority on the 1st April 2019. IRAS project ID number 241617. Collection and handling of human samples used in this study conformed to the Declaration of Helsinki and the Human Tissue Act (UK) and Good Clinical Practice Guidelines (UK). All samples were collected from patients and volunteers after informed consent was signed.

Determination of liver damage

Alanine aminotransferase (ALT), aspartate aminotransferase (AST) and alkaline phosphatase (AP) were determined in mouse serum using the Randox Daytona analyser (Daytona) following the manufacturer's instructions as we previously described[1].

Histology, immunohistochemistry and immunofluorescence

Liver and gut tissues were fixed in 10% neutral buffered formalin (Sigma – HT501128-4L), embedded in paraffin and sectioned. For pathological analysis, liver sections were dewaxed, hydrated and stained for histopathological analysis using haematoxylin and eosin (H&E) to determine tissue structure and cell infiltration and sirius red to stain collagen and detect liver fibrosis. Slides were imaged using brightfield on a BX53 upright microscope (Olympus) with an Olympus DP74 colour camera and a pT100 LED transmitted light source (CoolLED). 5-10 fields of view per sample were imaged and analysed using open source FIJI software[3] as we described

previously [1, 4] to detect and quantify collagen in the sample. Fibrosis is represented as the percentage of stained area relative to total area per field.

Immunohistochemistry (IHC) was carried out in small and large intestine paraffin embedded tissue slides using an anti-Ki67 (Abcam – AB15580), anti-4HNE (Abcam – AB46545) and anti-p16 (Abcam – AB54210) antibody diluted in antibody diluent (Dako – S0809), after sodium citrate antigen retrieval. Next, we used EnVision$^+$polymer HRP labelled anti-mouse (Dako – K4001) or anti-rabbit (Dako - K4003) and IHC was developed using the DAB$^+$ chromogen system (Dako - K3468) after which nuclei were counterstained with hematoxylin. Slides were imaged on a BX53 and analysed using FIJI as described above.

Sodium citrate antigen retrieval was also used for IF in paraffin embedded sections using an anti-p16 (Abcam – AB54210), anti-Occludin (Abcam – AB216327) and anti-Lgr5 antibody (Thermo Fisher – MA5-25644). Apoptotic cells were labelled using terminal deoxynucleotidyl transferase dUTP nick end labeling (TUNEL) assay (Roche - 11684809910) on small and large intestine paraffin sections following manufacturer's instructions. Slides were mounted with a DAPI-mounting solution (Vector Laboratories – H-1200) to stain cell nuclei. Fluorescent microscopic imaging was performed using an AxioImager M2 (Zeiss) with the AxioCam mRM monochrome camera and standard light source and filter sets supplied. Images were analysed using the ZenBlue Software (Zeiss) and or FIJI[3].

RNA isolation and Quantitative Real-Time PCR

RNA was isolated from liver samples or cell cultures using QiAzol lysis Reagent (Qiagen – 79306). The first strand synthesis and reverse transcription was performed using M-MLV Reverse Transcriptase (Invitrogen – 28025013). Quantitative Real-Time PCR (qPCR) was carried out using SYBR Green reagent (Life Technologies – 10187094) using the ViiA7 Real-time PCR detection system (Applied Biosystems). Gene expression was normalized to TATA-box binding protein 1 (TBP1) and is represented in times versus control sample gene expression. Primer sequences can be provided upon request.

In vitro experiments

Human CaCo-2 cells were cultured in RPMI 1640 media (Sigma - R8758) supplemented with 10% FBS, 1% glutamine (Sigma – G7513) and 1% Pen/Strep (Lonza – DE17-602E) for up to 40 passages changing the medium every 3 days with one passage per week. Cells were treated with either 1 µl 100% ethanol, 100 ng/mL Lipopolysaccharides (LPS) from *Escherichia coli* O55:B5 (Sigma Aldrich – L2637) or 75 µM Deoxycholic acid (DCA) (Sigma Aldrich – D2510-10G) for 24 hours.

Western blot analysis

Proteins were extracted from cultured CaCo-2 cells using RIPA buffer containing 50 mM Tris-HCL, 150 mM NaCl, 0.1% SDS, 2 mM EDTA, 5% sodium deoxycholate, 1% Igepal 630, 1 mM PMSF and protease inhibitor (Sigma Aldrich - 4693124001). Proteins were resolved using sodium dodecyl sulfate-polyacrylamide gels and transferred to nitrocellulose membranes (Whatman - WHA1541185). Membranes were probed with p-p38 (Cell Signaling Technologies - 9211) and Occludin (Abcam - AB216327). GAPDH was used as loading control. Anti-rabbit IgG-HRP-linked (Cell Signaling Technologies – 7074S) or anti-mouse IgG-HRP linked (Cell Signaling Technologies – 7076S) were used as secondary antibodies. Images of the blots were taken using ChemiDoc MP imaging system (Bio-Rad).

Seahorse assay

CaCo-2 cells were harvested by trypsinization (0.25% trypsin in EDTA, Sigma Aldrich- T3924) and seeded into a 96-well Seahorse cell culture plate (Agilent – 101085-004) at a density of 7.5 x 10^4 cells per well. Cells were incubated at 37°C at 5% CO_2 for 48 hours and washed with PBS (Sigma Aldrich – D8537) before the addition of the treatments. Cells were treated with either 1 µl 100% ethanol, 100 ng/mL Lipopolysaccharides (LPS) from *Escherichia coli* O55:B5 (Sigma Aldrich – L2637) or 75 µM Deoxycholic acid (DCA) (Sigma Aldrich – D2510-10G) for 24 hours before the Seahorse XF Cell Mito Stress assay (Agilent – 103015-100) was performed using the Seahorse XFe96 Bioanalyzer following manufacturer's instructions. Briefly, oxygen consumption rate (OCR) readings were taken over time under standard conditions and after the sequential addition of mitochondrial inhibitors: 1 µM oligomycin, 0.5 µM FCCP and 0.5 µM rotenone/antimycin. Data were

normalized to signal to blank ratio by Hoechst staining and presented as pmol/min/Norm. Unit.

SA- β-Gal staining

Snap frozen intestinal tissues were embedded in OCT (CellPath – KMA-0100-00A), sectioned at 5 μm in the Cryostat and allowed to dry for at least 30 minutes. Briefly, tissues were fixed for 10 minutes and washed twice with PBS. β-Gal staining solution was applied to each slide and incubated in a humidified chamber at 37°C overnight. The tissues were washed with PBS at 37°C for 10 minutes and a second wash with PBS at RT for a further 5 minutes. Slides were counterstained with eosin and mounted. Twenty-four hours after treatments, CaCo-2 cells were washed once with PBS and stained using the Senescence β-Galactosidase Staining kit (Cell signaling - 9860S) following manufacturer's instructions.

IEC isolation from tissues

Intestinal epithelial cells were isolated from mouse Ileum and colon. Tissue was collected in PBS + 2 % FBS (Sigma – F9665), opened and washed in PBS + 2% FBS containing 0.1 mM DTT and 2 mM EDTA. Solution containing the tissue was incubating twice at 37°C 200 rpm for 10 minutes. After each incubation, solutions containing the cells were vortexed for 15 seconds. The solutions were centrifuged at 1700 rpm for 10 minutes and the pellet was washed in PBS + 2% FBS, pelleted once more and frozen for further analyses.

Intestinal crypt isolation and organoid culture

Small intestinal crypts were isolated from 3 cm segments of ileal tissue from (n=3-5) per group (Control, BDL, DDC or DDC/vanco) as per protocols provided by STEMCELL Technologies (https://www.stemcell.com/intestinal-epithelial-organoid-culture), based on the original culture protocol developed by Sato and colleagues[5]. Briefly, tissues were flushed with ice-cold PBS, opened lengthwise and excess mucus and villi removed by passing with a glass coverslip, then cut into ~2 mm pieces. Crypts were isolated by repeated rinsing and/or gentle trituration of tissue pieces followed by an incubation in enzyme-free dissociation reagent (Gentle Cell Dissociation Reagent, STEMCELL Technologies - 07174) for 15 min at 25 °C and then filtered with a 70 μm strainer. Crypt-

containing fractions were centrifuged at 290 rcf for 5 min at 4°C, and were resuspended in Intesticult[TM] Complete Organoid Growth Medium (STEMCELL Technologies - 06010). Triplicate wells per condition (~400 crypts/well in 50 µl Matrigel® basement membrane [Corning] domes) were plated in a pre-warmed 24-well tissue culture treated plates and incubated 30 min at 37 °C prior to culture in growth medium at 37 °C, 5% CO_2. Resulting organoids were microscopically examined at day 1, 3, and 6 post-plating to assess growth and budding, with culture media changed every 2 days.

Microscopy and measurement of organoids

Tissue culture plates were examined by brightfield microscopy using a 10x CFI Plan ApoChromat lambda D objective (Nikon) on the INCell 6500HS High Content Analyzer (Cytiva) or the Image Xpress Micro4 Imaging System (Molecular Devices) with the standard supplied camera and transmitted light sources. Images obtained from the INCell, were uploaded and converted into OME-TIFF images and analyzed with the OMERO web-client [6, 7]. The best focal plane was determined, and OMERO ROI tools were used to trace and measure the circumference. Images obtained from the Image Xpress were merged into a best focus projection and the circumference of the organoids was traced and measured using FIJI[3]. Measurements were made on randomly chosen organoids from blinded images to prevent observer bias, with 10 (or as many as possible until 10) organoids counted per well. For BDL mice 7days quantification of area of n=10 organoids per n=4 wells per mouse (n=2-3 per condition; control, BDL). For 0.1%DDC fed mice and DDC/vanco, quantification of area of n=10 organoids per n=3 wells per mouse (n=2-4 mice per condition; DDC, DDC/vanco) were done.

Intestinal permeability

Mice were gavaged with FITC-Dextran (44 mg/100g) (Sigma Aldrich – FD4) before sacrificed. FITC was detected in serum samples by fluorescence spectrophotometry (excitation:490nm, emission:530nm). LPS-Binding Protein ELISA (Abcam - AB279407) was performed using serum samples following manufacturer’s instructions.

Bile acid extraction and analysis

A portion of sample (approx. 50 mg) was taken into a screw cap tube along with ceramic beads; 1ml of 90% v/v methanol and 25µl of 40 µg/ml d4-GCA were added and homogenized for 30 seconds at 6000 rpm. The slurry was centrifuged at maximum speed for 10-minutes at 4°C. The supernatant was passed through a hydrophilic-lipophilic balance clean-up cartridge (Waters Oasis Prime HLB, 1cc, 30mg) into capture plate. The cleaned-up extracts in the capture plate were analysed using HPLC – mass spectrometry operated in multiple reaction monitoring (MRM) mode. Each sample (5 µl) was analysed using a Waters Acquity UPLC coupled to a Xevo TQ Absolute triple quadrupole mass spectrometer. HPLC was achieved using a binary gradient of solvent A (Water + 5mM Ammonium Acetate + 0.012% Formic acid) and solvent B (Methanol + 5mM Ammonium Ac + 0.012% Formic acid) at a constant flow rate of 900 µl/min. Separation was made using a Supelco Ascentis Express C18 150 x 4.6, 2.7µm column maintained at 40°C. Injection was made at 50% B and held for 2 min, ramped to 95%B at 20 min and held until 24 minutes. The column equilibrated to initial conditions for 5 minutes. The mass spectrometer was operated in electrospray negative selected ion mode. Quantification was applied using Waters TargetLynx software to integrate detected peak areas relative to the deuterated internal standards.

Bacterial genomic DNA isolation and 16s rRNA sequencing

Bacterial genomic DNA was isolated from faecal pellets using the Fast DNA Soil extraction kit (MPB – 116560200-CF). Genomic DNA was normalized to 5ng/µl with EB (10mM Tris-HCl) and libraries were performed.

Briefly, following a first PCR and clean-up using KAPA Pure Beads (Roche - 07983298001) a second PCR master mix was made up using P7 and P5 of Nextera XT Index Kit v2 index primers (Illumina - FC-131-2001 to 2004). Following the PCR reaction, the libraries were quantified using the Quant-iT dsDNA Assay Kit, high sensitivity kit (Invitrogen - 10164582) and run on a FLUOstar Optima plate reader. Libraries were pooled and run on a High Sensitivity D1000 ScreenTape (Agilent - 5067-5579) using the Agilent Tapestation 4200 to calculate the final library pool molarity. The pool was run on an Illumina MiSeq instrument using MiSeq® Reagent Kit v3 (600 cycle) (Illumina - FC-102-3003) following the Illumina recommended denaturation and loading recommendations which included a

20% PhiX spike in (PhiX Control v3 Illumina - FC-110-3001). The raw data was analysed locally on the MiSeq using MiSeq reporter.

16S sequence analysis

The LotuS2 2.20[8] was used in short amplicon mode with default quality filtering.

Raw 16S rRNA gene reads were quality filtered to ensure a minimum length of 170 bp, not more than eight homonucleotides, no ambiguous bases, average quality >= 27 and an accumulated error below 2.7. For ASVs were clustered in LotuS2 using the DADA2 clustering option. Taxonomy of ASVs was determined using the LotuS2 LCA algorithms against Silva 138.1 reference database[9]. Potential off-target ASVs were removed against the phiX and mouse genome references, as described in[10]. In total 2,107,057/ 3,013,843 passed through various filtering stages and were used for the abundance matrices used for multivariate and univariate testing. Further data analysis was conducted with R statistical language Version 3.00 (The R Foundation, https://www.r-project.org/) as described in Hildebrand et al.[11], employing the rtk software[12] for rarefaction and richness analysis.

Statistical analyses

Statistical analyses were perfomed using GraphPad Prism software version 10.0.3. Statistical differences between two groups were determined by unpaired, two-tailed Student's t-test with Welch's correction. When comparing more than two groups, differences were determined using one-way analysis of variance with Brown-Forsythe and Welch ANOVA tests all performed using GraphPad Prism software settings. Data are shown as mean±SEM. *p<0.05, **p<0.01, ***p<0.001, ****p<0.0001

Supplementary figures

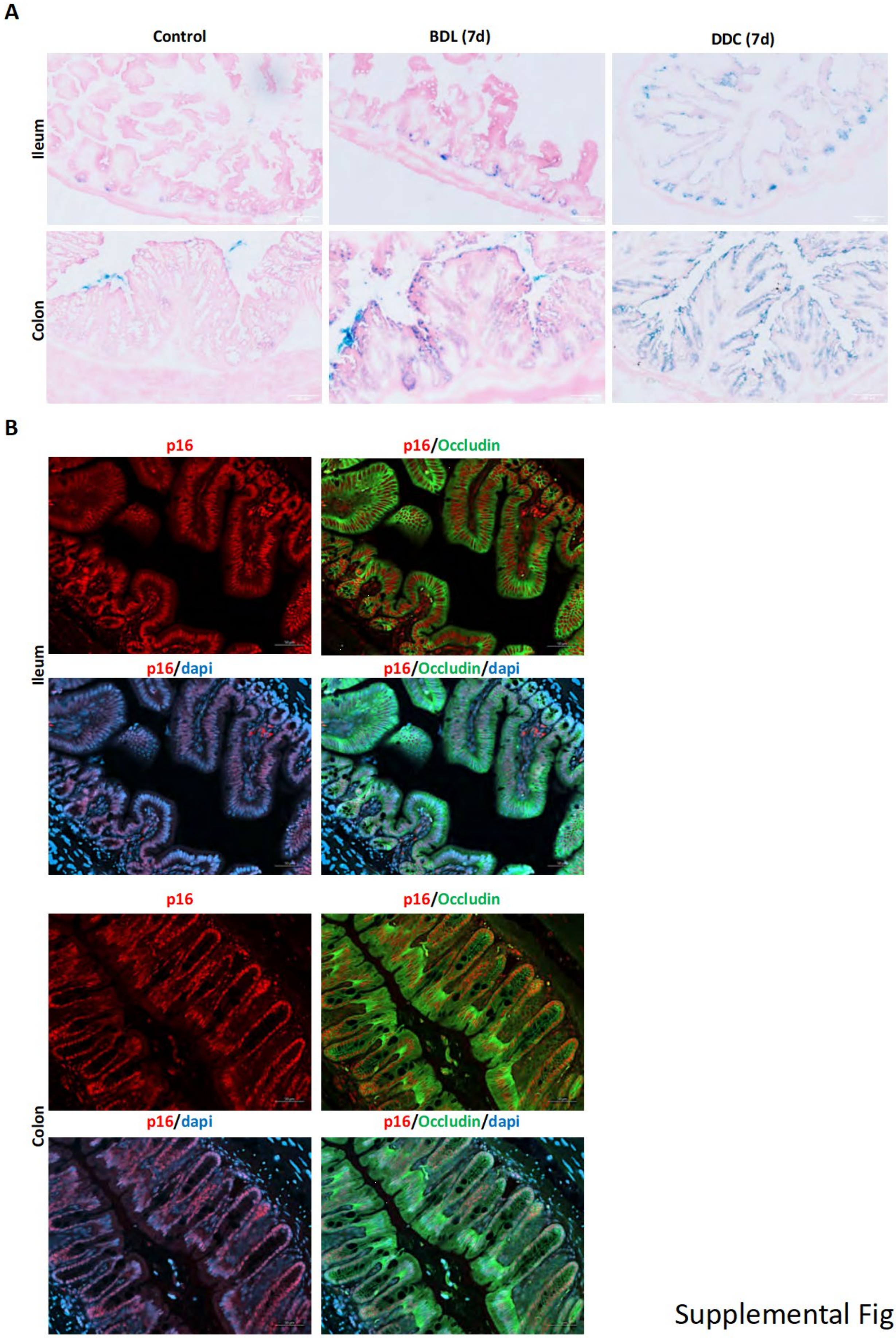

Supplemental Fig 1

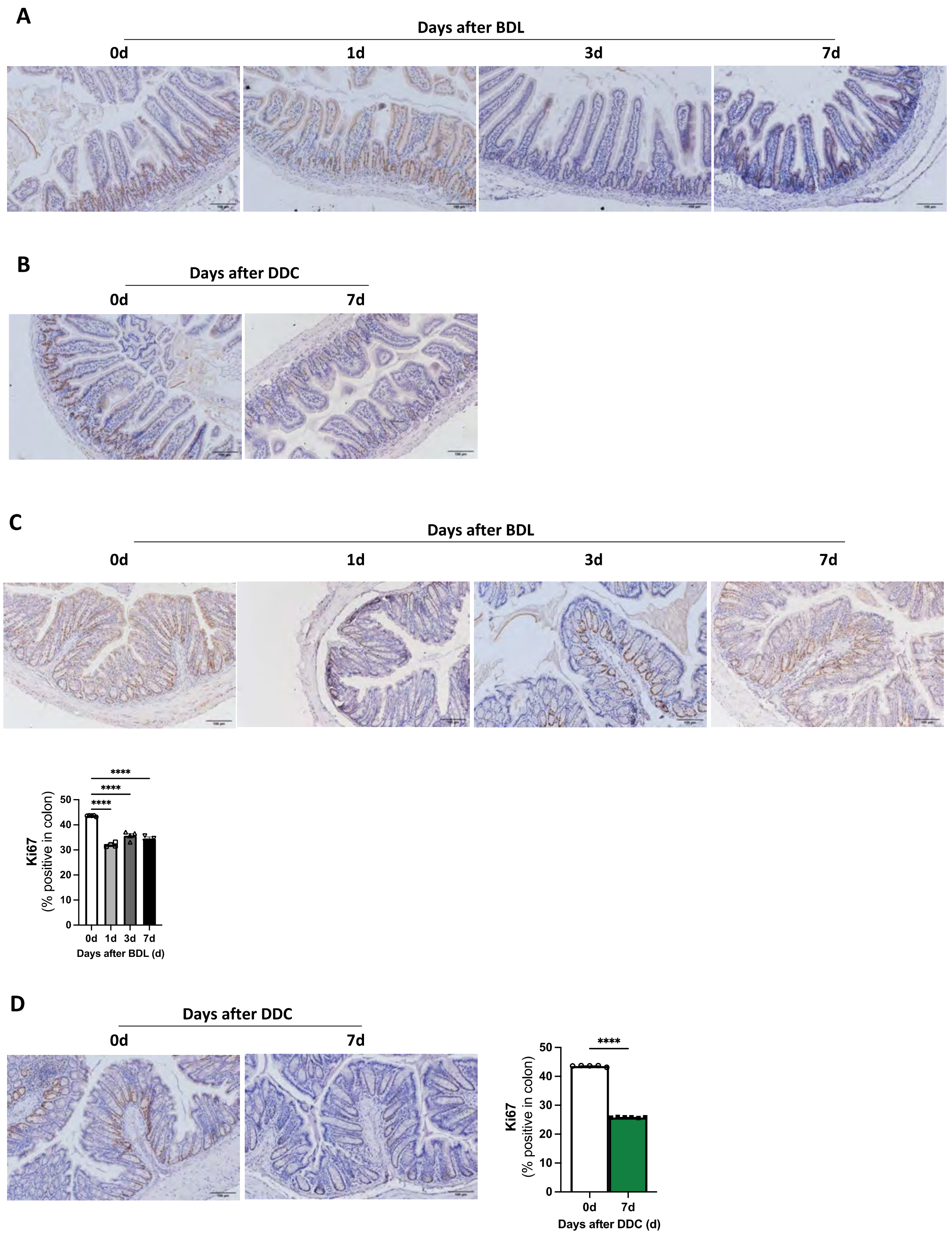

Supplemental Fig 2

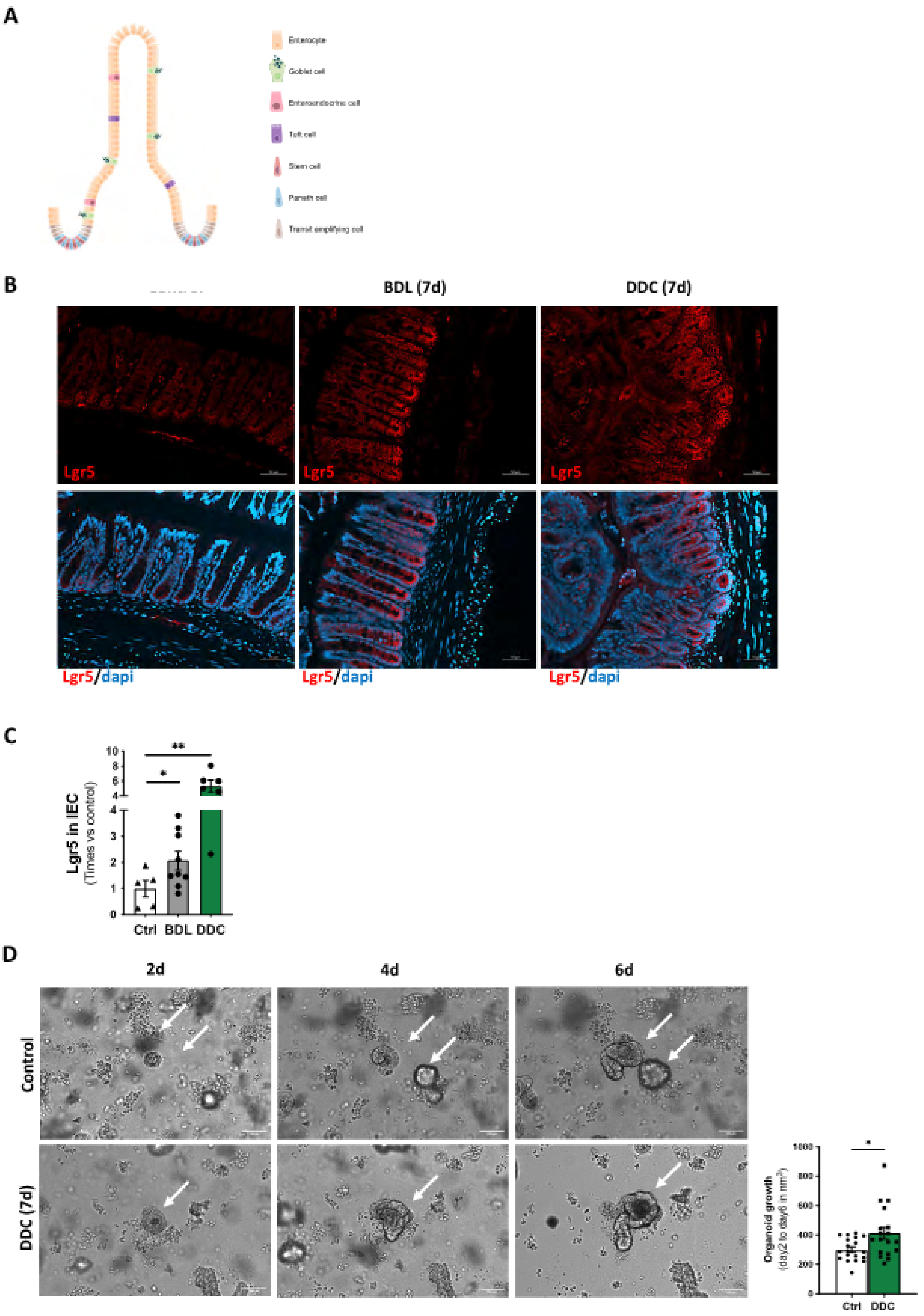

Supplemental Fig 3

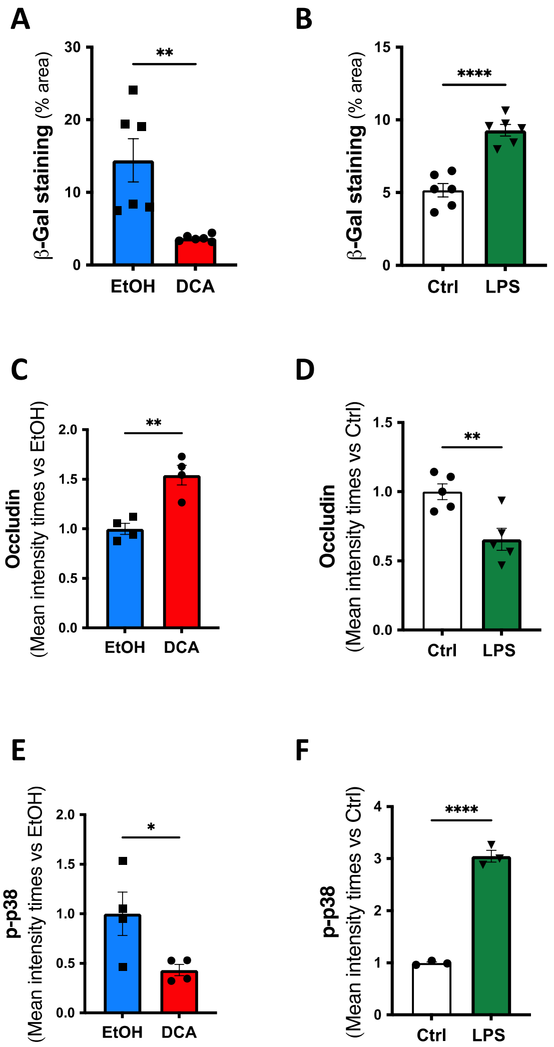

Supplemental Fig 4

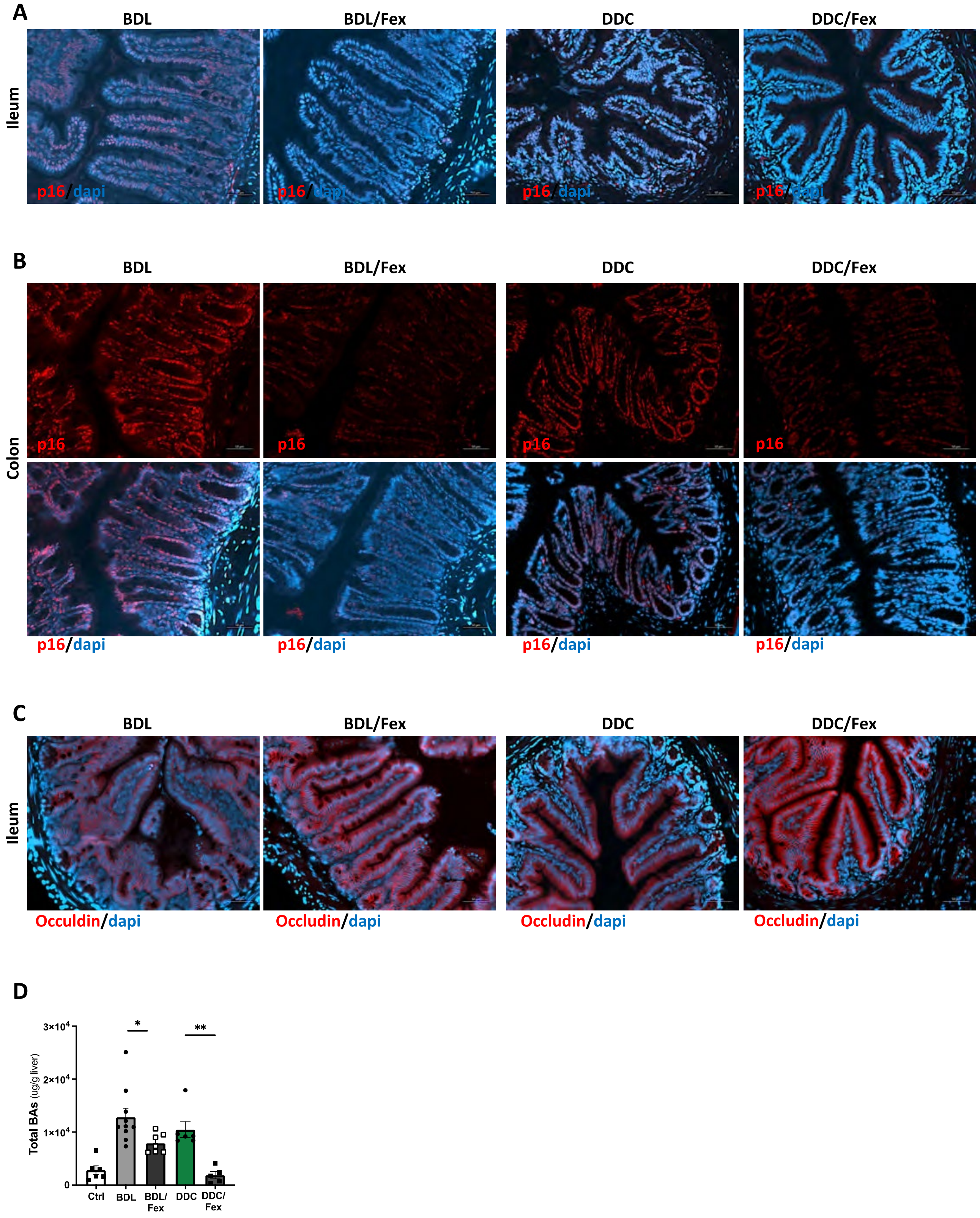

Supplemental Fig 5

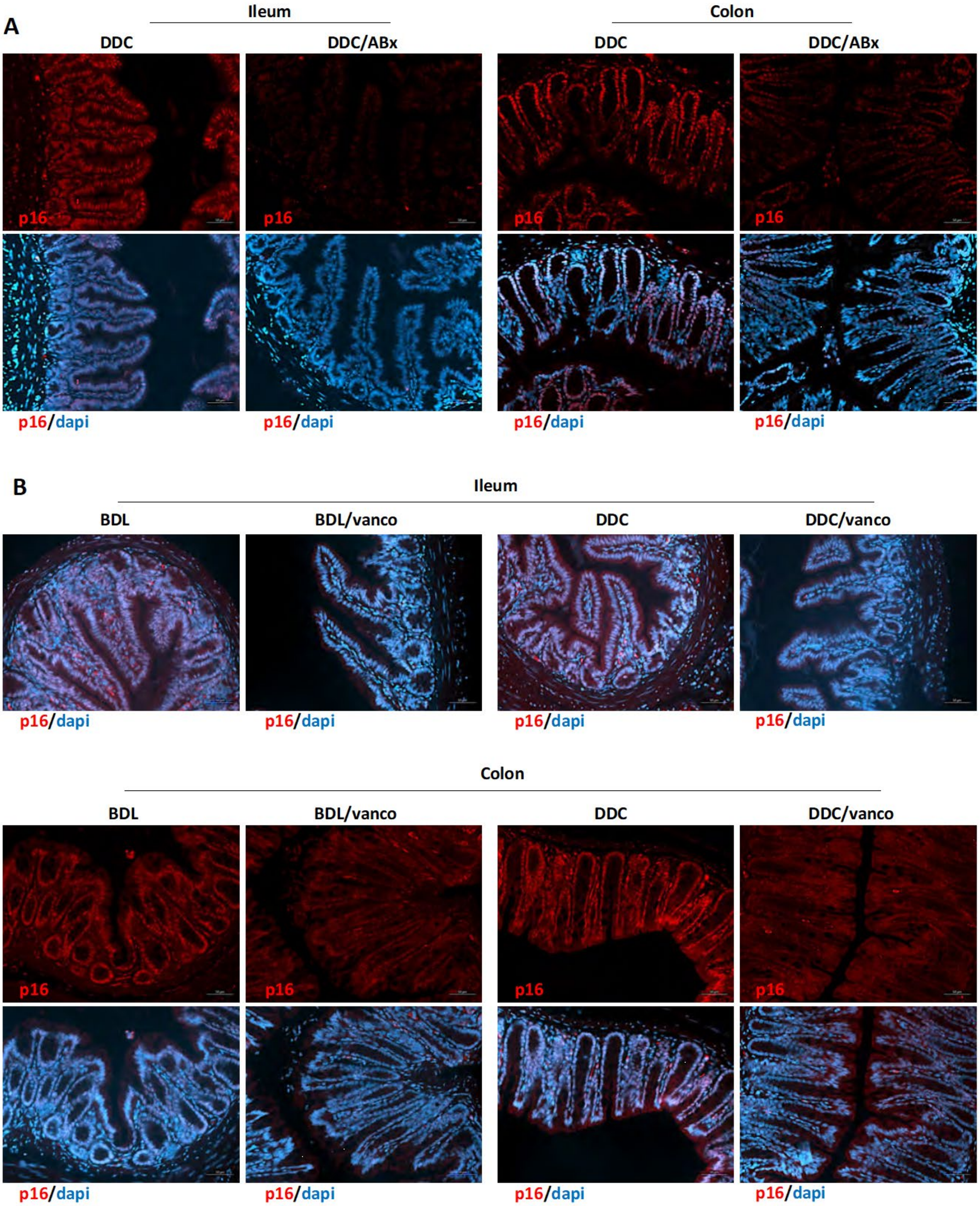

Supplemental Fig 6

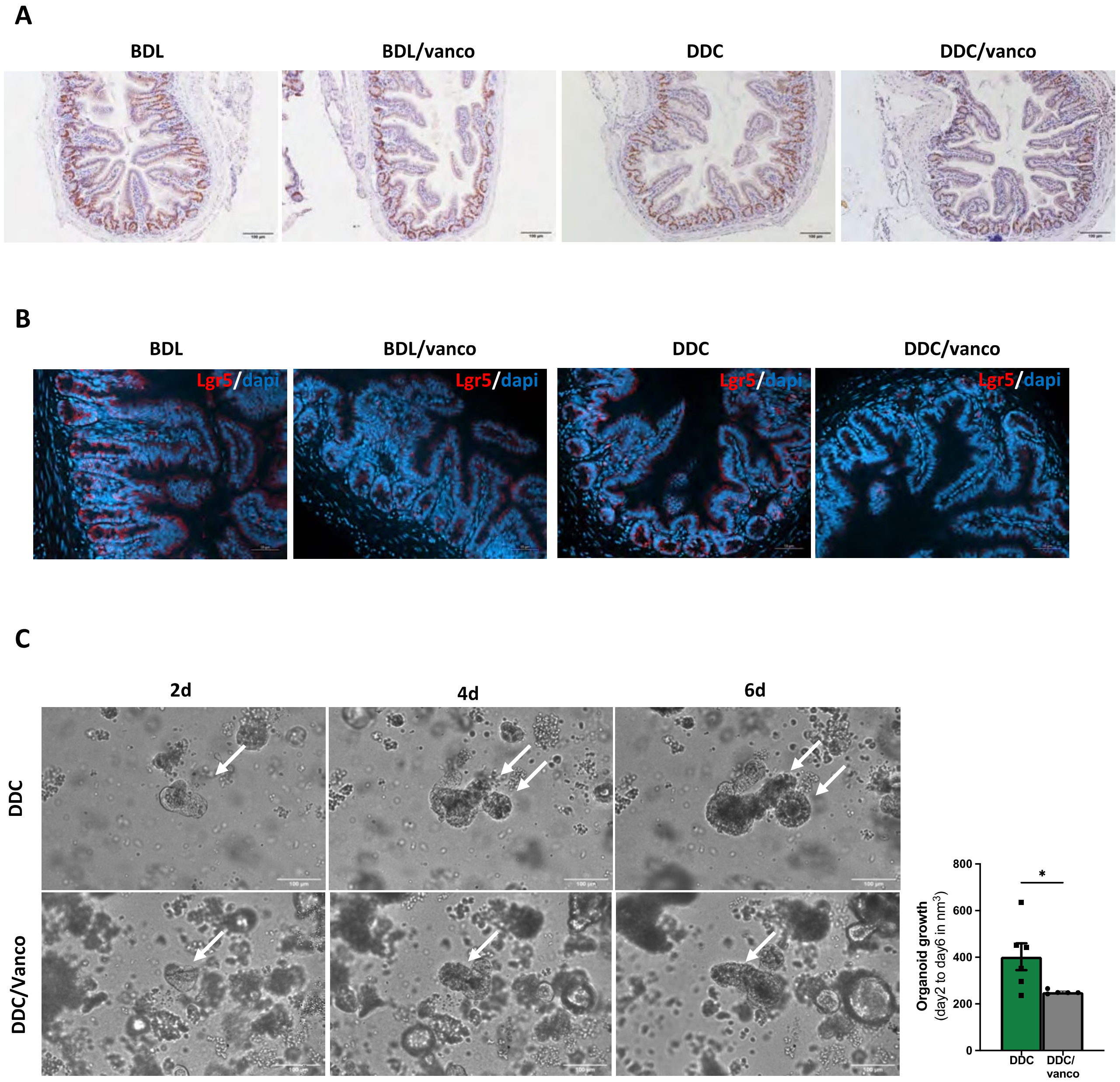

Supplemental Fig 7

A

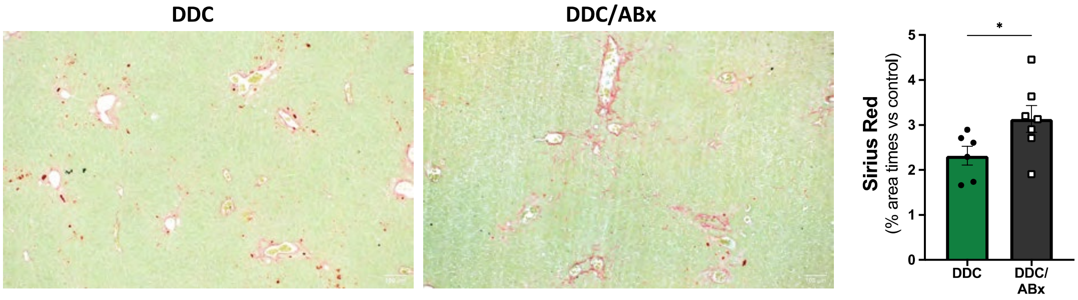

Supplemental Fig 8

A

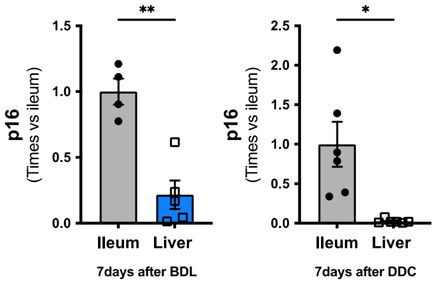

B

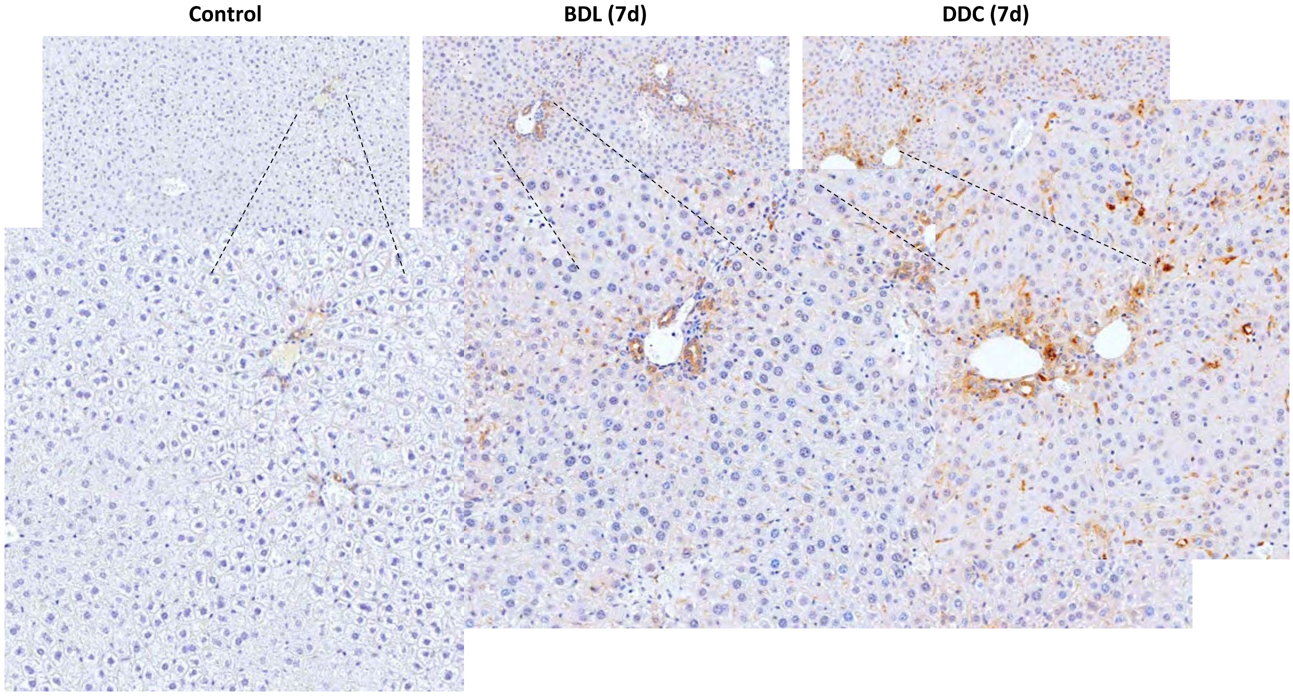

Supplemental Fig 9

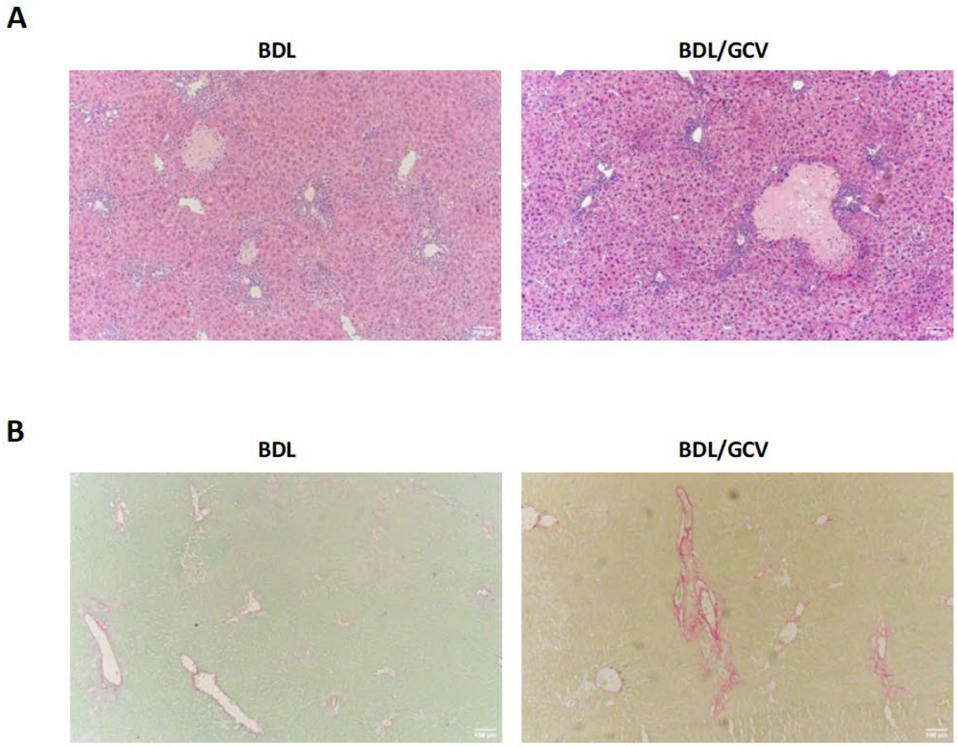

Supplemental Fig 10

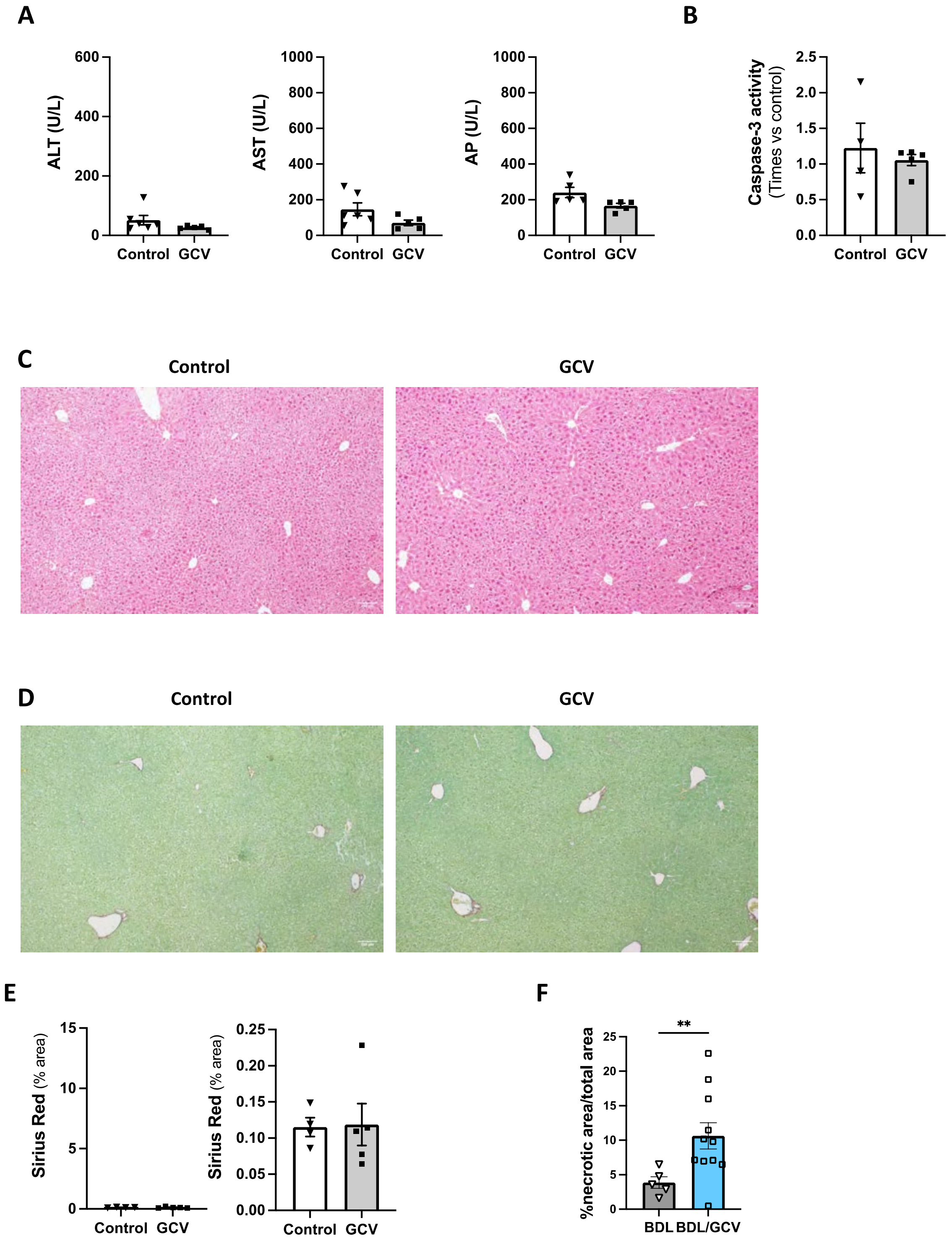

Supplemental Fig 11

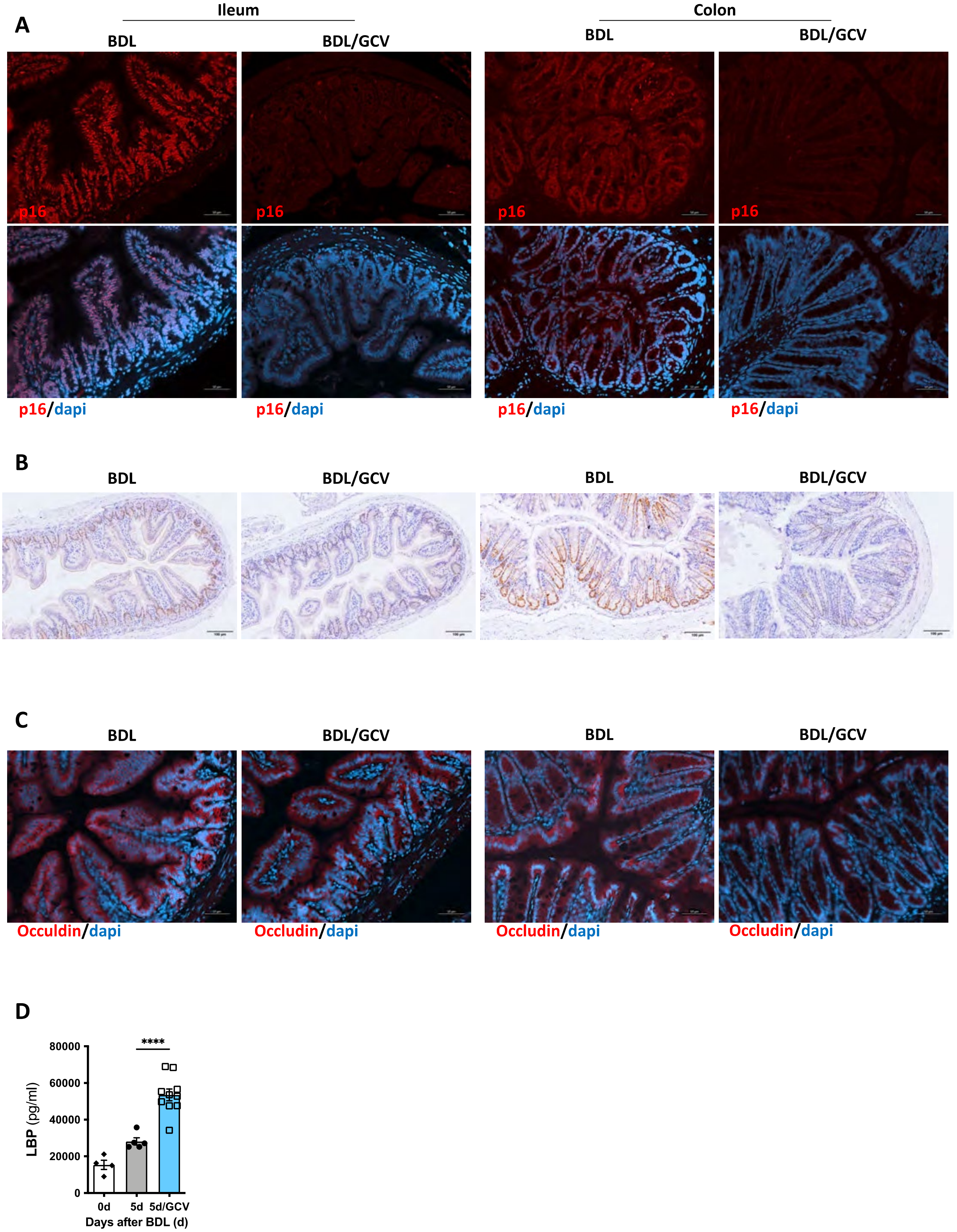

Supplemental Fig 12

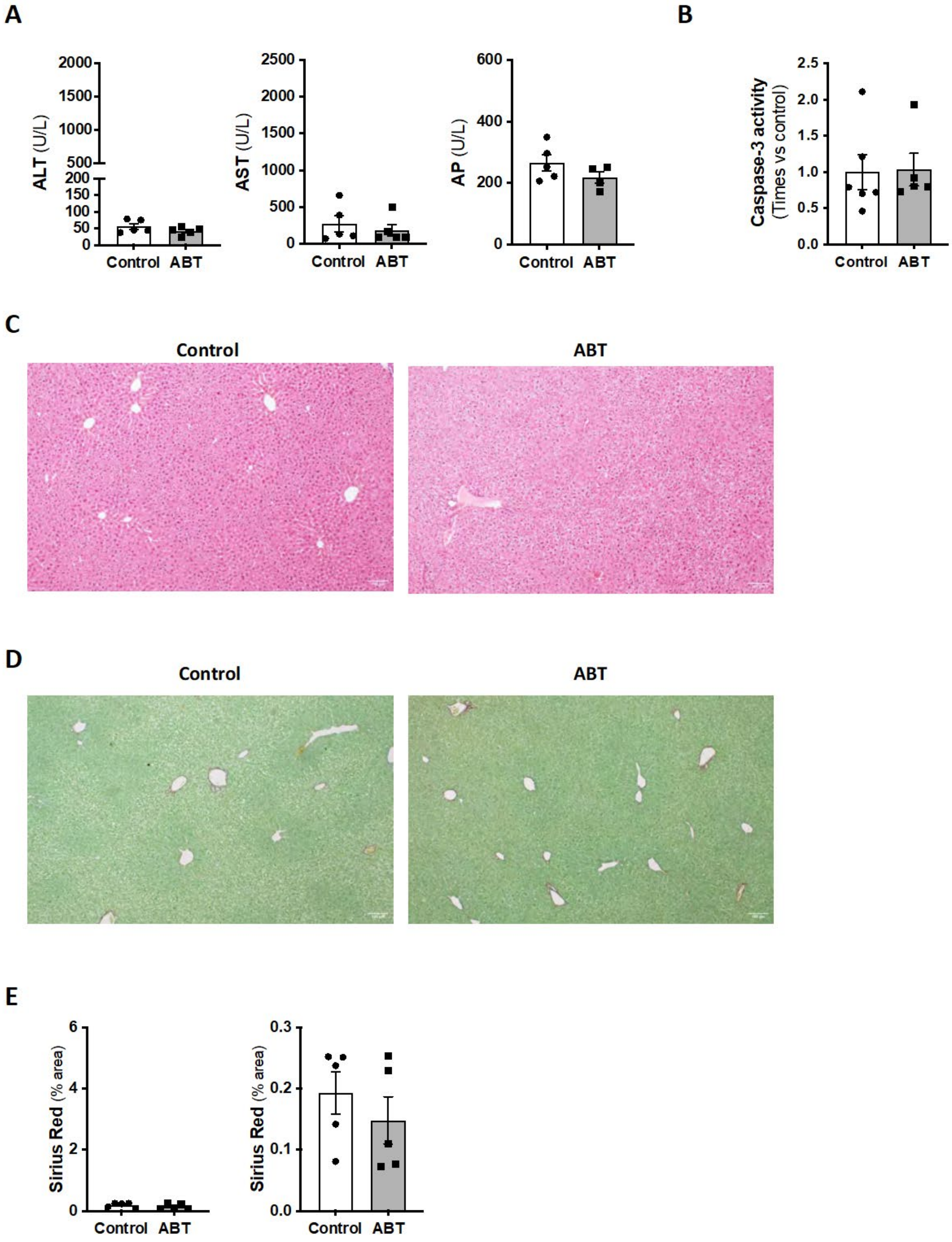

Supplemental Fig 13

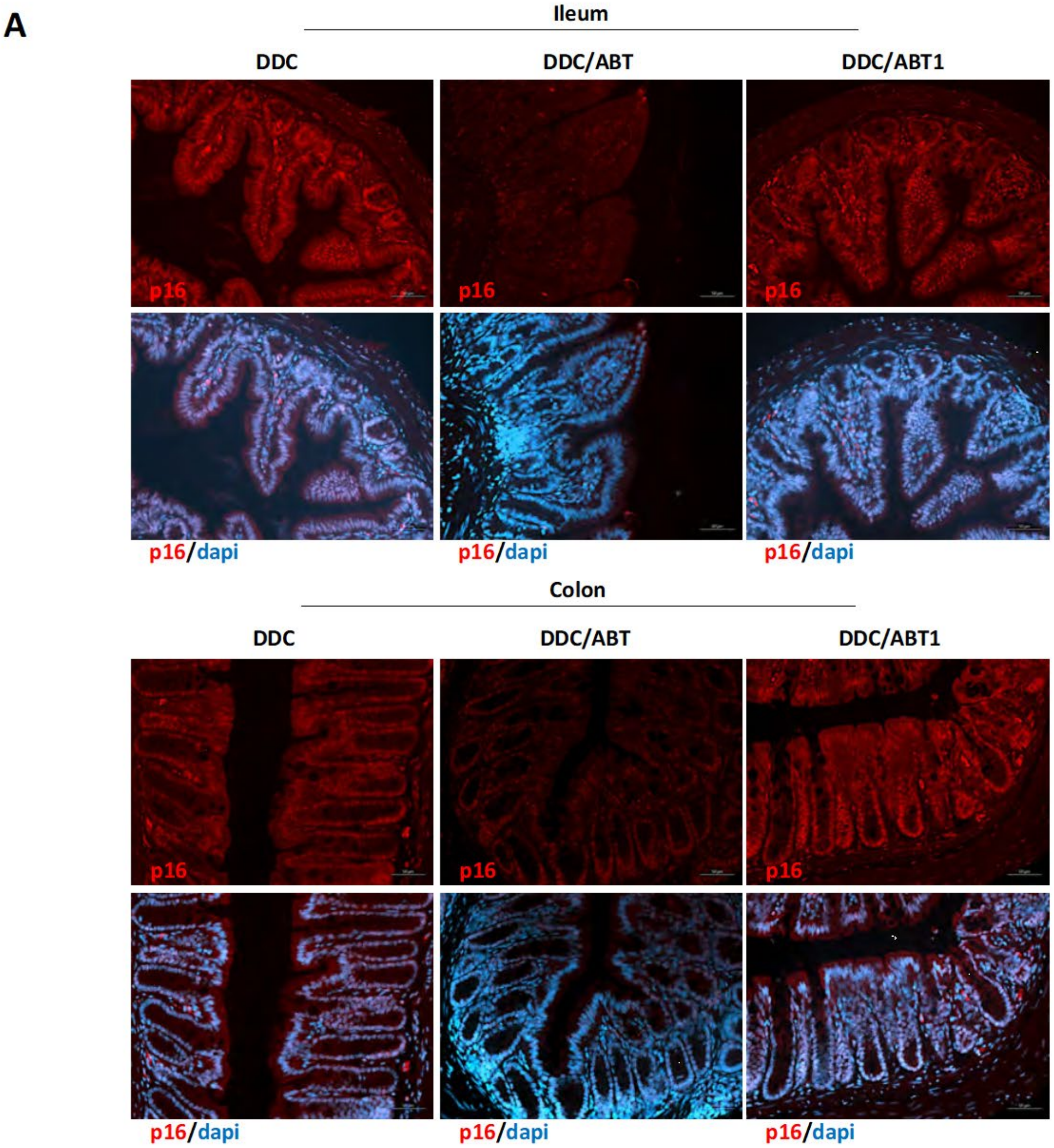

Supplemental Fig 14

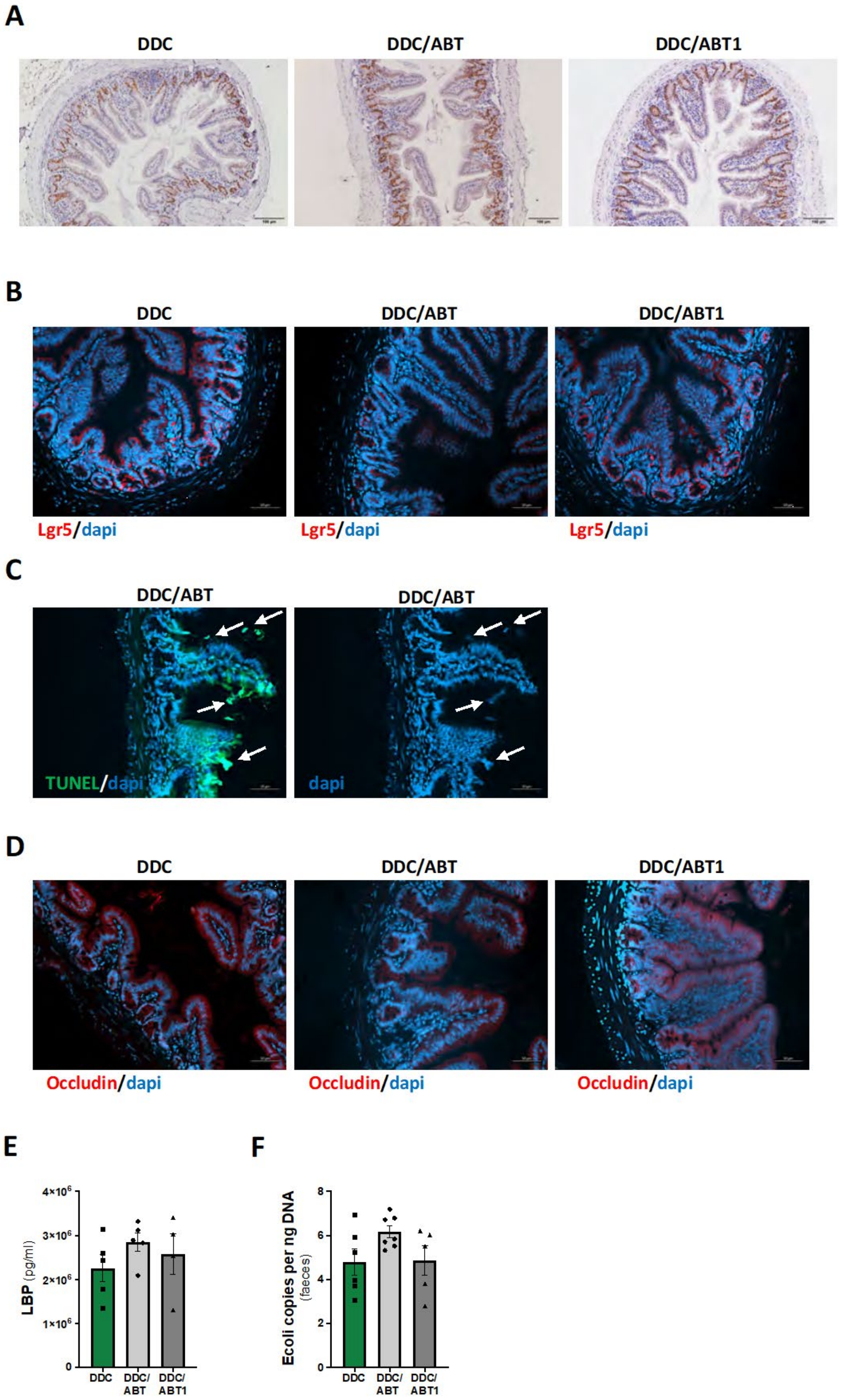

Supplemental Fig 15

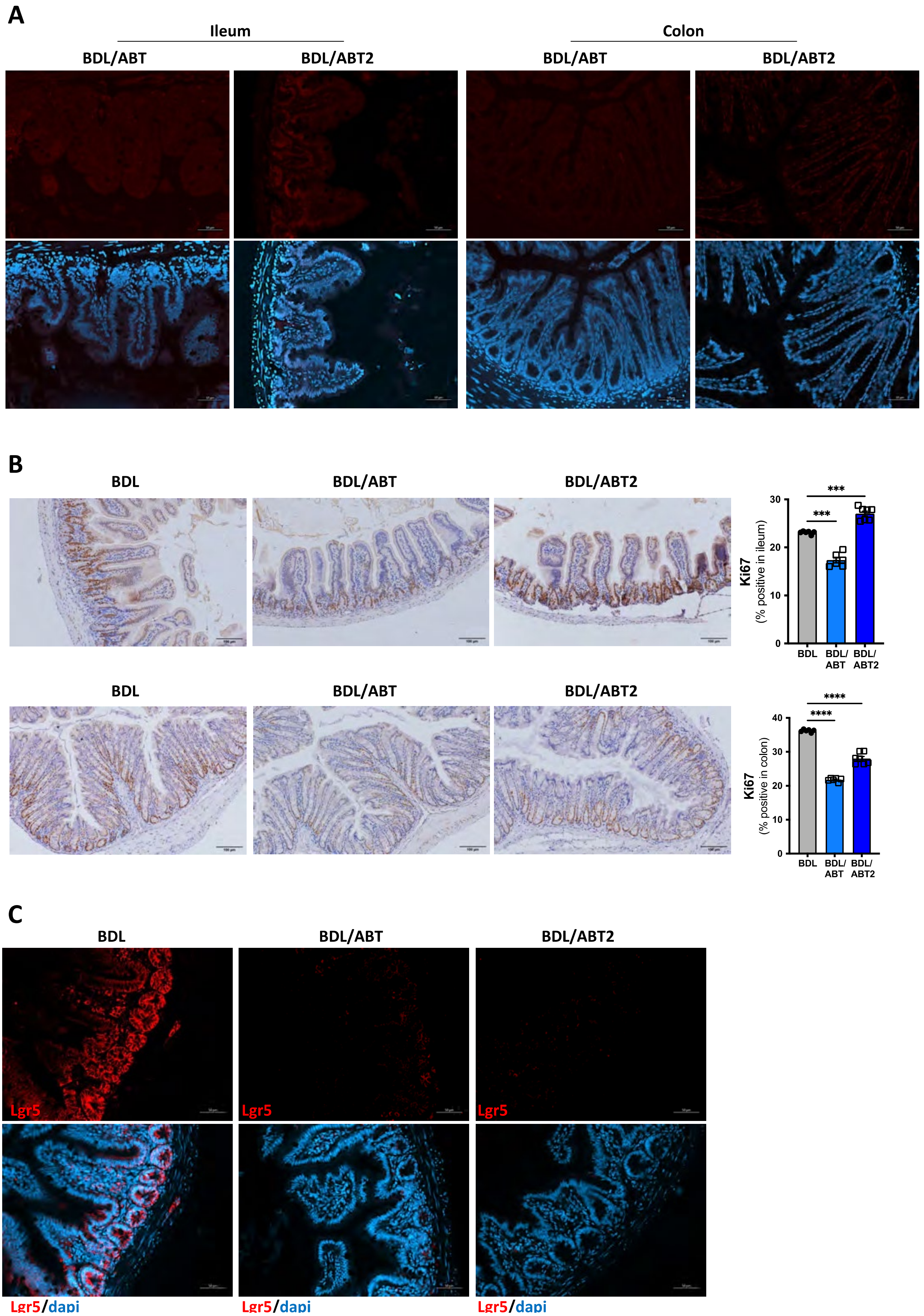

Supplemental Fig 16

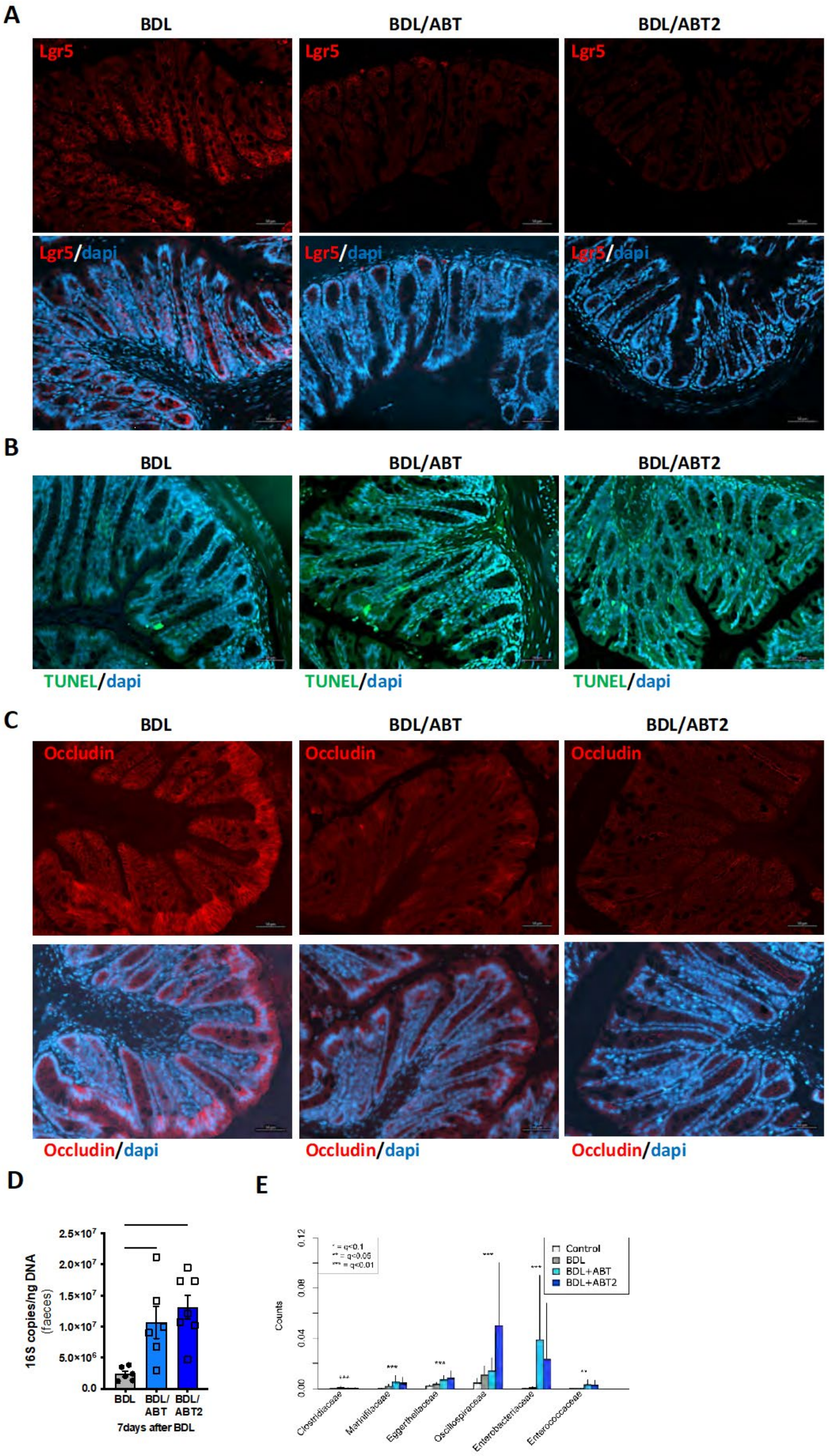

Supplemental Fig 17

Supplementary figure legends

Fig. S1. Senescence is increased in the intestine during murine cholestasis after BDL and DDC diet predominantly in IEC. (A) SA-β-Gal staining in ileum and colon samples (lower panels) from mice 7 days after BDL or 0.1%DDC diet. **(B)** Immunofluorescence in small intestine and colon tissues showing co-localisation of p16 (red) with nuclear dapi staining (blue) in Occludin (green) positive enterocytes. Analyses were done from n=5-6 mice. Representative microscopical images are shown at 20x magnification.

Fig. S2. Reduced proliferation during murine cholestasis after BDL and 0.1%DDC-diet. (A) Immunohistochemistry (IHC) using a Ki67-antibody in small intestine from BDL and **(B)** DDC-diet fed mice as well as IHC and further quantification in **(C)** colon from BDL (****P <0.0001; 0d vs 1d, 0d vs 3d, 0d vs 7d, One-way ANOVA).and **(D)** DDC-diet fed mice (****P <0.0001; control vs DDC, Welch's t-test). Analyses were done from n=5-6 mice. Representative microscopical images are shown at 10x magnification. Values are mean ± SEM.

Fig. S3. Increased ISC stemness in the intestine from mice during cholestasis after BDL and 0.1%DDC diet. (A) Scheme detailing the cellular composition of the small intestinal epithelia **(B)** $Lgr5^+$ immunofluorescence (IF) in colonic sections from mice at 7 days after BDL and DDC diet. Lgr5 in red and DAPI in blue (**C**) qPCR analysis of Lgr5 expression in IEC isolated from the small intestine from BDL and DDC-fed mice (*p=0.0439; control vs BDL, p=0.0016 control vs DDC, Brown-Forsythe and Welch one-way ANOVA). **(D)** *In vitro* growth of organoids from Crypt-cells from the small intestine from control and 0.1%DDC diet-fed mice at day 2, 4 and 6 after initiation of culture (*p=0.0182; control vs DDC, Welch's t-test). Further quantification of area of n=10 organoids per n=3-7 wells per mouse (n=4 per condition; Control, DDC). Analyses were done from n=5-9 mice. Representative microscopical images are shown at 20x magnification (B). For organoid imaging 10x BF optics and a Z stack to get all the focal planes were used (D). Values are mean ± SEM.

Fig. S4. DCA reduced while LPS increased senescence that associates with the modulation of Occludin in intestinal cells *in vitro*. (A) Quantification of SA-b-Gal staining (% per area using

Fiji software) on cultured CaCo-2 cells in response to ethanol and DCA (**p=0.0049 EtOH vs DCA, unpaired t-test) as well as **(B)** in control and LPS-treated cells (****p<0.0001 Ctrl vs LPS, unpaired t-test), **(C)** Quantification of Occludin protein expression in western blots in ethanol and DCA (**p=0.0031 EtOH vs DCA, unpaired t-test) as well as **(D)** in control and LPS-treated cells (**p<0.0079 Ctrl vs LPS, unpaired t-test). **(E)** Quantification of p-p38 protein expression in western blots in ethanol and DCA (*p=0.0459 EtOH vs DCA, unpaired t-test) as well as **(F)** in control and LPS-treated cells (****p<0.0001 Ctrl vs LPS, unpaired t-test). Quantifications were done using ImageLAb software. *In vitro* experiments were repeated 2-3x with n=3-4 replicates. Values are mean ± SEM.

Fig. S5. Pharmacological activation of FXR with Fexaramine reduces intestinal senescence and liver bile acid pool size after BDL and 0.1%DDC diet. (A) p16 IF staining on small intestine and **(B)** colon tissues from BDL/Fexaramine and DDC/Fexaramine fed mice showing reduced senescence. P16 in red and dapi in blue. **(C)** Occludin IF showing restored tight junction protein expression in BDL/Fex and DDC/Fex small intestine samples. Occludin in red and dapi in blue. **(D)** Quantification of the % of necrotic areas in H&E-stained liver sections in mice after BDL (***P* <0.0013; BDL vs BDL/Fex), and **(E)** after DDC diet. (ns p=0.0849; DDC vs DDC/Fex, Welch's t-test). **(F)** Analysis of total bile acids pool size in liver samples using LC-MS (**p*=0.0341; BDL vs BDL/Fex, **p=0.0023 DDC vs DDC/Fex, Brown-Forsythe and Welch one-way ANOVA). Analyses were done from n=6-11 mice. Representative images are shown from 20x magnification. Values are mean ± SEM.

Fig. S6. Antibiotics treatment reduces intestinal senescence in mice after BDL and 0.1%DDC diet. (A) p16 IF staining on small intestine (left panels) and colon tissues (right panels) from DDC fed mice pre-treated with either a cocktail of broad-spectrum antibiotics (Ampicillin (1g/L) in drinking water and Vancomycin (50mg/kg), Neomycin (100mg/kg) and Metronidazole (100mg/kg) by oral gavage 1 day before intervention and throughout the experiment; ABx) or **(B)** Vancomycin only (Vanco; 0.5g/L in drinking water) showing reduced senescence. P16 in red and dapi in blue. Analyses were done from n=6-12 mice. Representative images are shown from 20x magnification.

Fig. S7. Reduced proliferation and ISC stemness in the intestine from vancomycin-treated mice after BDL and 0.1%DDC diet. (A) IHC using a Ki67-antibody in small intestine from BDL/vanco and DDC/vanco mice **(B)** $Lgr5^+$ IF in small intestine sections from BDL/vanco and DDC/vanco mice. Lgr5 in red and DAPI in blue. **(C)** *In vitro* growth of organoids from crypt-cells isolated from the small intestine from DDC and DDC/vanco mice at day 2, 4 and 6 after initiation of culture. Further quantification of area of n=10 organoids per n=3 wells per mouse (n=2-4 mice per condition; DDC, DDC/vanco) (**p*=0.0462; DDC vs DDC/vanco, Welch's t-test). Representative microscopical images are shown at 10x (A, C) and 20x (B) magnification from n=5-9 mice (A, B). Values are mean ± SEM.

Fig. S8. Antibiotics treatment associates with increased liver fibrosis in 0.1%DDC-fed mice. (A) Sirius Red staining and further quantification of % positive area in liver tissue samples from DDC and DDC/ABx mice (with VAMN). (**p*=0.0449; DDC vs DDC/ABx, Welch's t-test). Representative microscopical images are shown at 10x magnification from n=6 mice per group. Values are mean ± SEM. **P* <0.05 (DDC vs DDC/ABx).

Fig. S9. Senescence in the intestine and liver. (A) qPCR analysis of p16 expression in IECs isolated from ileums and livers from BDL and 0.1%DDC diet-fed mice showing increased expression in the intestine vs liver (***p*=0.0011; Ileum vs liver after BDL, **p*=0.0188; Ileum vs liver after DDC, Welch's t-test). **(B)** IHC using a p16 antibody on liver sections showing positive staining in the ductular areas. Representative microscopical images are shown at 20x magnification from n=5-6 mice per group. Values are mean ± SEM. **P* <0.05 and ***P* <0.01 (ileum vs liver in BDL or DDC).

Fig. S10. Elimination of senescent cells exacerbates liver injury and fibrosis after BDL. (A) H&E and **(B)** Sirius Red staining on liver sections from p16-3MR mice at 7 days after BDL and BDL+GCV (25mg/kg) treatment. GCV was given daily i.p. to mice from day 1 after BDL up to the duration of the experiment. Analyses were done from n=4 mice. Representative images are shown from 10x magnification.

Fig. S11. Administration of GCV does not have cytotoxic effects in the liver. (A) Serum transaminases (ALT and AST) and AP levels, (ALT ns p=0.1816, AST ns p=0.0951, AP ns p=0.0614; control vs GCV) **(B)** quantification of Caspase-3 activity in whole liver protein extracts (ns p=0.6636; control vs GCV), **(C)** H&E and **(D)** Sirius Red staining and **(E)** quantification of positively stained area (ns p=0.9231; control vs GCV) on liver sections from p16-3MR mice at 4 days post GCV (25mg/kg) treatment. **(F)** Quantification of % of necrotic areas in H&E-stained liver sections from p16-3MR mice at 5 days after GCV/BDL (**p=0.0061; control vs GCV). Analyses were done from n=5-6 mice/group. Representative images are shown from 10x magnification. Graph scales are matched to GCV/BDL-treatment groups. Values are mean ± SEM. Statistical differences were determined using Welch's t-test.

Fig. S12. Elimination of senescent cells reduced proliferation, tight junction protein expression and increased permeability after BDL. (A) p16 IF in the small intestine and colon from BDL and BDL mice where GCV was given orally, daily from day 1 after BDL up to the duration of the experiment (5 days after surgery; BDL/GCV). p16 in red and dapi in blue. **(B)** Ki67 IHC in small intestine and colon tissues from WT mice at 5 days BDL and BDL/GCV. **(C)** Occludin IF showing reduced tight junction protein expression in BDL/GCV vs BDL in small intestine and colon samples. Occludin in red and dapi in blue. **(D)** Detection of LBP in serum samples from control, BDL and BDL/GCV (****p<0.0001 BDL5d vs BDL5d/GCV, Brown-Forsythe and Welch one-way ANOVA). Analyses were done from n=5-12 mice. Representative images are shown from 20x (A, C) and 10x magnification (B). Values are mean ± SEM. **P* <0.05 (BDL vs BDL/ABT).

Fig. S13. Administration of ABT-263 does not have cytotoxic effects in the liver. (A) Serum transaminases (ALT and AST) and AP levels, (ALT ns p=0.2186, AST ns p=0.5437, AP ns p=0.1824; control vs ABT-263) **(B)** quantification of Caspase-3 activity in whole liver protein extracts, (ns p=0.9169; control vs ABT-263) **(C)** H&E and **(D)** Sirius Red staining and **(E)** quantification of positively stained area (ns p=0.4142; control vs ABT-263) on liver sections from WT mice at 6 days post ABT-263 (50mg/kg) treatment. Analyses were done from n=5 mice/group.

Representative images are shown from 10x magnification. Graph scales are matched to DDC/ABT-263 treatment groups. Values are mean ± SEM. Statistical differences were determined using Welch's t-test.

Fig. S14. Elimination of senescent cells after ABT-293 treatment in 0.1%DDC fed mice. (A) p16 IF in the small intestine (upper panels) and colon (lower panels) from 0.1%DDC diet fed mice treated with ABT-293(50mg/kg) treatment; orally, daily from day 1 after DDC up to the duration of the experiment (DDC/ABT) or daily but only at day 1, 2 and 3 after the initiation of DDC-feeding (DDC/ABT1). Analyses were done from n=5-7 mice. Representative images are shown from 20x magnification.

Fig. S15. Elimination of senescent cells after ABT-293 treatment reduced proliferation, cell death and reduction of tight junction protein expression after 0.1%DDC diet. (A) Ki67 IHC in small intestine tissues from 0.1%DDC-fed mice for 7 days, mice treated with ABT-293 from day1 to the end of the experiment (DDC/ABT) or only at day 1, 2 and 3 of DDC feeding (DDC/ABT1) **(B)** IF showing decreased Lgr5 expression in the small intestine from DDC/ABT mice compared to DDC and DDC/ABT1. Lgr5 in red and dapi in blue. **(C)** TUNEL assay on small intestine tissue sections showing profuse death (in green) and cell shedding (white arrows) in DDC/ABT mice vs DDC and DDC/ABT1. **(D)** Occludin IF showing reduced expression in DDC/ABT mice vs DDC and DDC/ABT1. Occludin in red and dapi in blue. **(E)** Detection of LBP in serum samples (ns p=0.1621 DDC vs DDC/ABT, ns p=0.6158 DDC/ABT vs DDC/ABT1) **(F)** qPCR analysis on faecal samples showing increased *E. coli* presence in DDC/ABT mice vs DDC and DDC/ABT1 while not statistically significant (ns p=0.1470 DDC vs DDC/ABT, ns p=0.9956 DDC/ABT vs DDC/ABT1). Analyses were done from n=4-7 mice. Representative images are shown from 10x (A) and 20x magnification (B, C, D). Statistical differences were determined using Brown-Forsythe and Welch one-way ANOVA.

Fig. S16. Elimination of senescent cells for shorter time and later in the disease reduced colonic IEC proliferation after BDL. (A) p16 IF showing effective elimination of p16 cells after

persistent ABT treatment (from day 1 up to 7d after BDL; BDL/ABT) and later ABT treatment (from day 4 up to day 7 after BDL; BDL/ABT2) p16 in red and dapi in blue. **(B)** Ki67 IHC and further quantification in small intestine (upper panels) (***p=0.0001; BDL vs BDL/ABT, ***p=0.0003; BDL vs BDL/ABT2) and colon tissues (lower panels) (****p<0.0001; BDL vs BDL/ABT; BDL vs BDL/ABT2) from WT mice at 7 days BDL, BDL/ABT and BDL/ABT2 mice. **(C)** Lgr5 IF showing reduced expression in the small intestines from BDL/ABT and BDL/ABT2 compared to BDL mice. Lgr5 in red and dapi in blue. Analyses were done from n=5-7 mice. Representative images are shown from 10x and 20x magnification. Values are mean ± SEM. * Statistical differences were determined using Brown-Forsythe and Welch one-way ANOVA.

Fig. S17. Effects of senolytic drug-mediated elimination of senescent cells in the intestinal barrier and microbiome composition after BDL. (A) Lgr5 IF, **(B)** TUNEL assay and **(C)** Occludin IF in the colon from BDL and BDL/ABT and BDL/ABT2 mice. Lgr5 in red, TUNEL in green, Occludin in red, dapi in blue **(D)** qPCR analysis showing increased 16srRNA expression in faecal samples from BDL/ABT and BDL/ABT2 mice compared to BDL. **(E)** Community composition analysis after 16s rRNA sequencing of faecal samples from BDL, BDL/ABT and BDL/ABT2 mice (*p=0.0462; BDL vs BDL/ABT, **p=0.0021; BDL vs BDL/ABT2) Analyses were done from n=5-7 mice. Representative images are shown from 20x magnification. Values are mean ± SEM. * Statistical differences were determined using Brown-Forsythe and Welch one-way ANOVA.

Supplementary tables

Table S1. PSC patients and control subject clinical data

Patient	Gender	Age	Creatinine (umol/l)	INR	PT (s)	AST (U/L)	ALT (U/L)	AP (U/L)	Bilirubin (umol/l)	Colon Biopsy	Stage of Liver disease (kPA)	Liver Histopathology	Current Treatment
PSC 1	M	71	128	0.87	10.4	41	19	211	8	PSC/UC patchy colitis	8.6	liver Biopsy	Not on Ursodeoxycholic acid
PSC 2	F	47	70	0.92	11.1	30	48	116	13	PSC UC, no active colitis. Normal mucosa	5.7	No liver biopsy undertaken	Ursodeoxycholic acid
PSC 3	M	70	124	0.98	11.8	N/A	8	76	6	PSC colitis quiescent. Normal mucosa	N/A	N/A	Tacrolimus and Azathioprine
PSC 4	M	21	67	1	11.5	39	140	174	7	N/A	5.7	Moderate	Ursodeoxycholic acid
PSC 5	M	21	260	1.02	12.4	28	38	84	16	IBD patchy mild to moderate active colitis	7.2	Mild	Ursodeoxycholic acid
PSC 6	M	41	45	1.09	12.6	98	34	546	19	PSC UC, inflammation, chronic active proctocolitis.	69.9	Severe	Ursodeoxycholic acid
PSC 7	M	25	74	0.91	11	29	22	146	11	PSC UC, colon looks normal, quiescent colitis	5.0	Moderate	Ursodeoxycholic acid and Azathioprine
PSC 8	M	57	98	0.96	11	36	38	81	9	PSC/UC mild inflammation, cryptitis. Quiescent colitis mild.	6.0	No Biopsy available	Not on Ursodeoxycholic acid
PSC 9	M	26	78	0.94	10.8	23	35	72	5	PSC colitis, mild but extensive inflammation, cryptitis, IBD	5.2	Mild	Adalimumab – Not on Ursodeoxycholic acid
PSC 10	M	31	88	0.95	11.5	20	26	175	21	UC PSC looks normal, no inflammation, quiescent UC	5.9	No liver biopsy undertaken	Ursodeoxycholic acid
PSC 11	F	77	88	1.14	13.2	19	22	342	7	PSC, colon polyps, mild inflammation, normal morphology	N/A	None recorded	N/A
PSC 12	M	36	86	1.4	17.2	72	51	256	30	PSC UC colitis, normal histology limits	4.2	No liver biopsy	Not on Ursodeoxycholic acid
PSC 13	F	67	64	N/A	N/A	24	128	55	9	N/A	N/A	No fibrosis	Azathioprine. Not on Ursodeoxycholic acid

Patient	Gender	Age	Creatinine (umol/l)	ALP (U/L)	ALT (U/L)	Bilirubin (umol/l)	Albumin (g/L)	Co-morbidities	Current Treatment
Control 1	M	70	62	103	30	11	40	N/A	Simvastatin (20mg) Clopidogrel (75mg)
Control 2	M	69	74	58	25	8	41	Hay fever, High blood pressure, Osteoarthritis	Valsartan. Simvastatin.
Control 3	M	67	N/A	N/A	N/A	N/A	N/A	High blood pressure, Heart attack, "other heart trouble"	None disclosed
Control 4	M	63	105	66	20	6	43	N/A	Warfarin
Control 5	M	72	78	60	26	14	42	Eczema, Indicated long term illness/disability but did not describe what	Rosuvastatin - certerazine
Control 6	F	61	63	89	19	7	40	Slight nut allergy, Hay fever, Osteoarthritis	Not on medication
Control 7	M	66	64	80	31	12	42	"Other heart trouble"	Atorvastatin, Quinine Sulphate 200mg PRN- very occasional
Control 8	M	73	67	73	29	7	40	N/A	Tamsulosin
Control 9	M	73	65	57	32	5	38	High blood pressure, Asthma, potentially some osteoarthritis	Atorvastatin. Betnovate 0.1% Cream. Carbocisteine. Dermol 500 Lotion. Enalapril. Fenbid Forte. Fexofenadine. Montelukast. Omeprazole. Salbutamol. Trelegy Ellipta.
Control 10	M	71	144	72	24	10	40	High blood pressure	Diclofenac

Table S1. Metadata of PSC patients and control subjects. Serum analyses, disease stage, fibrosis score, medications and diagnosis of colonic biopsied obtained from n=13 PSC patients and n=10 control subjects.

Table S2. Statistical results of enrichment over the taxonomic levels of phylum to species.

Phylum

Rank	Feature name	p-value	q-value	Direction
1	Bacteria;Deferribacterota	3.71E-05	0.000407881	Control>>>BDL > DDC
2	Bacteria;Verrucomicrobiota	0.000872386	0.003486239	Control > BDL = DDC
3	Bacteria;Proteobacteria	0.000950793	0.003486239	DDC > BDL > Control
4	Bacteria;Cyanobacteria	0.002807317	0.006182997	Control>> BDL = DDC
5	Bacteria;Patescibacteria	0.002810453	0.006182997	Control = BDL > DDC
6	Bacteria;Desulfobacterota	0.005547956	0.010171252	Control>= DDC>= BDL
7	Bacteria;Bacteroidota	0.02696733	0.042377232	BDL = DDC = Control
8	Bacteria;Firmicutes	0.105224205	0.144683282	Control = BDL = DDC
9	noHit;	0.139370117	0.170341255	Control = BDL = DDC
10	Bacteria;Actinobacteriota	0.339028395	0.372931234	NA
11	Bacteria;?	0.454435652	0.454435652	NA

Class

Rank	Feature name	p-value	q-value	Direction
1	Bacteria;Deferribacterota;Deferribacteres	3.71E-05	0.00063	Control>>>BDL > DDC
2	Bacteria;Actinobacteriota;Actinobacteria	0.000343	0.002919	BDL > DDC > Control
3	Bacteria;Verrucomicrobiota;Verrucomicrobiae	0.000872	0.004944	Control > BDL = DDC
4	Bacteria;Firmicutes;?	0.001789	0.006712	Control>> DDC = BDL
5	Bacteria;Cyanobacteria;Vampirivibrionia	0.001974	0.006712	Control>> BDL = DDC
6	Bacteria;Patescibacteria;Saccharimonadia	0.00281	0.007846	Control = BDL > DDC
7	Bacteria;Proteobacteria;Gammaproteobacteria	0.003231	0.007846	DDC>= BDL>= Control
8	Bacteria;Desulfobacterota;Desulfovibrionia	0.005548	0.011789	Control>= DDC>= BDL
9	Bacteria;Proteobacteria;Alphaproteobacteria	0.02222	0.041972	DDC = BDL>= Control
10	Bacteria;Bacteroidota;Bacteroidia	0.026967	0.045844	BDL = DDC = Control
11	Bacteria;Firmicutes;Bacilli	0.038162	0.058978	Control > DDC = BDL
12	Bacteria;Actinobacteriota;?	0.124634	0.176565	Control = DDC = BDL
13	noHit;	0.13937	0.177877	Control = BDL = DDC
14	Bacteria;Firmicutes;Clostridia	0.146487	0.177877	DDC = BDL = Control
15	Bacteria;Cyanobacteria;Cyanobacteriia	0.275046	0.311719	NA
16	Bacteria;Actinobacteriota;Coriobacteriia	0.361519	0.384113	NA
17	Bacteria;?;?	0.454436	0.454436	NA

Order

Rank	Feature name	p-value	q-value	Direction
1	Bacteria;Deferribacterota;Deferribacteres;Deferribacterales	3.71E-05	0.001446	Control>>>BDL > DDC
2	Bacteria;Bacteroidota;Bacteroidia;?	0.000114	0.001524	Control>> BDL = DDC
3	Bacteria;Actinobacteriota;Actinobacteria;Corynebacteriales	0.000117	0.001524	BDL>> DDC>> Control
4	Bacteria;Proteobacteria;Gammaproteobacteria;Enterobacterales	0.000188	0.001828	DDC = BDL>>>Control
5	Bacteria;Firmicutes;Bacilli;Erysipelotrichales	0.000764	0.005671	Control>> DDC = BDL
6	Bacteria;Verrucomicrobiota;Verrucomicrobiae;Verrucomicrobiales	0.000872	0.005671	Control > BDL = DDC
7	Bacteria;Firmicutes;?;?	0.001789	0.009624	Control>> DDC = BDL

8	Bacteria;Cyanobacteria;Vampirivibrionia;Gastranaerophilales	0.001974	0.009624	Control>> BDL = DDC
9	Bacteria;Patescibacteria;Saccharimonadia;Saccharimonadales	0.00281	0.012119	Control = BDL > DDC
10	Bacteria;Actinobacteriota;Actinobacteria;Micrococcales	0.003107	0.012119	DDC = Control = BDL
11	Bacteria;Desulfobacterota;Desulfovibrionia;Desulfovibrionales	0.005548	0.01967	Control>= DDC>= BDL
12	Bacteria;Firmicutes;Clostridia;Clostridiales	0.014183	0.046094	Control > BDL > DDC
13	Bacteria;Proteobacteria;Alphaproteobacteria;Rhodospirillales	0.02222	0.063972	DDC = BDL>= Control
14	Bacteria;Firmicutes;Bacilli;Lactobacillales	0.024192	0.063972	BDL = DDC = Control
15	Bacteria;Bacteroidota;Bacteroidia;Bacteroidales	0.026967	0.063972	BDL = DDC = Control
16	Bacteria;Firmicutes;Clostridia;?	0.027019	0.063972	BDL = DDC>>>Control
17	Bacteria;Firmicutes;Clostridia;Clostridia vadinBB60 group	0.027885	0.063972	Control > DDC = BDL
18	Bacteria;Firmicutes;Clostridia;Oscillospirales	0.032331	0.07005	Control>>>DDC = BDL
19	Bacteria;Firmicutes;Bacilli;Acholeplasmatales	0.042517	0.078961	Control = BDL = DDC
20	Bacteria;Firmicutes;Bacilli;RF39	0.042517	0.078961	Control = BDL = DDC
21	Bacteria;Proteobacteria;Gammaproteobacteria;Pseudomonadales	0.042517	0.078961	Control = BDL = DDC
22	Bacteria;Firmicutes;Clostridia;Peptococcales	0.064436	0.114228	Control = BDL = DDC
23	Bacteria;Firmicutes;Clostridia;Christensenellales	0.079493	0.134792	Control = BDL = DDC
24	Bacteria;Actinobacteriota;Actinobacteria;Propionibacteriales	0.096328	0.156532	Control = BDL = DDC
25	Bacteria;Firmicutes;Clostridia;Clostridia UCG-014	0.105936	0.165261	Control = BDL = DDC
26	Bacteria;Actinobacteriota;?;?	0.124634	0.186951	Control = DDC = BDL
27	noHit;	0.13937	0.195326	Control = BDL = DDC
28	Bacteria;Actinobacteriota;Actinobacteria;Pseudonocardiales	0.140234	0.195326	Control = BDL = DDC
29	Bacteria;Firmicutes;Clostridia;Monoglobales	0.152784	0.205468	Control = BDL = DDC
30	Bacteria;Proteobacteria;Alphaproteobacteria;Rickettsiales	0.168378	0.218892	DDC = Control = BDL
31	Bacteria;Firmicutes;Clostridia;Peptostreptococcales-Tissierellales	0.197726	0.248752	Control = BDL = DDC
32	Bacteria;Firmicutes;Bacilli;Staphylococcales	0.235989	0.287612	NA
33	Bacteria;Cyanobacteria;Cyanobacteriia;Chloroplast	0.275046	0.325055	NA
34	Bacteria;Firmicutes;Clostridia;Lachnospirales	0.322195	0.369577	NA
35	Bacteria;Actinobacteriota;Coriobacteriia;Coriobacteriales	0.361519	0.402835	NA
36	Bacteria;Proteobacteria;Alphaproteobacteria;Rhizobiales	0.388896	0.421304	NA
37	Bacteria;Proteobacteria;Gammaproteobacteria;Burkholderiales	0.429062	0.452255	NA
38	Bacteria;?;?;?	0.454436	0.466394	NA
39	Bacteria;Firmicutes;Bacilli;?	0.853157	0.853157	NA

Family

Rank	Feature name	p-value	q-value	Direction
1	Bacteria;Proteobacteria;Gammaproteobacteria;Burkholderiales;?	1.44E-05	0.000821	Control>>>BDL = DDC
2	Bacteria;Firmicutes;Bacilli;Erysipelotrichales;Erysipelatoclostridiaceae	2.97E-05	0.000821	Control>>>BDL = DDC

3	Bacteria;Deferribacterota;Deferribacteres;Deferribacterales;Deferribacteraceae	3.71E-05	0.000821	Control>>>BDL > DDC
4	Bacteria;Firmicutes;Clostridia;Clostridia vadinBB60 group;uncultured Clostridiales bacterium	4.38E-05	0.000821	Control>>>BDL = DDC
5	Bacteria;Firmicutes;Bacilli;Lactobacillales;Enterococcaceae	6.21E-05	0.000932	DDC>> BDL>>>Control
6	Bacteria;Bacteroidota;Bacteroidia;?;?	0.000114	0.001256	Control>> BDL = DDC
7	Bacteria;Actinobacteriota;Actinobacteria;Corynebacteriales;Corynebacteriaceae	0.000117	0.001256	BDL>> DDC>> Control
8	Bacteria;Proteobacteria;Gammaproteobacteria;Enterobacterales;Enterobacteriaceae	0.000188	0.001758	DDC = BDL>>>Control
9	Bacteria;Firmicutes;Bacilli;Erysipelotrichales;Erysipelotrichaceae	0.000764	0.006368	Control>> DDC = BDL
10	Bacteria;Verrucomicrobiota;Verrucomicrobiae;Verrucomicrobiales;Akkermansiaceae	0.000947	0.006682	Control > BDL = DDC
11	Bacteria;Actinobacteriota;Actinobacteria;Micrococcales;Microbacteriaceae	0.00098	0.006682	DDC>> Control = BDL
12	Bacteria;Firmicutes;Clostridia;Lachnospirales;?	0.00173	0.009871	Control>>>DDC = BDL
13	Bacteria;Firmicutes;?;?;?	0.001789	0.009871	Control>> DDC = BDL
14	Bacteria;Bacteroidota;Bacteroidia;Bacteroidales;Tannerellaceae	0.001969	0.009871	DDC > BDL = Control
15	Bacteria;Cyanobacteria;Vampirivibrionia;Gastranaerophilales;?	0.001974	0.009871	Control>> BDL = DDC
16	Bacteria;Patescibacteria;Saccharimonadia;Saccharimonadales;Saccharimonadaceae	0.00281	0.013174	Control = BDL > DDC
17	Bacteria;Firmicutes;Clostridia;Peptostreptococcales-Tissierellales;Anaerovoracaceae	0.00399	0.017601	Control = BDL>= DDC
18	Bacteria;Bacteroidota;Bacteroidia;Bacteroidales;Muribaculaceae	0.004265	0.017772	BDL = Control > DDC
19	Bacteria;Desulfobacterota;Desulfovibrionia;Desulfovibrionales;Desulfovibrionaceae	0.005548	0.0219	Control>= DDC>= BDL
20	Bacteria;Firmicutes;Clostridia;Clostridia vadinBB60 group;uncultured organism	0.006683	0.025062	Control > BDL = DDC
21	Bacteria;Firmicutes;Clostridia;Oscillospirales;Ruminococcaceae	0.007293	0.026047	Control = BDL = DDC
22	Bacteria;Firmicutes;Clostridia;Clostridiales;?	0.014183	0.042548	Control > BDL > DDC
23	Bacteria;Firmicutes;Clostridia;Clostridiales;Clostridiaceae	0.014183	0.042548	Control > BDL > DDC
24	Bacteria;Firmicutes;Clostridia;Oscillospirales;UCG-010	0.014183	0.042548	Control > BDL > DDC
25	Bacteria;Proteobacteria;Gammaproteobacteria;Pseudomonadales;Pseudomonadaceae	0.014183	0.042548	Control > BDL > DDC
26	Bacteria;Firmicutes;Bacilli;Lactobacillales;Streptococcaceae	0.021264	0.060271	BDL>= Control = DDC
27	Bacteria;Proteobacteria;Alphaproteobacteria;Rhodospirillales;?	0.02222	0.060271	DDC = BDL>= Control
28	Bacteria;Bacteroidota;Bacteroidia;Bacteroidales;?	0.022501	0.060271	Control = BDL = DDC
29	Bacteria;Firmicutes;Clostridia;Oscillospirales;?	0.025018	0.064702	Control = BDL = DDC
30	Bacteria;Proteobacteria;Gammaproteobacteria;Burkholderiales;Burkholderiaceae	0.025934	0.064836	Control>= BDL = DDC
31	Bacteria;Firmicutes;Clostridia;?;?	0.027019	0.065368	BDL = DDC>>>Control
32	Bacteria;Firmicutes;Bacilli;Lactobacillales;Lactobacillaceae	0.030054	0.07044	BDL = DDC = Control
33	Bacteria;Firmicutes;Clostridia;Clostridia vadinBB60 group;?	0.032135	0.073035	Control > DDC = BDL
34	Bacteria;Firmicutes;Bacilli;Acholeplasmatales;Acholeplasmataceae	0.042517	0.086553	Control = BDL = DDC

35	Bacteria;Firmicutes;Clostridia;Oscillospirales;Butyricicoccaceae	0.042517	0.086553	Control = BDL = DDC
36	Bacteria;Bacteroidota;Bacteroidia;Bacteroidales;Bacteroidaceae	0.043192	0.086553	DDC = BDL > Control
37	Bacteria;Actinobacteriota;Coriobacteriia;Coriobacteriales;Eggerthellaceae	0.043233	0.086553	Control = BDL = DDC
38	Bacteria;Firmicutes;Bacilli;Lactobacillales;?	0.043854	0.086553	Control > DDC = BDL
39	Bacteria;Firmicutes;Bacilli;RF39;uncultured rumen bacterium	0.046605	0.087385	Control>= BDL>= DDC
40	Bacteria;Firmicutes;Clostridia;Christensenellales;?	0.046605	0.087385	Control>= BDL>= DDC
41	Bacteria;Verrucomicrobiota;Verrucomicrobiae;Verrucomicrobiales;?	0.06092	0.111439	DDC = BDL = Control
42	Bacteria;Firmicutes;Clostridia;Peptococcales;Peptococcaceae	0.064436	0.115065	Control = BDL = DDC
43	Bacteria;Firmicutes;Clostridia;Oscillospirales;Oscillospiraceae	0.066303	0.115646	Control>> DDC = BDL
44	Bacteria;Firmicutes;Clostridia;Christensenellales;Christensenellaceae	0.079493	0.135499	Control = BDL = DDC
45	Bacteria;Actinobacteriota;Actinobacteria;Propionibacteriales;Propionibacteriaceae	0.096328	0.153714	Control = BDL = DDC
46	Bacteria;Firmicutes;Bacilli;Lactobacillales;Aerococcaceae	0.096328	0.153714	Control = BDL = DDC
47	Bacteria;Proteobacteria;Gammaproteobacteria;Burkholderiales;Comamonadaceae	0.096328	0.153714	Control = BDL = DDC
48	Bacteria;Firmicutes;Clostridia;Clostridia UCG-014;?	0.105936	0.165526	Control = BDL = DDC
49	Bacteria;Proteobacteria;Gammaproteobacteria;Enterobacterales;?	0.114864	0.175812	DDC = Control = BDL
50	Bacteria;Actinobacteriota;Coriobacteriia;Coriobacteriales;Atopobiaceae	0.1206	0.1809	Control > BDL = DDC
51	Bacteria;Actinobacteriota;?;?;?	0.124634	0.183286	Control = DDC = BDL
52	Bacteria;Bacteroidota;Bacteroidia;Bacteroidales;Prevotellaceae	0.137587	0.194769	Control>= BDL = DDC
53	noHit;	0.13937	0.194769	Control = BDL = DDC
54	Bacteria;Actinobacteriota;Actinobacteria;Pseudonocardiales;Pseudonocardiaceae	0.140234	0.194769	Control = BDL = DDC
55	Bacteria;Firmicutes;Clostridia;Monoglobales;Monoglobaceae	0.152784	0.208342	Control = BDL = DDC
56	Bacteria;Proteobacteria;Alphaproteobacteria;Rickettsiales;Mitochondria	0.168378	0.225507	DDC = Control = BDL
57	Bacteria;Firmicutes;Clostridia;Oscillospirales;[Eubacterium] coprostanoligenes group	0.194364	0.255742	Control = BDL = DDC
58	Bacteria;Firmicutes;Bacilli;Staphylococcales;Staphylococcaceae	0.235989	0.305158	NA
59	Bacteria;Firmicutes;Bacilli;Lactobacillales;Carnobacteriaceae	0.241929	0.307537	NA
60	Bacteria;Actinobacteriota;Coriobacteriia;Coriobacteriales;?	0.261827	0.327284	NA
61	Bacteria;Cyanobacteria;Cyanobacteriia;Chloroplast;?	0.275046	0.338172	NA
62	Bacteria;Actinobacteriota;Actinobacteria;Micrococcales;Micrococcaceae	0.284649	0.344333	NA
63	Bacteria;Firmicutes;Bacilli;RF39;?	0.343607	0.409056	NA
64	Bacteria;Firmicutes;Clostridia;Peptostreptococcales-Tissierellales;Peptostreptococcaceae	0.374586	0.422713	NA
65	Bacteria;Actinobacteriota;Coriobacteriia;Coriobacteriales;Coriobacteriaceae	0.388896	0.422713	NA
66	Bacteria;Bacteroidota;Bacteroidia;Bacteroidales;Marinifilaceae	0.388896	0.422713	NA
67	Bacteria;Cyanobacteria;Vampirivibrionia;Gastranaerophilales;gut metagenome	0.388896	0.422713	NA

68	Bacteria;Proteobacteria;Alphaproteobacteria;Rhizobiales;Beijerinckiaceae	0.388896	0.422713	NA
69	Bacteria;Proteobacteria;Gammaproteobacteria;Pseudomonadales;?	0.388896	0.422713	NA
70	Bacteria;Proteobacteria;Gammaproteobacteria;Burkholderiales;Sutterellaceae	0.4412	0.472714	NA
71	Bacteria;?;?;?;?	0.454436	0.480038	NA
72	Bacteria;Firmicutes;Clostridia;Peptostreptococcales-Tissierellales;?	0.574681	0.598626	NA
73	Bacteria;Firmicutes;Bacilli;?;?	0.853157	0.876531	NA
74	Bacteria;Bacteroidota;Bacteroidia;Bacteroidales;Rikenellaceae	0.917536	0.929935	NA
75	Bacteria;Firmicutes;Clostridia;Lachnospirales;Lachnospiraceae	0.932469	0.932469	NA

Genus

Rank	Feature name	p-value	q-value	Direction
1	Bacteria;Firmicutes;Bacilli;Erysipelotrichales;Erysipelatoclostridiaceae;Erysipelatoclostridium	1.44E-05	0.000993	Control>>>BDL = DDC
2	Bacteria;Proteobacteria;Gammaproteobacteria;Burkholderiales;?;?	1.44E-05	0.000993	Control>>>BDL = DDC
3	Bacteria;Firmicutes;Clostridia;Clostridia vadinBB60 group;uncultured Clostridiales bacterium;?	4.38E-05	0.00147	Control>>>BDL = DDC
4	Bacteria;Deferribacterota;Deferribacteres;Deferribacterales;Deferribacteraceae;Mucispirillum	4.62E-05	0.00147	Control>> BDL > DDC
5	Bacteria;Firmicutes;Bacilli;Lactobacillales;Enterococcaceae;Enterococcus	6.21E-05	0.00147	DDC>> BDL>>>Control
6	Bacteria;Firmicutes;Clostridia;Lachnospirales;Lachnospiraceae;Lachnospiraceae FCS020 group	6.39E-05	0.00147	Control>>>BDL>= DDC
7	Bacteria;Firmicutes;Clostridia;Oscillospirales;Ruminococcaceae;Anaerotruncus	7.85E-05	0.001547	Control>>>BDL > DDC
8	Bacteria;Bacteroidota;Bacteroidia;?;?;?	0.000114	0.001881	Control>> BDL = DDC
9	Bacteria;Firmicutes;Clostridia;Lachnospirales;Lachnospiraceae;Roseburia	0.000123	0.001881	Control>> BDL = DDC
10	Bacteria;Actinobacteriota;Actinobacteria;Corynebacteriales;Corynebacteriaceae;Corynebacterium	0.000169	0.002173	BDL>>>Control > DDC
11	Bacteria;Proteobacteria;Gammaproteobacteria;Enterobacterales;Enterobacteriaceae;Escherichia-Shigella	0.000175	0.002173	DDC = BDL>>>Control
12	Bacteria;Firmicutes;Clostridia;Peptostreptococcales-Tissierellales;Anaerovoracaceae;[Eubacterium] brachy group	0.000189	0.002173	Control > BDL > DDC
13	Bacteria;Firmicutes;Clostridia;Lachnospirales;Lachnospiraceae;Blautia	0.000306	0.00325	BDL = DDC>>>Control
14	Bacteria;Deferribacterota;Deferribacteres;Deferribacterales;Deferribacteraceae;?	0.000353	0.003476	Control>> BDL > DDC
15	Bacteria;Patescibacteria;Saccharimonadia;Saccharimonadales;Saccharimonadaceae;?	0.000399	0.003669	Control>= BDL>> DDC
16	Bacteria;Bacteroidota;Bacteroidia;Bacteroidales;Muribaculaceae;?	0.000476	0.004006	BDL = Control>>>DDC
17	Bacteria;Verrucomicrobiota;Verrucomicrobiae;Verrucomicrobiales;Akkermansiaceae;?	0.000493	0.004006	Control>> BDL>= DDC
18	Bacteria;Bacteroidota;Bacteroidia;Bacteroidales;Rikenellaceae;?	0.000525	0.004028	BDL>> DDC = Control
19	Bacteria;Bacteroidota;Bacteroidia;Bacteroidales;Muribaculaceae;uncultured organism	0.000654	0.004671	Control>> BDL = DDC
20	Bacteria;Firmicutes;Clostridia;Oscillospirales;Ruminococcaceae;Incertae Sedis	0.000681	0.004671	Control>= BDL > DDC

21	Bacteria;Firmicutes;Bacilli;Erysipelotrichales;Erysipelotrichaceae;?	0.000711	0.004671	Control>> DDC = BDL
22	Bacteria;Firmicutes;Bacilli;Erysipelotrichales;Erysipelotrichaceae;Faecalibaculum	0.000883	0.005541	Control>> DDC = BDL
23	Bacteria;Actinobacteriota;Actinobacteria;Micrococcales;Microbacteriaceae;?	0.00098	0.00588	DDC>> Control = BDL
24	Bacteria;Firmicutes;Bacilli;Erysipelotrichales;Erysipelatoclostridiaceae;Candidatus Stoquefichus	0.001361	0.007824	Control>> BDL = DDC
25	Bacteria;Firmicutes;Clostridia;Lachnospirales;?;?	0.00173	0.009394	Control>>>DDC = BDL
26	Bacteria;Firmicutes;?;?;?;?	0.001789	0.009394	Control>> DDC = BDL
27	Bacteria;Desulfobacterota;Desulfovibrionia;Desulfovibrionales;Desulfovibrionaceae;Desulfovibrio	0.001912	0.009394	Control>= DDC > BDL
28	Bacteria;Bacteroidota;Bacteroidia;Bacteroidales;Tannerellaceae;Parabacteroides	0.001969	0.009394	DDC > BDL = Control
29	Bacteria;Cyanobacteria;Vampirivibrionia;Gastranaerophilales;?;?	0.001974	0.009394	Control>> BDL = DDC
30	Bacteria;Bacteroidota;Bacteroidia;Bacteroidales;Muribaculaceae;mouse gut metagenome	0.003491	0.01606	Control = DDC = BDL
31	Bacteria;Firmicutes;Bacilli;Lactobacillales;Lactobacillaceae;Ligilactobacillus	0.003691	0.016433	DDC = BDL>> Control
32	Bacteria;Firmicutes;Clostridia;Oscillospirales;Oscillospiraceae;UCG-005	0.003925	0.016924	BDL > Control = DDC
33	Bacteria;Proteobacteria;Gammaproteobacteria;Enterobacterales;Enterobacteriaceae;?	0.004442	0.018576	BDL = DDC > Control
34	Bacteria;Firmicutes;Clostridia;Oscillospirales;Ruminococcaceae;?	0.005331	0.021638	Control = BDL = DDC
35	Bacteria;Bacteroidota;Bacteroidia;Bacteroidales;Bacteroidaceae;Bacteroides	0.005884	0.023201	DDC = BDL>>>Control
36	Bacteria;Firmicutes;Clostridia;Clostridia vadinBB60 group;uncultured organism;?	0.006683	0.025618	Control > BDL = DDC
37	Bacteria;Firmicutes;Bacilli;Staphylococcales;Staphylococcaceae;Staphylococcus	0.010443	0.037639	DDC = Control = BDL
38	Bacteria;Firmicutes;Clostridia;Lachnospirales;Lachnospiraceae;[Eubacterium] xylanophilum group	0.011433	0.037639	Control = BDL = DDC
39	Bacteria;Firmicutes;Clostridia;Lachnospirales;Lachnospiraceae;Lachnospiraceae UCG-008	0.013589	0.037639	Control = BDL = DDC
40	Bacteria;Firmicutes;Bacilli;Acholeplasmatales;Acholeplasmataceae;?	0.014183	0.037639	Control > BDL > DDC
41	Bacteria;Firmicutes;Bacilli;Staphylococcales;Staphylococcaceae;Jeotgalicoccus	0.014183	0.037639	Control > BDL > DDC
42	Bacteria;Firmicutes;Clostridia;Christensenellales;Christensenellaceae;?	0.014183	0.037639	Control > BDL > DDC
43	Bacteria;Firmicutes;Clostridia;Clostridiales;?;?	0.014183	0.037639	Control > BDL > DDC
44	Bacteria;Firmicutes;Clostridia;Clostridiales;Clostridiaceae;?	0.014183	0.037639	Control > BDL > DDC
45	Bacteria;Firmicutes;Clostridia;Lachnospirales;Lachnospiraceae;Tyzzerella	0.014183	0.037639	Control > BDL > DDC
46	Bacteria;Firmicutes;Clostridia;Oscillospirales;?;metagenome	0.014183	0.037639	Control > BDL > DDC
47	Bacteria;Firmicutes;Clostridia;Oscillospirales;Butyricicoccaceae;UCG-009	0.014183	0.037639	Control > BDL > DDC
48	Bacteria;Firmicutes;Clostridia;Oscillospirales;Oscillospiraceae;NK4A214 group	0.014183	0.037639	Control > BDL > DDC
49	Bacteria;Firmicutes;Clostridia;Oscillospirales;Ruminococcaceae;Harryflintia	0.014183	0.037639	Control > BDL > DDC
50	Bacteria;Firmicutes;Clostridia;Oscillospirales;UCG-010;?	0.014183	0.037639	Control > BDL > DDC
51	Bacteria;Firmicutes;Clostridia;Peptostreptococcales-Tissierellales;Anaerovoracaceae;?	0.014183	0.037639	Control > BDL > DDC
52	Bacteria;Proteobacteria;Gammaproteobacteria;Pseudomonadales;Pseudomonadaceae;Pseudomonas	0.014183	0.037639	Control > BDL > DDC

53	Bacteria;Firmicutes;Bacilli;Lactobacillales;Lactobacillaceae;?	0.016041	0.041768	BDL = DDC = Control
54	Bacteria;Actinobacteriota;Coriobacteriia;Coriobacteriales;Eggerthellaceae;?	0.017106	0.043717	Control = BDL = DDC
55	Bacteria;Firmicutes;Clostridia;Lachnospirales;Lachnospiraceae;Lachnospiraceae NK4A136 group	0.01936	0.048575	Control = BDL = DDC
56	Bacteria;Firmicutes;Clostridia;Lachnospirales;Lachnospiraceae;Lachnospiraceae UCG-001	0.020909	0.05148	Control = BDL > DDC
57	Bacteria;Firmicutes;Bacilli;Lactobacillales;Streptococcaceae;Streptococcus	0.021264	0.05148	BDL>= Control = DDC
58	Bacteria;Proteobacteria;Alphaproteobacteria;Rhodospirillales;?;?	0.02222	0.05263	DDC = BDL>= Control
59	Bacteria;Bacteroidota;Bacteroidia;Bacteroidales;?;?	0.022501	0.05263	Control = BDL = DDC
60	Bacteria;Firmicutes;Clostridia;Oscillospirales;?;?	0.023026	0.05296	Control = BDL = DDC
61	Bacteria;Firmicutes;Clostridia;?;?;?	0.027019	0.060903	BDL = DDC>>>Control
62	Bacteria;Firmicutes;Clostridia;Oscillospirales;Oscillospiraceae;Colidextribacter	0.027362	0.060903	Control>> DDC = BDL
63	Bacteria;Firmicutes;Clostridia;Clostridia vadinBB60 group;?;?	0.032135	0.070392	Control > DDC = BDL
64	Bacteria;Proteobacteria;Gammaproteobacteria;Burkholderiales;Sutterellaceae;?	0.034442	0.074266	Control > BDL = DDC
65	Bacteria;Firmicutes;Clostridia;Oscillospirales;Oscillospiraceae;?	0.042068	0.085035	Control = BDL = DDC
66	Bacteria;Actinobacteriota;Actinobacteria;Corynebacteriales;Corynebacteriaceae;?	0.042517	0.085035	Control = BDL = DDC
67	Bacteria;Firmicutes;Bacilli;Acholeplasmatales;Acholeplasmataceae;Anaeroplasma	0.042517	0.085035	Control = BDL = DDC
68	Bacteria;Firmicutes;Clostridia;Oscillospirales;Ruminococcaceae;Ruminococcus	0.042517	0.085035	Control = BDL = DDC
69	Bacteria;Patescibacteria;Saccharimonadia;Saccharimonadales;Saccharimonadaceae;Candidatus Saccharimonas	0.042517	0.085035	Control = BDL = DDC
70	Bacteria;Firmicutes;Bacilli;Lactobacillales;?;?	0.043854	0.085754	Control > DDC = BDL
71	Bacteria;Firmicutes;Bacilli;RF39;uncultured rumen bacterium;?	0.046605	0.085754	Control>= BDL>= DDC
72	Bacteria;Firmicutes;Clostridia;Christensenellales;?;?	0.046605	0.085754	Control>= BDL>= DDC
73	Bacteria;Firmicutes;Clostridia;Oscillospirales;Butyricicoccaceae;?	0.046605	0.085754	Control>= BDL>= DDC
74	Bacteria;Firmicutes;Clostridia;Oscillospirales;[Eubacterium] coprostanoligenes group;?	0.046605	0.085754	Control>= BDL>= DDC
75	Bacteria;Firmicutes;Clostridia;Peptococcales;Peptococcaceae;Peptococcus	0.046605	0.085754	Control>= BDL>= DDC
76	Bacteria;Firmicutes;Clostridia;Lachnospirales;Lachnospiraceae;Lachnospiraceae UCG-006	0.058245	0.10576	Control = BDL>= DDC
77	Bacteria;Firmicutes;Bacilli;Staphylococcales;Staphylococcaceae;?	0.059371	0.106405	DDC = BDL = Control
78	Bacteria;Verrucomicrobiota;Verrucomicrobiae;Verrucomicrobiales;?;?	0.06092	0.107781	DDC = BDL = Control
79	Bacteria;Firmicutes;Clostridia;Peptococcales;Peptococcaceae;?	0.064436	0.11256	Control = BDL = DDC
80	Bacteria;Firmicutes;Clostridia;Lachnospirales;Lachnospiraceae;[Eubacterium] ventriosum group	0.078931	0.136155	Control = BDL = DDC
81	Bacteria;Firmicutes;Bacilli;Lactobacillales;Lactobacillaceae;Lactobacillus	0.089188	0.151951	BDL = Control>= DDC
82	Bacteria;Bacteroidota;Bacteroidia;Bacteroidales;Bacteroidaceae;?	0.093891	0.156391	Control = DDC = BDL
83	Bacteria;Actinobacteriota;Actinobacteria;Propionibacteriales;Propionibacteriaceae;Cutibacterium	0.096328	0.156391	Control = BDL = DDC

84	Bacteria;Firmicutes;Bacilli;Lactobacillales;Aerococcaceae;Facklamia	0.096328	0.156391	Control = BDL = DDC
85	Bacteria;Proteobacteria;Gammaproteobacteria;Burkholderiales;Burkholderiaceae;Burkholderia-Caballeronia-Paraburkholderia	0.096328	0.156391	Control = BDL = DDC
86	Bacteria;Firmicutes;Clostridia;Clostridia UCG-014;?;?	0.105936	0.167915	Control = BDL = DDC
87	Bacteria;Actinobacteriota;Coriobacteriia;Coriobacteriales;Atopobiaceae;?	0.106104	0.167915	Control = BDL = DDC
88	Bacteria;Bacteroidota;Bacteroidia;Bacteroidales;Muribaculaceae;Muribaculum	0.107076	0.167915	BDL = Control = DDC
89	Bacteria;Proteobacteria;Gammaproteobacteria;Enterobacterales;?;?	0.114864	0.178104	DDC = Control = BDL
90	Bacteria;Actinobacteriota;Coriobacteriia;Coriobacteriales;Atopobiaceae;Olsenella	0.124544	0.189006	Control = DDC = BDL
91	Bacteria;Actinobacteriota;?;?;?;?	0.124634	0.189006	Control = DDC = BDL
92	Bacteria;Firmicutes;Clostridia;Oscillospirales;Oscillospiraceae;Oscillibacter	0.126957	0.189607	Control = BDL = DDC
93	Bacteria;Firmicutes;Bacilli;Lactobacillales;Lactobacillaceae;Limosilactobacillus	0.127779	0.189607	BDL = DDC = Control
94	Bacteria;Bacteroidota;Bacteroidia;Bacteroidales;Prevotellaceae;Prevotellaceae UCG-001	0.129866	0.190654	Control>= BDL = DDC
95	Bacteria;Firmicutes;Clostridia;Oscillospirales;Butyricicoccaceae;Butyricicoccus	0.133517	0.193523	Control = BDL = DDC
96	noHit;	0.13937	0.193523	Control = BDL = DDC
97	Bacteria;Actinobacteriota;Actinobacteria;Corynebacteriales;Corynebacteriaceae;Lawsonella	0.140234	0.193523	Control = BDL = DDC
98	Bacteria;Actinobacteriota;Actinobacteria;Micrococcales;Micrococcaceae;Kocuria	0.140234	0.193523	Control = BDL = DDC
99	Bacteria;Actinobacteriota;Actinobacteria;Pseudonocardiales;Pseudonocardiaceae;?	0.140234	0.193523	Control = BDL = DDC
100	Bacteria;Bacteroidota;Bacteroidia;Bacteroidales;Rikenellaceae;Rikenellaceae RC9 gut group	0.140234	0.193523	Control = BDL = DDC
101	Bacteria;Firmicutes;Clostridia;Monoglobales;Monoglobaceae;Monoglobus	0.152784	0.208754	Control = BDL = DDC
102	Bacteria;Proteobacteria;Alphaproteobacteria;Rickettsiales;Mitochondria;?	0.168378	0.227806	DDC = Control = BDL
103	Bacteria;Firmicutes;Clostridia;Lachnospirales;Lachnospiraceae;Anaerostipes	0.178181	0.238729	Control = BDL = DDC
104	Bacteria;Bacteroidota;Bacteroidia;Bacteroidales;Tannerellaceae;?	0.200045	0.265445	NA
105	Bacteria;Actinobacteriota;Coriobacteriia;Coriobacteriales;Eggerthellaceae;Enterorhabdus	0.20667	0.271624	NA
106	Bacteria;Firmicutes;Clostridia;Lachnospirales;Lachnospiraceae;A2	0.232094	0.302161	NA
107	Bacteria;Firmicutes;Bacilli;Lactobacillales;Carnobacteriaceae;Atopostipes	0.241929	0.312021	NA
108	Bacteria;Verrucomicrobiota;Verrucomicrobiae;Verrucomicrobiales;Akkermansiaceae;Akkermansia	0.246966	0.315568	NA
109	Bacteria;Actinobacteriota;Actinobacteria;Micrococcales;Micrococcaceae;?	0.259449	0.328474	NA
110	Bacteria;Actinobacteriota;Coriobacteriia;Coriobacteriales;?;?	0.261827	0.328474	NA
111	Bacteria;Cyanobacteria;Cyanobacteriia;Chloroplast;?;?	0.275046	0.34195	NA
112	Bacteria;Proteobacteria;Gammaproteobacteria;Burkholderiales;Burkholderiaceae;Ralstonia	0.324652	0.393	NA
113	Bacteria;Proteobacteria;Gammaproteobacteria;Burkholderiales;Comamonadaceae;?	0.324652	0.393	NA
114	Bacteria;Proteobacteria;Gammaproteobacteria;Burkholderiales;Comamonadaceae;Pelomonas	0.324652	0.393	NA
115	Bacteria;Firmicutes;Bacilli;RF39;?;?	0.343607	0.412328	NA
116	Bacteria;Firmicutes;Clostridia;Oscillospirales;Ruminococcaceae;Faecalibacterium	0.367384	0.425933	NA

117	Bacteria;Desulfobacterota;Desulfovibrionia;Desulfovibrionales;Desulfovibrionaceae;Bilophila	0.374009	0.425933	NA
118	Bacteria;Firmicutes;Clostridia;Peptostreptococcales-Tissierellales;Peptostreptococcaceae;?	0.374586	0.425933	NA
119	Bacteria;Actinobacteriota;Coriobacteriia;Coriobacteriales;Coriobacteriaceae;Collinsella	0.388896	0.425933	NA
120	Bacteria;Bacteroidota;Bacteroidia;Bacteroidales;Marinifilaceae;Odoribacter	0.388896	0.425933	NA
121	Bacteria;Cyanobacteria;Vampirivibrionia;Gastranaerophilales;gut metagenome;?	0.388896	0.425933	NA
122	Bacteria;Firmicutes;Clostridia;Christensenellales;Christensenellaceae;Christensenella sp. Marseille-P2437	0.388896	0.425933	NA
123	Bacteria;Firmicutes;Clostridia;Lachnospirales;Lachnospiraceae;Marvinbryantia	0.388896	0.425933	NA
124	Bacteria;Firmicutes;Clostridia;Oscillospirales;[Eubacterium] coprostanoligenes group;gut metagenome	0.388896	0.425933	NA
125	Bacteria;Proteobacteria;Alphaproteobacteria;Rhizobiales;Beijerinckiaceae;Methylobacterium-Methylorubrum	0.388896	0.425933	NA
126	Bacteria;Proteobacteria;Gammaproteobacteria;Pseudomonadales;?;?	0.388896	0.425933	NA
127	Bacteria;Bacteroidota;Bacteroidia;Bacteroidales;Muribaculaceae;uncultured Bacteroidales bacterium	0.437373	0.475256	NA
128	Bacteria;?;?;?;?;?	0.454436	0.489938	NA
129	Bacteria;Bacteroidota;Bacteroidia;Bacteroidales;Prevotellaceae;?	0.476534	0.509781	NA
130	Bacteria;Proteobacteria;Gammaproteobacteria;Burkholderiales;Sutterellaceae;Parasutterella	0.491938	0.522212	NA
131	Bacteria;Firmicutes;Bacilli;Lactobacillales;Lactobacillaceae;HT002	0.507641	0.534767	NA
132	Bacteria;Desulfobacterota;Desulfovibrionia;Desulfovibrionales;Desulfovibrionaceae;?	0.519055	0.542648	NA
133	Bacteria;Firmicutes;Clostridia;Peptostreptococcales-Tissierellales;?;?	0.574681	0.596285	NA
134	Bacteria;Actinobacteriota;Coriobacteriia;Coriobacteriales;Eggerthellaceae;Parvibacter	0.628484	0.647245	NA
135	Bacteria;Firmicutes;Clostridia;Lachnospirales;Lachnospiraceae;?	0.767239	0.784288	NA
136	Bacteria;Bacteroidota;Bacteroidia;Bacteroidales;Rikenellaceae;Alistipes	0.850107	0.859384	NA
137	Bacteria;Firmicutes;Bacilli;?;?;?	0.853157	0.859384	NA
138	Bacteria;Firmicutes;Clostridia;Lachnospirales;Lachnospiraceae;Lachnoclostridium	0.956688	0.956688	NA

Species

Rank	Feature name	p-value	q-value	Direction
1	Bacteria;Firmicutes;Bacilli;Erysipelotrichales;Erysipelatoclostridiaceae;Erysipelatoclostridium;?	1.44E-05	0.000816	Control>>>BDL = DDC
2	Bacteria;Firmicutes;Clostridia;Lachnospirales;Lachnospiraceae;Lachnoclostridium;bacterium NLAE-zl-H31	1.44E-05	0.000816	Control>>>BDL = DDC
3	Bacteria;Proteobacteria;Gammaproteobacteria;Burkholderiales;?;?;?	1.44E-05	0.000816	Control>>>BDL = DDC
4	Bacteria;Firmicutes;Clostridia;Clostridia vadinBB60 group;uncultured Clostridiales bacterium;?;?	4.38E-05	0.001552	Control>>>BDL = DDC
5	Bacteria;Deferribacterota;Deferribacteres;Deferribacterales;Deferribacteraceae;Mucispirillum;Mucispirillum schaedleri	4.62E-05	0.001552	Control>> BDL > DDC
6	Bacteria;Firmicutes;Bacilli;Lactobacillales;Enterococcaceae;Enterococcus;Enterococcus faecalis	6.21E-05	0.001552	DDC>> BDL>>>Control
7	Bacteria;Firmicutes;Clostridia;Lachnospirales;Lachnospiraceae;Lachnospiraceae FCS020 group;?	6.39E-05	0.001552	Control>>>BDL>= DDC

8	Bacteria;Firmicutes;Clostridia;Oscillospirales;Ruminococcaceae;Anaerotruncus;?	7.85E-05	0.001668	Control>>>BDL > DDC
9	Bacteria;Bacteroidota;Bacteroidia;?;?;?;?	0.000114	0.002061	Control>> BDL = DDC
10	Bacteria;Firmicutes;Clostridia;Lachnospirales;Lachnospiraceae;Roseburia;?	0.000123	0.002061	Control>> BDL = DDC
11	Bacteria;Firmicutes;Bacilli;Erysipelotrichales;Erysipelotrichaceae;Faecalibaculum;Faecalibaculum rodentium	0.000133	0.002061	Control>>>BDL = DDC
12	Bacteria;Proteobacteria;Gammaproteobacteria;Enterobacterales;Enterobacteriaceae;Escherichia-Shigella;Escherichia coli	0.000175	0.002471	DDC = BDL>>>Control
13	Bacteria;Firmicutes;Clostridia;Peptostreptococcales-Tissierellales;Anaerovoracaceae;[Eubacterium] brachy group;?	0.000189	0.002471	Control > BDL > DDC
14	Bacteria;Firmicutes;Bacilli;Lactobacillales;Lactobacillaceae;Lactobacillus;Lactobacillus gasseri	0.000261	0.003165	Control>> BDL = DDC
15	Bacteria;Firmicutes;Clostridia;Lachnospirales;Lachnospiraceae;Blautia;?	0.000313	0.00355	DDC = BDL>>>Control
16	Bacteria;Deferribacterota;Deferribacteres;Deferribacterales;Deferribacteraceae;?;?	0.000353	0.003747	Control>> BDL > DDC
17	Bacteria;Patescibacteria;Saccharimonadia;Saccharimonadales;Saccharimonadaceae;?;?	0.000399	0.003988	Control>= BDL>> DDC
18	Bacteria;Bacteroidota;Bacteroidia;Bacteroidales;Muribaculaceae;?;?	0.000476	0.004415	BDL = Control>>> DDC
19	Bacteria;Verrucomicrobiota;Verrucomicrobiae;Verrucomicrobiales;Akkermansiaceae;?;?	0.000493	0.004415	Control>> BDL>= DDC
20	Bacteria;Bacteroidota;Bacteroidia;Bacteroidales;Rikenellaceae;?;?	0.000525	0.004466	BDL>> DDC = Control
21	Bacteria;Firmicutes;Bacilli;Erysipelotrichales;Erysipelotrichaceae;Faecalibaculum;?	0.000589	0.004764	Control>> DDC = BDL
22	Bacteria;Bacteroidota;Bacteroidia;Bacteroidales;Rikenellaceae;Alistipes;Alistipes obesi	0.000628	0.004834	BDL > Control > DDC
23	Bacteria;Bacteroidota;Bacteroidia;Bacteroidales;Muribaculaceae;uncultured organism;?	0.000654	0.004834	Control>> BDL = DDC
24	Bacteria;Firmicutes;Bacilli;Erysipelotrichales;Erysipelotrichaceae;?;?	0.000711	0.005035	Control>> DDC = BDL
25	Bacteria;Firmicutes;Clostridia;Oscillospirales;Ruminococcaceae;Incertae Sedis;?	0.000768	0.005219	Control = BDL > DDC
26	Bacteria;Actinobacteriota;Actinobacteria;Micrococcales;Microbacteriaceae;?;?	0.00098	0.006408	DDC>> Control = BDL
27	Bacteria;Firmicutes;Bacilli;Erysipelotrichales;Erysipelatoclostridiaceae;Candidatus Stoquefichus;?	0.001361	0.008262	Control>> BDL = DDC
28	Bacteria;Firmicutes;Clostridia;Lachnospirales;Lachnospiraceae;Anaerostipes;Anaerostipes caccae	0.001361	0.008262	Control>> BDL = DDC
29	Bacteria;Firmicutes;Clostridia;Lachnospirales;?;?;?	0.00173	0.010139	Control>>> DDC = BDL
30	Bacteria;Firmicutes;?;?;?;?;?	0.001789	0.010139	Control>> DDC = BDL
31	Bacteria;Desulfobacterota;Desulfovibrionia;Desulfovibrionales;Desulfovibrionaceae;Desulfovibrio;?	0.001912	0.010485	Control>= DDC > BDL
32	Bacteria;Cyanobacteria;Vampirivibrionia;Gastranaerophilales;?;?;?	0.001974	0.010488	Control>> BDL = DDC
33	Bacteria;Bacteroidota;Bacteroidia;Bacteroidales;Tannerellaceae;Parabacteroides;Parabacteroides goldsteinii	0.003214	0.016557	DDC > BDL = Control
34	Bacteria;Bacteroidota;Bacteroidia;Bacteroidales;Muribaculaceae;mouse gut metagenome;?	0.003491	0.017284	Control = DDC = BDL
35	Bacteria;Firmicutes;Bacilli;Lactobacillales;Lactobacillaceae;Ligilactobacillus;?	0.003691	0.017284	DDC = BDL>> Control
36	Bacteria;Firmicutes;Clostridia;Lachnospirales;Lachnospiraceae;Lachnospiraceae NK4A136 group;Clostridiales bacterium	0.003712	0.017284	Control = BDL>= DDC
37	Bacteria;Proteobacteria;Gammaproteobacteria;Enterobacterales;Enterobacteriaceae;Escherichia-Shigella;?	0.003762	0.017284	Control>= BDL = DDC

38	Bacteria;Firmicutes;Clostridia;Oscillospirales;Oscillospiraceae;UCG-005;?	0.003925	0.017557	BDL > Control = DDC
39	Bacteria;Proteobacteria;Gammaproteobacteria;Enterobacterales;Enterobacteriaceae;?;?	0.004442	0.019363	BDL = DDC > Control
40	Bacteria;Verrucomicrobiota;Verrucomicrobiae;Verrucomicrobiales;Akkermansiaceae;Akkermansia;Akkermansia muciniphila	0.004688	0.019924	Control>> DDC = BDL
41	Bacteria;Bacteroidota;Bacteroidia;Bacteroidales;Tannerellaceae;Parabacteroides;Parabacteroides distasonis	0.00507	0.021022	DDC>= BDL = Control
42	Bacteria;Firmicutes;Clostridia;Oscillospirales;Ruminococcaceae;?;?	0.005331	0.021578	Control = BDL = DDC
43	Bacteria;Firmicutes;Bacilli;Staphylococcales;Staphylococcaceae;Staphylococcus;Staphylococcus lentus	0.005676	0.02244	DDC > Control = BDL
44	Bacteria;Firmicutes;Clostridia;Oscillospirales;Ruminococcaceae;Incertae Sedis;Acutalibacter muris	0.006287	0.024289	Control > BDL = DDC
45	Bacteria;Actinobacteriota;Coriobacteriia;Coriobacteriales;Eggerthellaceae;?;?	0.006619	0.024698	Control = BDL = DDC
46	Bacteria;Firmicutes;Clostridia;Clostridia vadinBB60 group;uncultured organism;?;?	0.006683	0.024698	Control > BDL = DDC
47	Bacteria;Actinobacteriota;Coriobacteriia;Coriobacteriales;Eggerthellaceae;Enterorhabdus;mouse gut	0.006925	0.025049	Control = BDL = DDC
48	Bacteria;Firmicutes;Clostridia;Lachnospirales;Lachnospiraceae;[Eubacterium] xylanophilum group;?	0.011433	0.037673	Control = BDL = DDC
49	Bacteria;Bacteroidota;Bacteroidia;Bacteroidales;Bacteroidaceae;Bacteroides;Bacteroides uniformis	0.012849	0.037673	DDC = BDL > Control
50	Bacteria;Firmicutes;Clostridia;Lachnospirales;Lachnospiraceae;Lachnospiraceae UCG-008;?	0.013589	0.037673	Control = BDL = DDC
51	Bacteria;Firmicutes;Bacilli;Acholeplasmatales;Acholeplasmataceae;?;?	0.014183	0.037673	Control > BDL > DDC
52	Bacteria;Firmicutes;Bacilli;Staphylococcales;Staphylococcaceae;Jeotgalicoccus;?	0.014183	0.037673	Control > BDL > DDC
53	Bacteria;Firmicutes;Clostridia;Christensenellales;Christensenellaceae;?;?	0.014183	0.037673	Control > BDL > DDC
54	Bacteria;Firmicutes;Clostridia;Clostridiales;?;?;?	0.014183	0.037673	Control > BDL > DDC
55	Bacteria;Firmicutes;Clostridia;Clostridiales;Clostridiaceae;?;?	0.014183	0.037673	Control > BDL > DDC
56	Bacteria;Firmicutes;Clostridia;Lachnospirales;Lachnospiraceae;Blautia;Lachnospiraceae bacterium	0.014183	0.037673	Control > BDL > DDC
57	Bacteria;Firmicutes;Clostridia;Lachnospirales;Lachnospiraceae;Tyzzerella;?	0.014183	0.037673	Control > BDL > DDC
58	Bacteria;Firmicutes;Clostridia;Oscillospirales;?;metagenome;?	0.014183	0.037673	Control > BDL > DDC
59	Bacteria;Firmicutes;Clostridia;Oscillospirales;Butyricicoccaceae;UCG-009;[Clostridium] leptum	0.014183	0.037673	Control > BDL > DDC
60	Bacteria;Firmicutes;Clostridia;Oscillospirales;Oscillospiraceae;NK4A214 group;?	0.014183	0.037673	Control > BDL > DDC
61	Bacteria;Firmicutes;Clostridia;Oscillospirales;Ruminococcaceae;Harryflintia;?	0.014183	0.037673	Control > BDL > DDC
62	Bacteria;Firmicutes;Clostridia;Oscillospirales;UCG-010;?;?	0.014183	0.037673	Control > BDL > DDC
63	Bacteria;Firmicutes;Clostridia;Peptostreptococcales-Tissierellales;Anaerovoracaceae;?;?	0.014183	0.037673	Control > BDL > DDC
64	Bacteria;Proteobacteria;Gammaproteobacteria;Pseudomonadales;Pseudomonadaceae;Pseudomonas;?	0.014183	0.037673	Control > BDL > DDC
65	Bacteria;Firmicutes;Bacilli;Lactobacillales;Lactobacillaceae;?;?	0.016041	0.041954	BDL = DDC = Control
66	Bacteria;Firmicutes;Bacilli;Lactobacillales;Lactobacillaceae;Lactobacillus;?	0.017354	0.044698	Control>= BDL = DDC
67	Bacteria;Bacteroidota;Bacteroidia;Bacteroidales;Rikenellaceae;Alistipes;Alistipes timonensis	0.018211	0.046206	DDC = BDL > Control
68	Bacteria;Firmicutes;Clostridia;Lachnospirales;Lachnospiraceae;Lachnospiraceae NK4A136 group;?	0.01936	0.048399	Control = BDL = DDC
69	Bacteria;Firmicutes;Clostridia;Lachnospirales;Lachnospiraceae;Lachnospiraceae UCG-001;?	0.020909	0.051515	Control = BDL > DDC

70	Bacteria;Firmicutes;Bacilli;Lactobacillales;Streptococcaceae;Streptococcus;?	0.021264	0.05164	BDL>= Control = DDC
71	Bacteria;Bacteroidota;Bacteroidia;Bacteroidales;Tannerellaceae;Parabacteroides;?	0.021883	0.052395	Control = DDC>= BDL
72	Bacteria;Proteobacteria;Alphaproteobacteria;Rhodospirillales;?;?;?	0.02222	0.0524	DDC = BDL>= Control
73	Bacteria;Bacteroidota;Bacteroidia;Bacteroidales;?;?;?	0.022501	0.0524	Control = BDL = DDC
74	Bacteria;Firmicutes;Clostridia;Oscillospirales;?;?;?	0.023026	0.052898	Control = BDL = DDC
75	Bacteria;Firmicutes;Bacilli;Lactobacillales;Carnobacteriaceae;Atopostipes;?	0.025934	0.058784	Control>= BDL = DDC
76	Bacteria;Firmicutes;Clostridia;?;?;?;?	0.027019	0.06041	BDL = DDC>>>Control
77	Bacteria;Firmicutes;Clostridia;Oscillospirales;Oscillospiraceae;Colidextribacter;?	0.027362	0.06041	Control>> DDC = BDL
78	Bacteria;Actinobacteriota;Actinobacteria;Corynebacteriales;Corynebacteriaceae;Corynebacterium;Corynebacterium lowii	0.02849	0.062094	BDL>= Control = DDC
79	Bacteria;Firmicutes;Clostridia;Clostridia vadinBB60 group;?;?;?	0.032135	0.069152	Control > DDC = BDL
80	Bacteria;Proteobacteria;Gammaproteobacteria;Burkholderiales;Sutterellaceae;?;?	0.034442	0.07319	Control > BDL = DDC
81	Bacteria;Bacteroidota;Bacteroidia;Bacteroidales;Bacteroidaceae;Bacteroides;Bacteroides acidifaciens	0.036893	0.077429	BDL > Control = DDC
82	Bacteria;Firmicutes;Clostridia;Oscillospirales;Oscillospiraceae;?;?	0.042068	0.084046	Control = BDL = DDC
83	Bacteria;Actinobacteriota;Actinobacteria;Corynebacteriales;Corynebacteriaceae;?;?	0.042517	0.084046	Control = BDL = DDC
84	Bacteria;Firmicutes;Bacilli;Acholeplasmatales;Acholeplasmataceae;Anaeroplasma;?	0.042517	0.084046	Control = BDL = DDC
85	Bacteria;Firmicutes;Clostridia;Oscillospirales;Ruminococcaceae;Ruminococcus;?	0.042517	0.084046	Control = BDL = DDC
86	Bacteria;Patescibacteria;Saccharimonadia;Saccharimonadales;Saccharimonadaceae;Candidatus Saccharimonas;?	0.042517	0.084046	Control = BDL = DDC
87	Bacteria;Firmicutes;Bacilli;Lactobacillales;?;?;?	0.043854	0.085691	Control > DDC = BDL
88	Bacteria;Firmicutes;Bacilli;RF39;uncultured rumen bacterium;?;?	0.046605	0.086119	Control>= BDL>= DDC
89	Bacteria;Firmicutes;Clostridia;Christensenellales;?;?;?	0.046605	0.086119	Control>= BDL>= DDC
90	Bacteria;Firmicutes;Clostridia;Oscillospirales;Butyricicoccaceae;?;?	0.046605	0.086119	Control>= BDL>= DDC
91	Bacteria;Firmicutes;Clostridia;Oscillospirales;[Eubacterium] coprostanoligenes group;?;?	0.046605	0.086119	Control>= BDL>= DDC
92	Bacteria;Firmicutes;Clostridia;Peptococcales;Peptococcaceae;Peptococcus;?	0.046605	0.086119	Control>= BDL>= DDC
93	Bacteria;Actinobacteriota;Actinobacteria;Corynebacteriales;Corynebacteriaceae;Corynebacterium;Corynebacterium ammoniagenes	0.050319	0.091452	Control = BDL = DDC
94	Bacteria;Bacteroidota;Bacteroidia;Bacteroidales;Bacteroidaceae;Bacteroides;Bacteroides caecimuris	0.050567	0.091452	DDC = BDL = Control
95	Bacteria;Firmicutes;Clostridia;Lachnospirales;Lachnospiraceae;Lachnoclostridium;?	0.057212	0.102379	BDL = DDC > Control
96	Bacteria;Firmicutes;Clostridia;Lachnospirales;Lachnospiraceae;Lachnospiraceae UCG-006;?	0.058245	0.103141	Control = BDL>= DDC
97	Bacteria;Firmicutes;Bacilli;Staphylococcales;Staphylococcaceae;?;?	0.059371	0.104052	DDC = BDL = Control
98	Bacteria;Verrucomicrobiota;Verrucomicrobiae;Verrucomicrobiales;?;?;?	0.06092	0.105677	DDC = BDL = Control
99	Bacteria;Firmicutes;Clostridia;Peptococcales;Peptococcaceae;?;?	0.064436	0.110648	Control = BDL = DDC

100	Bacteria;Firmicutes;Clostridia;Lachnospirales;Lachnospiraceae;Lachnoclostridium;[Clostridium] bolteae	0.075773	0.128814	DDC = Control = BDL
101	Bacteria;Firmicutes;Clostridia;Lachnospirales;Lachnospiraceae;[Eubacterium] ventriosum group;?	0.078931	0.132854	Control = BDL = DDC
102	Bacteria;Actinobacteriota;Actinobacteria;Corynebacteriales;Corynebacteriaceae;Corynebacterium;?	0.08468	0.141133	Control = BDL = DDC
103	Bacteria;Firmicutes;Bacilli;Lactobacillales;Lactobacillaceae;Lactobacillus;Lactobacillus taiwanensis	0.089188	0.147204	BDL = Control>= DDC
104	Bacteria;Bacteroidota;Bacteroidia;Bacteroidales;Muribaculaceae;Muribaculum;Muribaculum intestinale	0.091265	0.149184	Control = BDL = DDC
105	Bacteria;Bacteroidota;Bacteroidia;Bacteroidales;Bacteroidaceae;?;?	0.093891	0.152014	Control = DDC = BDL
106	Bacteria;Actinobacteriota;Actinobacteria;Propionibacteriales;Propionibacteriaceae;Cutibacterium;Cutibacterium granulosum	0.096328	0.153044	Control = BDL = DDC
107	Bacteria;Firmicutes;Bacilli;Lactobacillales;Aerococcaceae;Facklamia;Facklamia tabacinasalis	0.096328	0.153044	Control = BDL = DDC
108	Bacteria;Actinobacteriota;Coriobacteriia;Coriobacteriales;Eggerthellaceae;?;uncultured Coriobacteriales bacterium	0.100321	0.157913	Control = DDC = BDL
109	Bacteria;Firmicutes;Clostridia;Clostridia UCG-014;?;?;?	0.105936	0.163979	Control = BDL = DDC
110	Bacteria;Actinobacteriota;Coriobacteriia;Coriobacteriales;Atopobiaceae;?;?	0.106104	0.163979	Control = BDL = DDC
111	Bacteria;Bacteroidota;Bacteroidia;Bacteroidales;Muribaculaceae;Muribaculum;?	0.107076	0.163991	BDL = Control = DDC
112	Bacteria;Proteobacteria;Gammaproteobacteria;Enterobacterales;?;?;?	0.114864	0.174347	DDC = Control = BDL
113	Bacteria;Actinobacteriota;Coriobacteriia;Coriobacteriales;Atopobiaceae;Olsenella;?	0.124544	0.185858	Control = DDC = BDL
114	Bacteria;Actinobacteriota;?;?;?;?;?	0.124634	0.185858	Control = DDC = BDL
115	Bacteria;Firmicutes;Clostridia;Oscillospirales;Oscillospiraceae;Oscillibacter;Clostridiales bacterium	0.126957	0.187262	Control = BDL = DDC
116	Bacteria;Firmicutes;Bacilli;Lactobacillales;Lactobacillaceae;Limosilactobacillus;?	0.127779	0.187262	BDL = DDC = Control
117	Bacteria;Bacteroidota;Bacteroidia;Bacteroidales;Prevotellaceae;Prevotellaceae UCG-001;?	0.129866	0.188694	Control>= BDL = DDC
118	Bacteria;Firmicutes;Clostridia;Oscillospirales;Butyricicoccaceae;Butyricicoccus;?	0.133517	0.192355	Control = BDL = DDC
119	noHit;	0.13937	0.193819	Control = BDL = DDC
120	Bacteria;Actinobacteriota;Actinobacteria;Corynebacteriales;Corynebacteriaceae;Lawsonella;?	0.140234	0.193819	Control = BDL = DDC
121	Bacteria;Actinobacteriota;Actinobacteria;Micrococcales;Micrococcaceae;Kocuria;?	0.140234	0.193819	Control = BDL = DDC
122	Bacteria;Actinobacteriota;Actinobacteria;Pseudonocardiales;Pseudonocardiaceae;?;?	0.140234	0.193819	Control = BDL = DDC
123	Bacteria;Bacteroidota;Bacteroidia;Bacteroidales;Rikenellaceae;Rikenellaceae RC9 gut group;?	0.140234	0.193819	Control = BDL = DDC
124	Bacteria;Firmicutes;Clostridia;Monoglobales;Monoglobaceae;Monoglobus;?	0.152784	0.209462	Control = BDL = DDC
125	Bacteria;Firmicutes;Clostridia;Oscillospirales;Oscillospiraceae;Oscillibacter;?	0.163916	0.222926	Control = BDL = DDC
126	Bacteria;Proteobacteria;Alphaproteobacteria;Rickettsiales;Mitochondria;?;?	0.168378	0.227177	DDC = Control = BDL
127	Bacteria;Firmicutes;Clostridia;Lachnospirales;Lachnospiraceae;Anaerostipes;?	0.195873	0.262192	Control = BDL = DDC
128	Bacteria;Bacteroidota;Bacteroidia;Bacteroidales;Tannerellaceae;?;?	0.200045	0.265685	NA
129	Bacteria;Firmicutes;Bacilli;Lactobacillales;Carnobacteriaceae;Atopostipes;Firmicutes oral	0.209486	0.276067	NA

130	Bacteria;Firmicutes;Clostridia;Lachnospirales;Lachnospiraceae;A2;?	0.232094	0.303508	NA
131	Bacteria;Bacteroidota;Bacteroidia;Bacteroidales;Rikenellaceae;Alistipes;uncultured Bacteroidales bacterium	0.24809	0.321949	NA
132	Bacteria;Actinobacteriota;Actinobacteria;Micrococcales;Micrococcaceae;?;?	0.259449	0.334139	NA
133	Bacteria;Actinobacteriota;Coriobacteriia;Coriobacteriales;?;?;?	0.261827	0.334666	NA
134	Bacteria;Cyanobacteria;Cyanobacteriia;Chloroplast;?;?;?	0.275046	0.34894	NA
135	Bacteria;Firmicutes;Bacilli;Staphylococcales;Staphylococcaceae;Staphylococcus;?	0.29903	0.376557	NA
136	Bacteria;Actinobacteriota;Coriobacteriia;Coriobacteriales;Eggerthellaceae;Enterorhabdus;?	0.321123	0.391425	NA
137	Bacteria;Proteobacteria;Gammaproteobacteria;Burkholderiales;Burkholderiaceae;Burkholderia-Caballeronia-Paraburkholderia;Paraburkholderia ferrariae	0.324652	0.391425	NA
138	Bacteria;Proteobacteria;Gammaproteobacteria;Burkholderiales;Burkholderiaceae;Burkholderia-Caballeronia-Paraburkholderia;Paraburkholderia susongensis	0.324652	0.391425	NA
139	Bacteria;Proteobacteria;Gammaproteobacteria;Burkholderiales;Burkholderiaceae;Ralstonia;?	0.324652	0.391425	NA
140	Bacteria;Proteobacteria;Gammaproteobacteria;Burkholderiales;Comamonadaceae;?;?	0.324652	0.391425	NA
141	Bacteria;Proteobacteria;Gammaproteobacteria;Burkholderiales;Comamonadaceae;Pelomonas;Pelomonas puraquae	0.324652	0.391425	NA
142	Bacteria;Firmicutes;Bacilli;RF39;?;?;?	0.343607	0.41136	NA
143	Bacteria;Firmicutes;Clostridia;Oscillospirales;Ruminococcaceae;Faecalibacterium;?	0.367384	0.426531	NA
144	Bacteria;Desulfobacterota;Desulfovibrionia;Desulfovibrionales;Desulfovibrionaceae;Bilophila;?	0.374009	0.426531	NA
145	Bacteria;Firmicutes;Clostridia;Peptostreptococcales-Tissierellales;Peptostreptococcaceae;?;?	0.374586	0.426531	NA
146	Bacteria;Actinobacteriota;Coriobacteriia;Coriobacteriales;Coriobacteriaceae;Collinsella;?	0.388896	0.426531	NA
147	Bacteria;Bacteroidota;Bacteroidia;Bacteroidales;Marinifilaceae;Odoribacter;?	0.388896	0.426531	NA
148	Bacteria;Bacteroidota;Bacteroidia;Bacteroidales;Rikenellaceae;Alistipes;Alistipes finegoldii	0.388896	0.426531	NA
149	Bacteria;Bacteroidota;Bacteroidia;Bacteroidales;Tannerellaceae;Parabacteroides;Parabacteroides merdae	0.388896	0.426531	NA
150	Bacteria;Cyanobacteria;Vampirivibrionia;Gastranaerophilales;gut metagenome;?;?	0.388896	0.426531	NA
151	Bacteria;Firmicutes;Clostridia;Christensenellales;Christensenellaceae;Christensenella sp. Marseille-P2437;?	0.388896	0.426531	NA
152	Bacteria;Firmicutes;Clostridia;Lachnospirales;Lachnospiraceae;Marvinbryantia;?	0.388896	0.426531	NA
153	Bacteria;Firmicutes;Clostridia;Oscillospirales;[Eubacterium] coprostanoligenes group;gut metagenome;?	0.388896	0.426531	NA
154	Bacteria;Proteobacteria;Alphaproteobacteria;Rhizobiales;Beijerinckiaceae;Methylobacterium-Methylorubrum;?	0.388896	0.426531	NA
155	Bacteria;Proteobacteria;Gammaproteobacteria;Pseudomonadales;?;?;?	0.388896	0.426531	NA
156	Bacteria;Firmicutes;Bacilli;Lactobacillales;Enterococcaceae;Enterococcus;?	0.403354	0.439552	NA
157	Bacteria;Bacteroidota;Bacteroidia;Bacteroidales;Muribaculaceae;uncultured Bacteroidales bacterium;?	0.437373	0.473589	NA
158	Bacteria;?;?;?;?;?;?	0.454436	0.48895	NA
159	Bacteria;Proteobacteria;Gammaproteobacteria;Burkholderiales;Sutterellaceae;Parasutterella;Turicimonas muris	0.459506	0.491296	NA
160	Bacteria;Bacteroidota;Bacteroidia;Bacteroidales;Prevotellaceae;?;?	0.476534	0.506318	NA
161	Bacteria;Proteobacteria;Gammaproteobacteria;Burkholderiales;Sutterellaceae;Parasutterella;?	0.491938	0.519438	NA
162	Bacteria;Bacteroidota;Bacteroidia;Bacteroidales;Bacteroidaceae;Bacteroides;?	0.497754	0.522334	NA

163	Bacteria;Firmicutes;Bacilli;Lactobacillales;Lactobacillaceae;HT002;Lactobacillus sp.	0.507641	0.529442	NA
164	Bacteria;Desulfobacterota;Desulfovibrionia;Desulfovibrionales;Desulfovibrionaceae;?;?	0.519055	0.538045	NA
165	Bacteria;Firmicutes;Clostridia;Peptostreptococcales-Tissierellales;?;?;?	0.574681	0.592095	NA
166	Bacteria;Actinobacteriota;Coriobacteriia;Coriobacteriales;Eggerthellaceae;Parvibacter;?	0.628484	0.643628	NA
167	Bacteria;Firmicutes;Clostridia;Lachnospirales;Lachnospiraceae;?;?	0.767239	0.781021	NA
168	Bacteria;Bacteroidota;Bacteroidia;Bacteroidales;Rikenellaceae;Alistipes;?	0.844726	0.850581	NA
169	Bacteria;Verrucomicrobiota;Verrucomicrobiae;Verrucomicrobiales;Akkermansiaceae;Akkermansia;?	0.845578	0.850581	NA
170	Bacteria;Firmicutes;Bacilli;?;?;?;?	0.853157	0.853157	NA

Table S2. Bacterial taxa after 16s rRNA amplicon sequencing of mouse faecal samples. 16s rRNA sequencing of faecal samples from control, BDL and 0.1%DDC diet fed mice (7days). Analyses were done from n=5-7 mice. (Ctrl vs BDL vs).

Table S3. Statistical results of enrichment over the taxonomic levels of phylum to species.

Phylum

Rank	Feature name	p-value	q-value	Direction
1	Bacteria;Cyanobacteria	0.000193	0.00232	BDL+ABT2 = BDL+ABT>> BDL = Control
2	Bacteria;Firmicutes	0.000764	0.004487	Control>> BDL>> BDL+ABT = BDL+ABT2
3	Bacteria;Actinobacteriota	0.001122	0.004487	BDL+ABT = BDL+ABT2>= BDL = Control
4	Bacteria;Patescibacteria	0.002231	0.006003	BDL>> BDL+ABT = BDL+ABT2 = Control
5	Bacteria;?	0.002501	0.006003	Control > BDL = BDL+ABT2 = BDL+ABT
6	Bacteria;Bacteroidota	0.003846	0.007691	BDL+ABT = BDL+ABT2 = BDL>= Control
7	Bacteria;Deferribacterota	0.006648	0.011396	Control>= BDL = BDL+ABT = BDL+ABT2
8	Bacteria;Desulfobacterota	0.011032	0.016548	BDL+ABT2 = Control = BDL = BDL+ABT
9	Bacteria;Verrucomicrobiota	0.026843	0.035791	Control = BDL+ABT = BDL+ABT2 = BDL
10	Bacteria;Proteobacteria	0.048527	0.057753	BDL+ABT = BDL+ABT2 = Control = BDL
11	noHit;	0.052941	0.057753	Control = BDL = BDL+ABT = BDL+ABT2
12	Bacteria;Campylobacterota	0.114862	0.114862	Control = BDL = BDL+ABT = BDL+ABT2

Class

Rank	Feature name	p-value	q-value	Direction
1	Bacteria;Cyanobacteria;Vampirivibrionia	0.000193	0.003481	BDL+ABT2 = BDL+ABT>> BDL = Control
2	Bacteria;Firmicutes;Bacilli	0.000789	0.006526	Control = BDL>> BDL+ABT = BDL+ABT2
3	Bacteria;Actinobacteriota;Coriobacteriia	0.001088	0.006526	BDL+ABT = BDL+ABT2 > BDL = Control
4	Bacteria;Patescibacteria;Saccharimonadia	0.002231	0.009004	BDL>> BDL+ABT = BDL+ABT2 = Control
5	Bacteria;?;?	0.002501	0.009004	Control > BDL = BDL+ABT2 = BDL+ABT
6	Bacteria;Bacteroidota;Bacteroidia	0.003846	0.011537	BDL+ABT = BDL+ABT2 = BDL>= Control
7	Bacteria;Proteobacteria;Alphaproteobacteria	0.005816	0.014954	BDL+ABT > Control>= BDL+ABT2 = BDL
8	Bacteria;Deferribacterota;Deferribacteres	0.006648	0.014957	Control>= BDL = BDL+ABT = BDL+ABT2
9	Bacteria;Desulfobacterota;Desulfovibrionia	0.011032	0.01973	BDL+ABT2 = Control = BDL = BDL+ABT
10	Bacteria;Actinobacteriota;Actinobacteria	0.011838	0.01973	BDL = BDL+ABT2 = BDL+ABT>= Control
11	Bacteria;Actinobacteriota;?	0.012258	0.01973	Control = BDL+ABT2 = BDL+ABT = BDL
12	Bacteria;Firmicutes;Clostridia	0.013153	0.01973	Control = BDL+ABT2 = BDL+ABT = BDL
13	Bacteria;Firmicutes;?	0.024649	0.03413	Control = BDL+ABT2 = BDL = BDL+ABT
14	Bacteria;Verrucomicrobiota;Verrucomicrobiae	0.026843	0.034513	Control = BDL+ABT = BDL+ABT2 = BDL
15	noHit;	0.052941	0.063529	Control = BDL = BDL+ABT = BDL+ABT2
16	Bacteria;Campylobacterota;Campylobacteria	0.114862	0.12922	Control = BDL = BDL+ABT = BDL+ABT2
17	Bacteria;Cyanobacteria;Cyanobacteriia	0.261923	0.277331	NA
18	Bacteria;Proteobacteria;Gammaproteobacteria	0.959641	0.959641	NA

Order

Rank	Feature name	p-value	q-value	Direction
1	Bacteria;Firmicutes;Clostridia;Clostridia UCG-014	3.82E-05	0.001179	BDL>> BDL+ABT2 > BDL+ABT>> Control
2	Bacteria;Bacteroidota;Bacteroidia;?	0.000106	0.001179	Control>> BDL = BDL+ABT = BDL+ABT2
3	Bacteria;Firmicutes;Bacilli;Erysipelotrichales	0.000166	0.001179	Control>> BDL+ABT2>= BDL = BDL+ABT
4	Bacteria;Firmicutes;Clostridia;Peptococcales	0.000185	0.001179	BDL+ABT2>= BDL+ABT = BDL>> Control
5	Bacteria;Cyanobacteria;Vampirivibrionia;Gastranaerophilales	0.000193	0.001179	BDL+ABT2 = BDL+ABT>> BDL = Control
6	Bacteria;Firmicutes;Clostridia;Clostridia vadinBB60 group	0.000208	0.001179	Control>> BDL = BDL+ABT = BDL+ABT2

7	Bacteria;Firmicutes;Bacilli;Acholeplasmatales	0.000223	0.001179	BDL>> Control = BDL+ABT = BDL+ABT2
8	Bacteria;Firmicutes;Clostridia;Christensenellales	0.000608	0.002778	BDL = BDL+ABT2 = BDL+ABT>> Control
9	Bacteria;Firmicutes;Clostridia;Lachnospirales	0.000676	0.002778	Control>> BDL+ABT = BDL = BDL+ABT2
10	Bacteria;Firmicutes;Clostridia;Clostridiales	0.001044	0.003658	BDL = BDL+ABT2 = BDL+ABT>> Control
11	Bacteria;Actinobacteriota;Coriobacteriia;Coriobacteriales	0.001088	0.003658	BDL+ABT = BDL+ABT2 > BDL = Control
12	Bacteria;Patescibacteria;Saccharimonadia;Saccharimonadales	0.002231	0.006879	BDL>> BDL+ABT = BDL+ABT2 = Control
13	Bacteria;?;?;?	0.002501	0.007119	Control > BDL = BDL+ABT2 = BDL+ABT
14	Bacteria;Proteobacteria;Gammaproteobacteria;Pseudomonadales	0.002707	0.007155	BDL = BDL+ABT2 > Control = BDL+ABT
15	Bacteria;Actinobacteriota;Actinobacteria;Corynebacteriales	0.003175	0.007831	BDL = BDL+ABT2 = BDL+ABT > Control
16	Bacteria;Proteobacteria;Gammaproteobacteria;Burkholderiales	0.003428	0.007927	Control>= BDL = BDL+ABT2>= BDL+ABT
17	Bacteria;Bacteroidota;Bacteroidia;Bacteroidales	0.003846	0.00837	BDL+ABT = BDL+ABT2 = BDL>= Control
18	Bacteria;Firmicutes;Bacilli;?	0.004441	0.009129	Control = BDL = BDL+ABT = BDL+ABT2
19	Bacteria;Proteobacteria;Alphaproteobacteria;Rhodospirillales	0.004853	0.009451	BDL+ABT > Control>= BDL+ABT2 = BDL
20	Bacteria;Proteobacteria;Gammaproteobacteria;Enterobacterales	0.005357	0.009911	BDL+ABT2 = BDL+ABT = BDL > Control
21	Bacteria;Firmicutes;Bacilli;Lactobacillales	0.005724	0.010086	BDL>> Control = BDL+ABT = BDL+ABT2
22	Bacteria;Deferribacterota;Deferribacteres;Deferribacterales	0.006648	0.01118	Control>= BDL = BDL+ABT = BDL+ABT2
23	Bacteria;Desulfobacterota;Desulfovibrionia;Desulfovibrionales	0.011032	0.017747	BDL+ABT2 = Control = BDL = BDL+ABT
24	Bacteria;Actinobacteriota;?;?	0.012258	0.018898	Control = BDL+ABT2 = BDL+ABT = BDL
25	Bacteria;Firmicutes;Clostridia;Oscillospirales	0.017023	0.025193	BDL+ABT2 = BDL = BDL+ABT = Control
26	Bacteria;Firmicutes;?;?	0.024649	0.035078	Control = BDL+ABT2 = BDL = BDL+ABT
27	Bacteria;Verrucomicrobiota;Verrucomicrobiae;Verrucomicrobiales	0.026843	0.036785	Control = BDL+ABT = BDL+ABT2 = BDL
28	Bacteria;Firmicutes;Bacilli;Staphylococcales	0.049681	0.06565	BDL>= Control = BDL+ABT2 = BDL+ABT
29	noHit;	0.052941	0.067545	Control = BDL = BDL+ABT = BDL+ABT2
30	Bacteria;Campylobacterota;Campylobacteria;Campylobacterales	0.114862	0.141664	Control = BDL = BDL+ABT = BDL+ABT2
31	Bacteria;Firmicutes;Clostridia;?	0.139614	0.166636	BDL+ABT2 = Control = BDL+ABT = BDL
32	Bacteria;Actinobacteriota;Actinobacteria;Propionibacteriales	0.176793	0.204416	Control = BDL = BDL+ABT = BDL+ABT2
33	Bacteria;Proteobacteria;Alphaproteobacteria;Rickettsiales	0.199219	0.219287	BDL+ABT = Control = BDL = BDL+ABT2
34	Bacteria;Firmicutes;Bacilli;RF39	0.201507	0.219287	NA
35	Bacteria;Cyanobacteria;Cyanobacteriia;Chloroplast	0.261923	0.27689	NA
36	Bacteria;Actinobacteriota;Actinobacteria;Micrococcales	0.365584	0.375739	NA
37	Bacteria;Firmicutes;Clostridia;Peptostreptococcales-Tissierellales	0.686288	0.686288	NA

Family

Rank	Feature name	p-value	q-value	Direction
1	Bacteria;Firmicutes;Clostridia;Clostridia UCG-014;?	3.82E-05	0.001338	BDL>> BDL+ABT2 > BDL+ABT>> Control
2	Bacteria;Firmicutes;Clostridia;Clostridia vadinBB60 group;uncultured organism	7.40E-05	0.001338	Control>> BDL = BDL+ABT = BDL+ABT2
3	Bacteria;Proteobacteria;Gammaproteobacteria;Burkholderiales;?	9.91E-05	0.001338	Control>> BDL = BDL+ABT = BDL+ABT2
4	Bacteria;Bacteroidota;Bacteroidia;?;?	0.000106	0.001338	Control>> BDL = BDL+ABT = BDL+ABT2
5	Bacteria;Firmicutes;Clostridia;Clostridia vadinBB60 group;uncultured Clostridiales bacterium	0.000106	0.001338	Control>> BDL = BDL+ABT = BDL+ABT2
6	Bacteria;Bacteroidota;Bacteroidia;Bacteroidales;?	0.000117	0.001338	BDL+ABT2 = BDL+ABT>> BDL>> Control
7	Bacteria;Firmicutes;Bacilli;Erysipelotrichales;Erysipelotrichaceae	0.000149	0.001338	Control>> BDL+ABT2 = BDL = BDL+ABT
8	Bacteria;Bacteroidota;Bacteroidia;Bacteroidales;Tannerellaceae	0.000169	0.001338	BDL+ABT2 = BDL+ABT>> Control>> BDL
9	Bacteria;Firmicutes;Clostridia;Peptococcales;Peptococcaceae	0.000185	0.001338	BDL+ABT2>= BDL+ABT = BDL>> Control
10	Bacteria;Cyanobacteria;Vampirivibrionia;Gastranaerophilales;?	0.000193	0.001338	BDL+ABT2 = BDL+ABT>> BDL = Control
11	Bacteria;Firmicutes;Clostridia;Clostridia vadinBB60 group;?	0.000208	0.001338	Control>> BDL = BDL+ABT = BDL+ABT2
12	Bacteria;Firmicutes;Bacilli;Acholeplasmatales;Acholeplasmataceae	0.000223	0.001338	BDL>> Control = BDL+ABT = BDL+ABT2
13	Bacteria;Firmicutes;Clostridia;Oscillospirales;Butyricicoccaceae	0.000361	0.001999	BDL = BDL+ABT2 = BDL+ABT>> Control
14	Bacteria;Firmicutes;Clostridia;Oscillospirales;[Eubacterium] coprostanoligenes group	0.000404	0.002079	BDL>> Control = BDL+ABT = BDL+ABT2
15	Bacteria;Firmicutes;Clostridia;Lachnospirales;Lachnospiraceae	0.000606	0.002815	Control>> BDL+ABT = BDL = BDL+ABT2
16	Bacteria;Firmicutes;Clostridia;Christensenellales;Christensenellaceae	0.000626	0.002815	BDL = BDL+ABT2 = BDL+ABT>> Control
17	Bacteria;Actinobacteriota;Coriobacteriia;Coriobacteriales;Eggerthellaceae	0.000874	0.003701	BDL+ABT = BDL+ABT2 > BDL>= Control
18	Bacteria;Firmicutes;Clostridia;Clostridiales;Clostridiaceae	0.001044	0.004178	BDL = BDL+ABT2 = BDL+ABT>> Control
19	Bacteria;Bacteroidota;Bacteroidia;Bacteroidales;Marinifilaceae	0.001117	0.004234	BDL+ABT = BDL = BDL+ABT2>> Control
20	Bacteria;Bacteroidota;Bacteroidia;Bacteroidales;Bacteroidaceae	0.002074	0.007328	BDL+ABT = BDL+ABT2 > Control = BDL
21	Bacteria;Patescibacteria;Saccharimonadia;Saccharimonadales;Saccharimonadaceae	0.002231	0.007328	BDL>> BDL+ABT = BDL+ABT2 = Control
22	Bacteria;Firmicutes;Clostridia;Oscillospirales;Oscillospiraceae	0.002239	0.007328	BDL+ABT2>= BDL+ABT = BDL > Control
23	Bacteria;?;?;?;?	0.002501	0.00783	Control > BDL = BDL+ABT2 = BDL+ABT

24	Bacteria;Actinobacteriota;Actinobacteria;Corynebacteriales;Corynebacteriaceae	0.003175	0.009525	BDL = BDL+ABT2 = BDL+ABT > Control
25	Bacteria;Proteobacteria;Gammaproteobacteria;Burkholderiales;Sutterellaceae	0.003428	0.009872	Control>= BDL = BDL+ABT2>= BDL+ABT
26	Bacteria;Firmicutes;Bacilli;Lactobacillales;Lactobacillaceae	0.003825	0.010139	BDL>> Control = BDL+ABT2 = BDL+ABT
27	Bacteria;Firmicutes;Clostridia;Lachnospirales;?	0.003861	0.010139	Control > BDL+ABT = BDL+ABT2 = BDL
28	Bacteria;Cyanobacteria;Vampirivibrionia;Gastranaerophilales;uncultured rumen bacterium	0.003943	0.010139	BDL+ABT = BDL+ABT2 = BDL>= Control
29	Bacteria;Firmicutes;Bacilli;?;?	0.004441	0.011026	Control = BDL = BDL+ABT = BDL+ABT2
30	Bacteria;Proteobacteria;Alphaproteobacteria;Rhodospirillales;?	0.004853	0.011648	BDL+ABT > Control>= BDL+ABT2 = BDL
31	Bacteria;Proteobacteria;Gammaproteobacteria;Enterobacterales;Enterobacteriaceae	0.005357	0.012442	BDL+ABT2 = BDL+ABT = BDL > Control
32	Bacteria;Deferribacterota;Deferribacteres;Deferribacterales;Deferribacteraceae	0.006648	0.014957	Control>= BDL = BDL+ABT = BDL+ABT2
33	Bacteria;Proteobacteria;Gammaproteobacteria;Enterobacterales;Pasteurellaceae	0.007875	0.017181	BDL+ABT>= Control = BDL = BDL+ABT2
34	Bacteria;Proteobacteria;Gammaproteobacteria;Pseudomonadales;?	0.009973	0.021119	BDL = BDL+ABT2>= Control = BDL+ABT
35	Bacteria;Desulfobacterota;Desulfovibrionia;Desulfovibrionales;Desulfovibrionaceae	0.011032	0.022525	BDL+ABT2 = Control = BDL = BDL+ABT
36	Bacteria;Firmicutes;Clostridia;Oscillospirales;?	0.011575	0.022525	BDL+ABT2 = BDL+ABT = BDL = Control
37	Bacteria;Firmicutes;Clostridia;Christensenellales;?	0.011575	0.022525	BDL+ABT2 > Control = BDL = BDL+ABT
38	Bacteria;Firmicutes;Bacilli;Lactobacillales;Streptococcaceae	0.012004	0.022631	BDL+ABT = BDL+ABT2 = BDL>= Control
39	Bacteria;Actinobacteriota;?;?;?	0.012258	0.022631	Control = BDL+ABT2 = BDL+ABT = BDL
40	Bacteria;Actinobacteriota;Coriobacteriia;Coriobacteriales;Atopobiaceae	0.01791	0.032237	Control = BDL+ABT = BDL+ABT2 = BDL
41	Bacteria;Firmicutes;Bacilli;Lactobacillales;Enterococcaceae	0.022403	0.039342	BDL+ABT = BDL+ABT2 = BDL = Control
42	Bacteria;Verrucomicrobiota;Verrucomicrobiae;Verrucomicrobiales;Akkermansiaceae	0.023823	0.040034	Control = BDL+ABT = BDL+ABT2 = BDL
43	Bacteria;Bacteroidota;Bacteroidia;Bacteroidales;Muribaculaceae	0.023909	0.040034	BDL>= BDL+ABT = BDL+ABT2 = Control
44	Bacteria;Firmicutes;?;?;?	0.024649	0.040335	Control = BDL+ABT2 = BDL = BDL+ABT
45	Bacteria;Firmicutes;Clostridia;Oscillospirales;Ruminococcaceae	0.028171	0.045073	Control = BDL+ABT2 = BDL = BDL+ABT
46	Bacteria;Cyanobacteria;Vampirivibrionia;Gastranaerophilales;gut metagenome	0.036891	0.056906	BDL+ABT2 = Control = BDL = BDL+ABT
47	Bacteria;Actinobacteriota;Coriobacteriia;Coriobacteriales;?	0.037147	0.056906	Control = BDL = BDL+ABT = BDL+ABT2

48	Bacteria;Firmicutes;Bacilli;Staphylococcales;Staphylococcaceae	0.049681	0.074521	BDL>= Control = BDL+ABT2 = BDL+ABT
49	noHit;	0.052941	0.07779	Control = BDL = BDL+ABT = BDL+ABT2
50	Bacteria;Firmicutes;Bacilli;Erysipelotrichales;Erysipelatoclostridiaceae	0.061002	0.087843	BDL+ABT2 = BDL = BDL+ABT = Control
51	Bacteria;Firmicutes;Bacilli;Lactobacillales;?	0.103811	0.146557	BDL+ABT = BDL+ABT2 = Control = BDL
52	Bacteria;Campylobacterota;Campylobacteria;Campylobacterales;Helicobacteraceae	0.114862	0.15904	Control = BDL = BDL+ABT = BDL+ABT2
53	Bacteria;Verrucomicrobiota;Verrucomicrobiae;Verrucomicrobiales;?	0.124835	0.169587	BDL = Control = BDL+ABT2 = BDL+ABT
54	Bacteria;Firmicutes;Clostridia;?;?	0.139614	0.186152	BDL+ABT2 = Control = BDL+ABT = BDL
55	Bacteria;Proteobacteria;Gammaproteobacteria;Burkholderiales;Burkholderiaceae	0.174122	0.221188	Control = BDL = BDL+ABT = BDL+ABT2
56	Bacteria;Actinobacteriota;Actinobacteria;Propionibacteriales;Propionibacteriaceae	0.176793	0.221188	Control = BDL = BDL+ABT = BDL+ABT2
57	Bacteria;Proteobacteria;Gammaproteobacteria;Burkholderiales;Comamonadaceae	0.176793	0.221188	Control = BDL = BDL+ABT = BDL+ABT2
58	Bacteria;Firmicutes;Clostridia;Oscillospirales;UCG-010	0.178179	0.221188	Control = BDL = BDL+ABT = BDL+ABT2
59	Bacteria;Proteobacteria;Alphaproteobacteria;Rickettsiales;Mitochondria	0.199219	0.241809	BDL+ABT = Control = BDL = BDL+ABT2
60	Bacteria;Firmicutes;Bacilli;RF39;?	0.201507	0.241809	NA
61	Bacteria;Firmicutes;Bacilli;Lactobacillales;Aerococcaceae	0.209798	0.247631	NA
62	Bacteria;Proteobacteria;Gammaproteobacteria;Enterobacterales;?	0.237299	0.275573	NA
63	Bacteria;Cyanobacteria;Cyanobacteriia;Chloroplast;?	0.261923	0.295931	NA
64	Bacteria;Bacteroidota;Bacteroidia;Bacteroidales;Prevotellaceae	0.26305	0.295931	NA
65	Bacteria;Bacteroidota;Bacteroidia;Bacteroidales;Rikenellaceae	0.304428	0.337213	NA
66	Bacteria;Firmicutes;Bacilli;RF39;uncultured Firmicutes bacterium	0.320762	0.3447	NA
67	Bacteria;Proteobacteria;Gammaproteobacteria;Pseudomonadales;Moraxellaceae	0.320762	0.3447	NA
68	Bacteria;Firmicutes;Bacilli;Lactobacillales;Carnobacteriaceae	0.338174	0.358066	NA
69	Bacteria;Firmicutes;Clostridia;Peptostreptococcales-Tissierellales;Anaerovoracaceae	0.360946	0.373884	NA
70	Bacteria;Actinobacteriota;Actinobacteria;Micrococcales;Micrococcaceae	0.365584	0.373884	NA
71	Bacteria;Firmicutes;Clostridia;Clostridiales;?	0.368691	0.373884	NA
72	Bacteria;Firmicutes;Clostridia;Peptostreptococcales-Tissierellales;?	0.61084	0.61084	NA

Genus

Rank	Feature name	p-value	q-value	Direction

1	Bacteria;Firmicutes;Clostridia;Clostridia UCG-014;?;?	3.82E-05	0.001449	BDL>> BDL+ABT2 > BDL+ABT>> Control
2	Bacteria;Firmicutes;Bacilli;Erysipelotrichales;Erysipelotrichaceae;?	4.21E-05	0.001449	Control>> BDL>> BDL+ABT2>= BDL+ABT
3	Bacteria;Actinobacteriota;Coriobacteriia;Coriobacteriales;Eggerthellaceae;Parvibacter	4.75E-05	0.001449	BDL+ABT2 = BDL+ABT>> BDL>> Control
4	Bacteria;Bacteroidota;Bacteroidia;Bacteroidales;Muribaculaceae;uncultured Muribaculaceae bacterium	5.03E-05	0.001449	BDL > BDL+ABT>= BDL+ABT2>> Control
5	Bacteria;Firmicutes;Clostridia;Clostridia vadinBB60 group;uncultured organism;?	7.40E-05	0.001449	Control>> BDL = BDL+ABT = BDL+ABT2
6	Bacteria;Firmicutes;Clostridia;Oscillospirales;Oscillospiraceae;?	9.01E-05	0.001449	BDL>= BDL+ABT2 > BDL+ABT>> Control
7	Bacteria;Proteobacteria;Gammaproteobacteria;Burkholderiales;?;?	9.91E-05	0.001449	Control>> BDL = BDL+ABT = BDL+ABT2
8	Bacteria;Firmicutes;Clostridia;Oscillospirales;Oscillospiraceae;Oscillibacter	0.000101	0.001449	BDL+ABT2 = BDL+ABT = BDL>> Control
9	Bacteria;Bacteroidota;Bacteroidia;?;?;?	0.000106	0.001449	Control>> BDL = BDL+ABT = BDL+ABT2
10	Bacteria;Firmicutes;Clostridia;Clostridia vadinBB60 group;uncultured Clostridiales bacterium;?	0.000106	0.001449	Control>> BDL = BDL+ABT = BDL+ABT2
11	Bacteria;Bacteroidota;Bacteroidia;Bacteroidales;?;?	0.000117	0.001456	BDL+ABT2 = BDL+ABT>> BDL>> Control
12	Bacteria;Bacteroidota;Bacteroidia;Bacteroidales;Tannerellaceae;Parabacteroides	0.000169	0.001583	BDL+ABT2 = BDL+ABT>> Control>> BDL
13	Bacteria;Firmicutes;Clostridia;Peptococcales;Peptococcaceae;?	0.000171	0.001583	BDL+ABT2>= BDL+ABT = BDL>> Control
14	Bacteria;Patescibacteria;Saccharimonadia;Saccharimonadales;Saccharimonadaceae;Candidatus Saccharimonas	0.000191	0.001583	BDL>> BDL+ABT = BDL+ABT2>> Control
15	Bacteria;Cyanobacteria;Vampirivibrionia;Gastranaerophilales;?;?	0.000193	0.001583	BDL+ABT2 = BDL+ABT>> BDL = Control
16	Bacteria;Firmicutes;Clostridia;Clostridia vadinBB60 group;?;?	0.000208	0.001583	Control>> BDL = BDL+ABT = BDL+ABT2
17	Bacteria;Bacteroidota;Bacteroidia;Bacteroidales;Bacteroidaceae;Bacteroides	0.000213	0.001583	BDL+ABT2 = BDL+ABT>> BDL = Control
18	Bacteria;Firmicutes;Bacilli;Erysipelotrichales;Erysipelotrichaceae;Faecalibaculum	0.000219	0.001583	Control>> BDL+ABT2 > BDL+ABT = BDL
19	Bacteria;Firmicutes;Bacilli;Acholeplasmatales;Acholeplasmataceae;Anaeroplasma	0.000223	0.001583	BDL>> Control = BDL+ABT = BDL+ABT2
20	Bacteria;Firmicutes;Clostridia;Lachnospirales;Lachnospiraceae;Roseburia	0.000248	0.001583	Control>> BDL+ABT2 = BDL>= BDL+ABT
21	Bacteria;Firmicutes;Clostridia;Oscillospirales;Oscillospiraceae;NK4A214 group	0.000259	0.001583	BDL>> Control = BDL+ABT = BDL+ABT2
22	Bacteria;Deferribacterota;Deferribacteres;Deferribacterales;Deferribacteraceae;?	0.000259	0.001583	Control > BDL = BDL+ABT = BDL+ABT2

23	Bacteria;Firmicutes;Bacilli;Lactobacillales;Lactobacillaceae;Lactobacillus	0.0002 74	0.0015 83	BDL = Control>> BDL+ABT = BDL+ABT2
24	Bacteria;Verrucomicrobiota;Verrucomicrobiae;Verrucomicrobiales;Akkermansi aceae;Akkermansia	0.0002 77	0.0015 83	Control>> BDL = BDL+ABT = BDL+ABT2
25	Bacteria;Bacteroidota;Bacteroidia;Bacteroidales;Marinifilaceae;?	0.0003 74	0.0020 09	BDL+ABT = BDL+ABT2>= BDL > Control
26	Bacteria;Firmicutes;Clostridia;Lachnospirales;Lachnospiraceae;Lachnospiracea e FCS020 group	0.0004 02	0.0020 09	Control>> BDL+ABT2 = BDL > BDL+ABT
27	Bacteria;Firmicutes;Clostridia;Oscillospirales;[Eubacterium] coprostanoligenes group;gut metagenome	0.0004 04	0.0020 09	BDL>> Control = BDL+ABT = BDL+ABT2
28	Bacteria;Bacteroidota;Bacteroidia;Bacteroidales;Rikenellaceae;Rikenella	0.0004 26	0.0020 09	BDL+ABT = BDL+ABT2 = BDL>> Control
29	Bacteria;Bacteroidota;Bacteroidia;Bacteroidales;Muribaculaceae;mouse gut metagenome	0.0004 28	0.0020 09	Control>= BDL = BDL+ABT > BDL+ABT2
30	Bacteria;Firmicutes;Clostridia;Lachnospirales;Lachnospiraceae;Blautia	0.0004 4	0.0020 09	BDL+ABT2 = BDL = BDL+ABT>> Control
31	Bacteria;Bacteroidota;Bacteroidia;Bacteroidales;Prevotellaceae;?	0.0004 57	0.0020 18	BDL = BDL+ABT = BDL+ABT2>> Control
32	Bacteria;Firmicutes;Bacilli;Lactobacillales;Lactobacillaceae;Limosilactobacillus	0.0004 71	0.0020 18	BDL>= Control > BDL+ABT>= BDL+ABT2
33	Bacteria;Firmicutes;Clostridia;Lachnospirales;Lachnospiraceae;?	0.0005 66	0.0023 48	Control>> BDL+ABT = BDL = BDL+ABT2
34	Bacteria;Firmicutes;Clostridia;Lachnospirales;Lachnospiraceae;Lachnoclostridiu m	0.0006 48	0.0025 61	Control>> BDL+ABT2 = BDL = BDL+ABT
35	Bacteria;Firmicutes;Clostridia;Oscillospirales;Butyricicoccaceae;?	0.0006 54	0.0025 61	BDL = BDL+ABT2 = BDL+ABT>> Control
36	Bacteria;Bacteroidota;Bacteroidia;Bacteroidales;Rikenellaceae;?	0.0008 06	0.0029 94	BDL>= BDL+ABT>= BDL+ABT2 = Control
37	Bacteria;Proteobacteria;Gammaproteobacteria;Burkholderiales;Sutterellaceae; ?	0.0008 16	0.0029 94	Control = BDL+ABT>= BDL = BDL+ABT2
38	Bacteria;Firmicutes;Clostridia;Peptostreptococcales-Tissierellales;Anaerovoracaceae;[Eubacterium] brachy group	0.0008 3	0.0029 94	Control > BDL+ABT2 = BDL+ABT = BDL
39	Bacteria;Firmicutes;Clostridia;Clostridiales;Clostridiaceae;?	0.0010 44	0.0036 69	BDL = BDL+ABT2 = BDL+ABT>> Control
40	Bacteria;Bacteroidota;Bacteroidia;Bacteroidales;Muribaculaceae;uncultured organism	0.0011 3	0.0038 71	Control = BDL = BDL+ABT > BDL+ABT2
41	Bacteria;Bacteroidota;Bacteroidia;Bacteroidales;Rikenellaceae;Rikenellaceae RC9 gut group	0.0011 62	0.0038 81	BDL > Control = BDL+ABT = BDL+ABT2
42	Bacteria;Bacteroidota;Bacteroidia;Bacteroidales;Marinifilaceae;Odoribacter	0.0012 47	0.0040 69	BDL+ABT = BDL = BDL+ABT2>> Control
43	Bacteria;Actinobacteriota;Coriobacteriia;Coriobacteriales;Atopobiaceae;Olsene lla	0.0013 63	0.0043 43	Control = BDL+ABT = BDL+ABT2 = BDL

44	Bacteria;Firmicutes;Clostridia;Christensenellales;Christensenellaceae;?	0.001564	0.00477	BDL = BDL+ABT = BDL+ABT2>> Control
45	Bacteria;Firmicutes;Clostridia;Peptostreptococcales-Tissierellales;Anaerovoracaceae;?	0.001567	0.00477	BDL+ABT2 = BDL = BDL+ABT>> Control
46	Bacteria;Patescibacteria;Saccharimonadia;Saccharimonadales;Saccharimonadaceae;?	0.001697	0.005053	Control = BDL+ABT2 = BDL>= BDL+ABT
47	Bacteria;Actinobacteriota;Coriobacteriia;Coriobacteriales;Eggerthellaceae;Enterorhabdus	0.001924	0.005607	BDL+ABT = BDL+ABT2>= BDL = Control
48	Bacteria;Firmicutes;Clostridia;Oscillospirales;Ruminococcaceae;Incertae Sedis	0.002044	0.005835	Control>= BDL = BDL+ABT = BDL+ABT2
49	Bacteria;Firmicutes;Clostridia;Oscillospirales;Butyricicoccaceae;UCG-009	0.002233	0.006169	BDL+ABT2 = BDL = BDL+ABT>> Control
50	Bacteria;Bacteroidota;Bacteroidia;Bacteroidales;Muribaculaceae;?	0.002251	0.006169	BDL = BDL+ABT = BDL+ABT2 > Control
51	Bacteria;?;?;?;?;?	0.002501	0.006719	Control > BDL = BDL+ABT2 = BDL+ABT
52	Bacteria;Proteobacteria;Gammaproteobacteria;Enterobacterales;Enterobacteriaceae;?	0.002872	0.007567	BDL = BDL+ABT = BDL+ABT2>> Control
53	Bacteria;Desulfobacterota;Desulfovibrionia;Desulfovibrionales;Desulfovibrionaceae;?	0.003055	0.007754	Control = BDL+ABT2 = BDL = BDL+ABT
54	Bacteria;Firmicutes;Bacilli;Erysipelotrichales;Erysipelatoclostridiaceae;?	0.003064	0.007754	BDL+ABT = BDL+ABT2 = BDL>> Control
55	Bacteria;Firmicutes;Bacilli;Erysipelotrichales;Erysipelatoclostridiaceae;Candidatus Stoquefichus	0.00312	0.007754	Control > BDL = BDL+ABT = BDL+ABT2
56	Bacteria;Actinobacteriota;Actinobacteria;Corynebacteriales;Corynebacteriaceae;Corynebacterium	0.003175	0.007754	BDL = BDL+ABT2 = BDL+ABT > Control
57	Bacteria;Firmicutes;Clostridia;Christensenellales;Christensenellaceae;Christensenella sp. Marseille-P2437	0.003226	0.007754	BDL = BDL+ABT2 = BDL+ABT > Control
58	Bacteria;Proteobacteria;Gammaproteobacteria;Burkholderiales;Sutterellaceae;Parasutterella	0.003671	0.008671	Control = BDL+ABT2 = BDL > BDL+ABT
59	Bacteria;Firmicutes;Clostridia;Lachnospirales;?;?	0.003861	0.008966	Control > BDL+ABT = BDL+ABT2 = BDL
60	Bacteria;Cyanobacteria;Vampirivibrionia;Gastranaerophilales;uncultured rumen bacterium;?	0.003943	0.009003	BDL+ABT = BDL+ABT2 = BDL>= Control
61	Bacteria;Firmicutes;Bacilli;?;?;?	0.004441	0.009974	Control = BDL = BDL+ABT = BDL+ABT2
62	Bacteria;Proteobacteria;Alphaproteobacteria;Rhodospirillales;?;?	0.004853	0.010724	BDL+ABT > Control>= BDL+ABT2 = BDL
63	Bacteria;Actinobacteriota;Coriobacteriia;Coriobacteriales;Eggerthellaceae;DNF00809	0.005539	0.012046	BDL+ABT = BDL+ABT2 = BDL>= Control
64	Bacteria;Firmicutes;Clostridia;Lachnospirales;Lachnospiraceae;A2	0.00647	0.013849	BDL = BDL+ABT2 = BDL+ABT = Control
65	Bacteria;Deferribacterota;Deferribacteres;Deferribacterales;Deferribacteraceae;Mucispirillum	0.006648	0.014011	Control>= BDL = BDL+ABT = BDL+ABT2

66	Bacteria;Firmicutes;Clostridia;Lachnospirales;Lachnospiraceae;Lachnospiracea e NK4A136 group	0.0068 72	0.0142 65	BDL+ABT2 = BDL+ABT = BDL > Control
67	Bacteria;Proteobacteria;Gammaproteobacteria;Enterobacterales;Pasteurellace ae;?	0.0078 75	0.0161 02	BDL+ABT>= Control = BDL = BDL+ABT2
68	Bacteria;Proteobacteria;Gammaproteobacteria;Enterobacterales;Enterobacteri aceae;Escherichia-Shigella	0.0088 83	0.0178 96	BDL+ABT2 = BDL+ABT = BDL > Control
69	Bacteria;Proteobacteria;Gammaproteobacteria;Pseudomonadales;?;?	0.0099 73	0.0197 21	BDL = BDL+ABT2>= Control = BDL+ABT
70	Bacteria;Firmicutes;Bacilli;Acholeplasmatales;Acholeplasmataceae;?	0.0100 76	0.0197 21	BDL>= Control = BDL+ABT = BDL+ABT2
71	Bacteria;Firmicutes;Clostridia;Lachnospirales;Lachnospiraceae;[Eubacterium] xylanophilum group	0.0102 91	0.0198 58	BDL+ABT2 = BDL = Control = BDL+ABT
72	Bacteria;Firmicutes;Clostridia;Oscillospirales;?;?	0.0115 75	0.0217 24	BDL+ABT2 = BDL+ABT = BDL = Control
73	Bacteria;Firmicutes;Clostridia;Christensenellales;?;?	0.0115 75	0.0217 24	BDL+ABT2 > Control = BDL = BDL+ABT
74	Bacteria;Firmicutes;Bacilli;Lactobacillales;Streptococcaceae;Streptococcus	0.0120 04	0.0222 24	BDL+ABT = BDL+ABT2 = BDL>= Control
75	Bacteria;Actinobacteriota;?;?;?;?	0.0122 58	0.0223 92	Control = BDL+ABT2 = BDL+ABT = BDL
76	Bacteria;Firmicutes;Bacilli;Lactobacillales;Lactobacillaceae;HT002	0.0140 68	0.0253 4	BDL = Control = BDL+ABT = BDL+ABT2
77	Bacteria;Desulfobacterota;Desulfovibrionia;Desulfovibrionales;Desulfovibriona ceae;Desulfovibrio	0.0142 42	0.0253 4	BDL+ABT2 = Control = BDL = BDL+ABT
78	Bacteria;Firmicutes;Bacilli;Staphylococcales;Staphylococcaceae;Staphylococcus	0.0161 12	0.0282 98	BDL > Control = BDL+ABT = BDL+ABT2
79	Bacteria;Bacteroidota;Bacteroidia;Bacteroidales;Muribaculaceae;Muribaculum	0.0204 99	0.0355 48	Control = BDL+ABT = BDL = BDL+ABT2
80	Bacteria;Firmicutes;Clostridia;Lachnospirales;Lachnospiraceae;Anaerostipes	0.0220 15	0.0377 02	Control = BDL = BDL+ABT = BDL+ABT2
81	Bacteria;Firmicutes;Bacilli;Lactobacillales;Enterococcaceae;Enterococcus	0.0224 03	0.0378 91	BDL+ABT = BDL+ABT2 = BDL = Control
82	Bacteria;Firmicutes;Clostridia;Oscillospirales;Ruminococcaceae;Harryflintia	0.0227 53	0.0380 15	BDL+ABT2 = BDL+ABT>= Control = BDL
83	Bacteria;Firmicutes;?;?;?;?	0.0246 49	0.0406 86	Control = BDL+ABT2 = BDL = BDL+ABT
84	Bacteria;Firmicutes;Clostridia;Oscillospirales;Oscillospiraceae;UCG-005	0.0276 34	0.0450 7	BDL+ABT2 > Control = BDL = BDL+ABT
85	Bacteria;Cyanobacteria;Vampirivibrionia;Gastranaerophilales;gut metagenome;?	0.0368 91	0.0591 76	BDL+ABT2 = Control = BDL = BDL+ABT
86	Bacteria;Actinobacteriota;Coriobacteriia;Coriobacteriales;?;?	0.0371 47	0.0591 76	Control = BDL = BDL+ABT = BDL+ABT2

87	Bacteria;Firmicutes;Clostridia;Oscillospirales;Ruminococcaceae;Anaerotruncus	0.051981	0.081855	Control = BDL+ABT2 = BDL = BDL+ABT
88	noHit;	0.052941	0.082419	Control = BDL = BDL+ABT = BDL+ABT2
89	Bacteria;Verrucomicrobiota;Verrucomicrobiae;Verrucomicrobiales;Akkermansiaceae;?	0.055264	0.085069	Control = BDL+ABT = BDL+ABT2 = BDL
90	Bacteria;Bacteroidota;Bacteroidia;Bacteroidales;Bacteroidaceae;?	0.05787	0.08805	Control = BDL = BDL+ABT2 = BDL+ABT
91	Bacteria;Firmicutes;Clostridia;Oscillospirales;Ruminococcaceae;?	0.058486	0.08805	BDL+ABT2 = Control>= BDL+ABT = BDL
92	Bacteria;Firmicutes;Bacilli;Lactobacillales;Lactobacillaceae;Ligilactobacillus	0.061591	0.091717	BDL = BDL+ABT2 = BDL+ABT > Control
93	Bacteria;Firmicutes;Clostridia;Oscillospirales;[Eubacterium] coprostanoligenes group;?	0.063794	0.093229	Control = BDL = BDL+ABT = BDL+ABT2
94	Bacteria;Firmicutes;Bacilli;Lactobacillales;Lactobacillaceae;?	0.063967	0.093229	BDL = Control = BDL+ABT = BDL+ABT2
95	Bacteria;Firmicutes;Clostridia;Oscillospirales;Oscillospiraceae;Colidextribacter	0.068462	0.09873	BDL+ABT2 = BDL = Control = BDL+ABT
96	Bacteria;Firmicutes;Clostridia;Lachnospirales;Lachnospiraceae;Lachnospiraceae UCG-001	0.078172	0.111558	BDL+ABT = BDL = BDL+ABT2 = Control
97	Bacteria;Bacteroidota;Bacteroidia;Bacteroidales;Tannerellaceae;?	0.084302	0.119065	Control = BDL+ABT2 = BDL+ABT = BDL
98	Bacteria;Firmicutes;Bacilli;Lactobacillales;?;?	0.103811	0.145124	BDL+ABT = BDL+ABT2 = Control = BDL
99	Bacteria;Campylobacterota;Campylobacteria;Campylobacterales;Helicobacteraceae;Helicobacter	0.114862	0.158951	Control = BDL = BDL+ABT = BDL+ABT2
100	Bacteria;Verrucomicrobiota;Verrucomicrobiae;Verrucomicrobiales;?;?	0.124835	0.168421	BDL = Control = BDL+ABT2 = BDL+ABT
101	Bacteria;Firmicutes;Clostridia;Oscillospirales;?;metagenome	0.125321	0.168421	BDL+ABT = BDL = Control = BDL+ABT2
102	Bacteria;Firmicutes;Clostridia;Oscillospirales;Butyricicoccaceae;Butyricicoccus	0.125394	0.168421	BDL>= Control = BDL+ABT = BDL+ABT2
103	Bacteria;Firmicutes;Clostridia;?;?;?	0.139614	0.1857	BDL+ABT2 = Control = BDL+ABT = BDL
104	Bacteria;Firmicutes;Clostridia;Lachnospirales;Lachnospiraceae;Lachnospiraceae UCG-008	0.143081	0.188482	Control = BDL = BDL+ABT2>= BDL+ABT
105	Bacteria;Bacteroidota;Bacteroidia;Bacteroidales;Muribaculaceae;uncultured Bacteroidales bacterium	0.148079	0.193208	Control = BDL = BDL+ABT2 = BDL+ABT
106	Bacteria;Firmicutes;Clostridia;Lachnospirales;Lachnospiraceae;Lachnospiraceae UCG-006	0.154395	0.199548	BDL+ABT = BDL = BDL+ABT2 = Control
107	Bacteria;Actinobacteriota;Coriobacteriia;Coriobacteriales;Eggerthellaceae;?	0.159295	0.203957	BDL+ABT2 = BDL+ABT = BDL = Control
108	Bacteria;Actinobacteriota;Actinobacteria;Micrococcales;Micrococcaceae;?	0.176793	0.219915	Control = BDL = BDL+ABT = BDL+ABT2

109	Bacteria;Actinobacteriota;Actinobacteria;Propionibacteriales;Propionibacteriaceae;Cutibacterium	0.176793	0.219915	Control = BDL = BDL+ABT = BDL+ABT2
110	Bacteria;Proteobacteria;Gammaproteobacteria;Burkholderiales;Burkholderiaceae;Burkholderia-Caballeronia-Paraburkholderia	0.176793	0.219915	Control = BDL = BDL+ABT = BDL+ABT2
111	Bacteria;Firmicutes;Clostridia;Oscillospirales;UCG-010;?	0.178179	0.219915	Control = BDL = BDL+ABT = BDL+ABT2
112	Bacteria;Proteobacteria;Alphaproteobacteria;Rickettsiales;Mitochondria;?	0.199219	0.242162	BDL+ABT = Control = BDL = BDL+ABT2
113	Bacteria;Actinobacteriota;Actinobacteria;Corynebacteriales;Corynebacteriaceae;?	0.200727	0.242162	NA
114	Bacteria;Firmicutes;Bacilli;RF39;?;?	0.201507	0.242162	NA
115	Bacteria;Actinobacteriota;Coriobacteriia;Coriobacteriales;Atopobiaceae;?	0.204919	0.244121	NA
116	Bacteria;Firmicutes;Bacilli;Lactobacillales;Aerococcaceae;Facklamia	0.209798	0.247779	NA
117	Bacteria;Proteobacteria;Gammaproteobacteria;Enterobacterales;?;?	0.237299	0.277863	NA
118	Bacteria;Bacteroidota;Bacteroidia;Bacteroidales;Muribaculaceae;uncultured Barnesiella sp.	0.245308	0.284807	NA
119	Bacteria;Cyanobacteria;Cyanobacteriia;Chloroplast;?;?	0.261923	0.301542	NA
120	Bacteria;Bacteroidota;Bacteroidia;Bacteroidales;Prevotellaceae;Prevotellaceae UCG-001	0.301911	0.344681	NA
121	Bacteria;Firmicutes;Bacilli;RF39;uncultured Firmicutes bacterium;?	0.320762	0.357272	NA
122	Bacteria;Firmicutes;Clostridia;Lachnospirales;Lachnospiraceae;GCA-900066575	0.320762	0.357272	NA
123	Bacteria;Firmicutes;Clostridia;Oscillospirales;Oscillospiraceae;UCG-003	0.320762	0.357272	NA
124	Bacteria;Firmicutes;Bacilli;Lactobacillales;Carnobacteriaceae;Atopostipes	0.338174	0.373627	NA
125	Bacteria;Firmicutes;Clostridia;Clostridiales;?;?	0.368691	0.404086	NA
126	Bacteria;Firmicutes;Clostridia;Lachnospirales;Lachnospiraceae;Marvinbryantia	0.372407	0.404918	NA
127	Bacteria;Firmicutes;Bacilli;Erysipelotrichales;Erysipelatoclostridiaceae;Erysipelatoclostridium	0.375905	0.405503	NA
128	Bacteria;Bacteroidota;Bacteroidia;Bacteroidales;Rikenellaceae;Alistipes	0.388431	0.415743	NA
129	Bacteria;Firmicutes;Clostridia;Peptococcales;Peptococcaceae;Peptococcus	0.394996	0.419492	NA
130	Bacteria;Firmicutes;Bacilli;Staphylococcales;Staphylococcaceae;Jeotgalicoccus	0.414179	0.436481	NA
131	Bacteria;Firmicutes;Bacilli;Staphylococcales;Staphylococcaceae;?	0.493795	0.509461	NA
132	Bacteria;Desulfobacterota;Desulfovibrionia;Desulfovibrionales;Desulfovibrionaceae;Bilophila	0.498305	0.509461	NA
133	Bacteria;Proteobacteria;Gammaproteobacteria;Burkholderiales;Comamonadaceae;?	0.498305	0.509461	NA
134	Bacteria;Proteobacteria;Gammaproteobacteria;Burkholderiales;Comamonadaceae;Pelomonas	0.498305	0.509461	NA
135	Bacteria;Proteobacteria;Gammaproteobacteria;Burkholderiales;Burkholderiaceae;Ralstonia	0.559864	0.568158	NA
136	Bacteria;Firmicutes;Clostridia;Peptostreptococcales-Tissierellales;?;?	0.61084	0.615332	NA
137	Bacteria;Firmicutes;Clostridia;Lachnospirales;Lachnospiraceae;[Eubacterium] ventriosum group	0.988288	0.988288	NA

Species

Rank	Feature name	p-value	q-value	Direction
1	Bacteria;Firmicutes;Clostridia;Lachnospirales;Lachnospiraceae;Lachnoclostridium;bacterium NLAE-zl-H31	1.56E-05	0.001533	Control>> BDL = BDL+ABT = BDL+ABT2
2	Bacteria;Firmicutes;Clostridia;Clostridia UCG-014;?;?;?	3.82E-05	0.001533	BDL>> BDL+ABT2 > BDL+ABT >> Control
3	Bacteria;Firmicutes;Bacilli;Erysipelotrichales;Erysipelotrichaceae;?;?	4.21E-05	0.001533	Control>> BDL>> BDL+ABT2>= BDL+ABT
4	Bacteria;Actinobacteriota;Coriobacteriia;Coriobacteriales;Eggerthellaceae;Parvibacter;Adlercreutzia caecicola	4.75E-05	0.001533	BDL+ABT2 = BDL+ABT >> BDL>> Control
5	Bacteria;Bacteroidota;Bacteroidia;Bacteroidales;Muribaculaceae;uncultured Muribaculaceae bacterium;?	5.03E-05	0.001533	BDL > BDL+ABT >= BDL+ABT2>> Control
6	Bacteria;Bacteroidota;Bacteroidia;Bacteroidales;Rikenellaceae;Alistipes;uncultured Bacteroidales bacterium	5.37E-05	0.001533	Control>> BDL>= BDL+ABT = BDL+ABT2
7	Bacteria;Firmicutes;Clostridia;Clostridia vadinBB60 group;uncultured organism;?;?	7.40E-05	0.001533	Control>> BDL = BDL+ABT = BDL+ABT2
8	Bacteria;Firmicutes;Clostridia;Oscillospirales;Oscillospiraceae;?;?	9.01E-05	0.001533	BDL>= BDL+ABT2 > BDL+ABT >> Control
9	Bacteria;Proteobacteria;Gammaproteobacteria;Burkholderiales;?;?;?	9.91E-05	0.001533	Control>> BDL = BDL+ABT = BDL+ABT2
10	Bacteria;Firmicutes;Clostridia;Oscillospirales;Oscillospiraceae;Oscillibacter;Clostridiales bacterium	0.000101	0.001533	BDL+ABT2 = BDL+ABT = BDL>> Control
11	Bacteria;Bacteroidota;Bacteroidia;?;?;?;?	0.000106	0.001533	Control>> BDL = BDL+ABT = BDL+ABT2

12	Bacteria;Firmicutes;Clostridia;Clostridia vadinBB60 group;uncultured Clostridiales bacterium;?;?	0.000106	0.001533	Control>> BDL = BDL+ABT = BDL+ABT2
13	Bacteria;Bacteroidota;Bacteroidia;Bacteroidales;?;?;?	0.000117	0.001564	BDL+ABT2 = BDL+ABT >> BDL>> Control
14	Bacteria;Firmicutes;Bacilli;Erysipelotrichales;Erysipelotrichaceae;Faecalibaculum;?	0.000129	0.001608	Control>> BDL = BDL+ABT = BDL+ABT2
15	Bacteria;Bacteroidota;Bacteroidia;Bacteroidales;Tannerellaceae;Parabacteroides;Parabacteroides distasonis	0.000146	0.00169	BDL+ABT = BDL+ABT2>> Control > BDL
16	Bacteria;Firmicutes;Clostridia;Lachnospirales;Lachnospiraceae;Blautia;?	0.000159	0.00173	BDL = BDL+ABT2>= BDL+ABT >> Control
17	Bacteria;Firmicutes;Clostridia;Peptococcales;Peptococcaceae;?;?	0.000171	0.001746	BDL+ABT2>= BDL+ABT = BDL>> Control
18	Bacteria;Patescibacteria;Saccharimonadia;Saccharimonadales;Saccharimonadaceae;Candidatus Saccharimonas;?	0.000191	0.001771	BDL>> BDL+ABT = BDL+ABT2>> Control
19	Bacteria;Cyanobacteria;Vampirivibrionia;Gastranaerophilales;?;?;?	0.000193	0.001771	BDL+ABT2 = BDL+ABT >> BDL = Control
20	Bacteria;Firmicutes;Clostridia;Clostridia vadinBB60 group;?;?;?	0.000208	0.001808	Control>> BDL = BDL+ABT = BDL+ABT2
21	Bacteria;Firmicutes;Bacilli;Acholeplasmatales;Acholeplasmataceae;Anaeroplasma;?	0.000223	0.001848	BDL>> Control = BDL+ABT = BDL+ABT2
22	Bacteria;Firmicutes;Clostridia;Lachnospirales;Lachnospiraceae;Roseburia;?	0.000248	0.001877	Control>> BDL+ABT2 = BDL>= BDL+ABT
23	Bacteria;Firmicutes;Clostridia;Oscillospirales;Oscillospiraceae;NK4A214 group;?	0.000259	0.001877	BDL>> Control =

				BDL+ABT = BDL+ABT2
24	Bacteria;Deferribacterota;Deferribacteres;Deferribacterales;Deferribacteraceae;?;?	0.000259	0.001877	Control > BDL = BDL+ABT = BDL+ABT2
25	Bacteria;Firmicutes;Bacilli;Lactobacillales;Lactobacillaceae;Lactobacillus;Lactobacillus taiwanensis	0.000274	0.001906	BDL = Control> > BDL+ABT = BDL+ABT2
26	Bacteria;Bacteroidota;Bacteroidia;Bacteroidales;Bacteroidaceae;Bacteroides;Bacteroides acidifaciens	0.000305	0.002017	BDL+ABT2 = BDL+ABT >> BDL = Control
27	Bacteria;Bacteroidota;Bacteroidia;Bacteroidales;Bacteroidaceae;Bacteroides;?	0.000313	0.002017	BDL+ABT = BDL+ABT2>= BDL>= Control
28	Bacteria;Bacteroidota;Bacteroidia;Bacteroidales;Rikenellaceae;Alistipes;Alistipes obesi	0.000371	0.002069	BDL = BDL+ABT2 = BDL+ABT >> Control
29	Bacteria;Firmicutes;Clostridia;Lachnospirales;Lachnospiraceae;Blautia;Lachnospiraceae bacterium	0.000372	0.002069	BDL+ABT2 = BDL+ABT = BDL>> Control
30	Bacteria;Bacteroidota;Bacteroidia;Bacteroidales;Marinifilaceae;?;?	0.000374	0.002069	BDL+ABT = BDL+ABT2>= BDL > Control
31	Bacteria;Verrucomicrobiota;Verrucomicrobiae;Verrucomicrobiales;Akkermansiaceae;Akkermansia;Akkermansia muciniphila	0.000385	0.002069	Control > BDL = BDL+ABT > BDL+ABT2
32	Bacteria;Firmicutes;Clostridia;Lachnospirales;Lachnospiraceae;Lachnospiraceae FCS020 group;?	0.000402	0.002069	Control> > BDL+ABT2 = BDL > BDL+ABT
33	Bacteria;Firmicutes;Clostridia;Oscillospirales;[Eubacterium] coprostanoligenes group;gut metagenome;?	0.000404	0.002069	BDL>> Control = BDL+ABT = BDL+ABT2
34	Bacteria;Bacteroidota;Bacteroidia;Bacteroidales;Rikenellaceae;Rikenella;?	0.000426	0.002069	BDL+ABT = BDL+ABT2 =

35	Bacteria;Bacteroidota;Bacteroidia;Bacteroidales;Rikenellaceae;Alistipes;Alistipes timonensis	0.000427	0.002069	BDL>> Control BDL+ABT = BDL>= BDL+ABT 2 > Control
36	Bacteria;Bacteroidota;Bacteroidia;Bacteroidales;Muribaculaceae;mouse gut metagenome;?	0.000428	0.002069	Control> = BDL = BDL+ABT > BDL+ABT 2
37	Bacteria;Bacteroidota;Bacteroidia;Bacteroidales;Prevotellaceae;?;?	0.000457	0.002148	BDL = BDL+ABT = BDL+ABT 2>> Control
38	Bacteria;Firmicutes;Bacilli;Lactobacillales;Lactobacillaceae;Limosilactobacillus;?	0.000471	0.002158	BDL>= Control > BDL+ABT >= BDL+ABT 2
39	Bacteria;Verrucomicrobiota;Verrucomicrobiae;Verrucomicrobiales;Akkermansiaceae;Akkermansia;?	0.000513	0.002278	Control> > BDL = BDL+ABT 2 = BDL+ABT
40	Bacteria;Firmicutes;Bacilli;Lactobacillales;Lactobacillaceae;Lactobacillus;?	0.000524	0.002278	Control > BDL = BDL+ABT = BDL+ABT 2
41	Bacteria;Firmicutes;Clostridia;Lachnospirales;Lachnospiraceae;?;?	0.000566	0.0024	Control> > BDL+ABT = BDL = BDL+ABT 2
42	Bacteria;Firmicutes;Bacilli;Lactobacillales;Lactobacillaceae;Lactobacillus;Lactobacillus gasseri	0.000617	0.002556	Control > BDL = BDL+ABT = BDL+ABT 2
43	Bacteria;Firmicutes;Clostridia;Oscillospirales;Butyricicoccaceae;?;?	0.000654	0.002647	BDL = BDL+ABT 2 = BDL+ABT >> Control
44	Bacteria;Bacteroidota;Bacteroidia;Bacteroidales;Rikenellaceae;?;?	0.000806	0.003141	BDL>= BDL+ABT >= BDL+ABT 2 = Control
45	Bacteria;Proteobacteria;Gammaproteobacteria;Burkholderiales;Sutterellaceae;?;?	0.000816	0.003141	Control = BDL+ABT >= BDL = BDL+ABT 2

46	Bacteria;Firmicutes;Clostridia;Peptostreptococcales-Tissierellales;Anaerovoracaceae;[Eubacterium] brachy group;?	0.000 83	0.003141	Control > BDL+ABT 2 = BDL+ABT = BDL
47	Bacteria;Actinobacteriota;Coriobacteriia;Coriobacteriales;Eggerthellaceae;Enterorhabdus;mouse gut	0.000 897	0.00332	BDL+ABT = BDL+ABT 2 = BDL>> Control
48	Bacteria;Firmicutes;Clostridia;Clostridiales;Clostridiaceae;?;?	0.001 044	0.003786	BDL = BDL+ABT 2 = BDL+ABT >> Control
49	Bacteria;Bacteroidota;Bacteroidia;Bacteroidales;Bacteroidaceae;Bacteroides;Bacteroides caecimuris	0.001 107	0.003931	Control = BDL+ABT = BDL+ABT 2>= BDL
50	Bacteria;Bacteroidota;Bacteroidia;Bacteroidales;Muribaculaceae;uncultured organism;?	0.001 13	0.003934	Control = BDL = BDL+ABT > BDL+ABT 2
51	Bacteria;Bacteroidota;Bacteroidia;Bacteroidales;Rikenellaceae;Rikenellaceae RC9 gut group;uncultured organism	0.001 162	0.003963	BDL > Control = BDL+ABT = BDL+ABT 2
52	Bacteria;Bacteroidota;Bacteroidia;Bacteroidales;Marinifilaceae;Odoribacter;?	0.001 247	0.004174	BDL+ABT = BDL = BDL+ABT 2>> Control
53	Bacteria;Bacteroidota;Bacteroidia;Bacteroidales;Rikenellaceae;Rikenellaceae RC9 gut group;?	0.001 307	0.00429	BDL > Control = BDL+ABT = BDL+ABT 2
54	Bacteria;Actinobacteriota;Coriobacteriia;Coriobacteriales;Atopobiaceae;Olsenella;?	0.001 363	0.004393	Control = BDL+ABT = BDL+ABT 2 = BDL
55	Bacteria;Firmicutes;Clostridia;Christensenellales;Christensenellaceae;?;?	0.001 564	0.004869	BDL = BDL+ABT = BDL+ABT 2>> Control
56	Bacteria;Firmicutes;Clostridia;Peptostreptococcales-Tissierellales;Anaerovoracaceae;?;?	0.001 567	0.004869	BDL+ABT 2 = BDL = BDL+ABT >> Control
57	Bacteria;Patescibacteria;Saccharimonadia;Saccharimonadales;Saccharimonadaceae;?;?	0.001 697	0.005122	Control = BDL+ABT 2 =

58	Bacteria;Proteobacteria;Gammaproteobacteria;Enterobacterales;Enterobacteriaceae;Escherichia-Shigella;?	0.001707	0.005122	BDL>= BDL+ABT Control> = BDL = BDL+ABT = BDL+ABT 2
59	Bacteria;Proteobacteria;Gammaproteobacteria;Enterobacterales;Enterobacteriaceae;Escherichia-Shigella;Escherichia coli	0.001872	0.005521	BDL+ABT 2 = BDL+ABT = BDL>> Control
60	Bacteria;Firmicutes;Clostridia;Oscillospirales;Ruminococcaceae;Incertae Sedis;?	0.002044	0.005929	Control> = BDL = BDL+ABT = BDL+ABT 2
61	Bacteria;Firmicutes;Clostridia;Oscillospirales;Butyricicoccaceae;UCG-009;[Clostridium] leptum	0.002233	0.006319	BDL+ABT 2 = BDL = BDL+ABT >> Control
62	Bacteria;Bacteroidota;Bacteroidia;Bacteroidales;Muribaculaceae;?;?	0.002251	0.006319	BDL = BDL+ABT = BDL+ABT 2 > Control
63	Bacteria;Firmicutes;Bacilli;Lactobacillales;Enterococcaceae;Enterococcus;Enterococcus faecalis	0.002493	0.0068	BDL+ABT = BDL+ABT 2>= Control = BDL
64	Bacteria;?;?;?;?;?;?	0.002501	0.0068	Control > BDL = BDL+ABT 2 = BDL+ABT
65	Bacteria;Proteobacteria;Gammaproteobacteria;Enterobacterales;Enterobacteriaceae;?;?	0.002872	0.007689	BDL = BDL+ABT = BDL+ABT 2>> Control
66	Bacteria;Desulfobacterota;Desulfovibrionia;Desulfovibrionales;Desulfovibrionaceae;?;?	0.003055	0.007956	Control = BDL+ABT 2 = BDL = BDL+ABT
67	Bacteria;Firmicutes;Bacilli;Erysipelotrichales;Erysipelatoclostridiaceae;?;?	0.003064	0.007956	BDL+ABT = BDL+ABT 2 = BDL>> Control
68	Bacteria;Firmicutes;Bacilli;Erysipelotrichales;Erysipelatoclostridiaceae;Candidatus Stoquefichus;?	0.00312	0.007984	Control > BDL = BDL+ABT = BDL+ABT 2

69	Bacteria;Firmicutes;Clostridia;Christensenellales;Christensenellaceae;Christensenella sp. Marseille-P2437;?	0.003226	0.008135	BDL = BDL+ABT2 = BDL+ABT > Control
70	Bacteria;Firmicutes;Bacilli;Erysipelotrichales;Erysipelotrichaceae;Faecalibaculum;Faecalibaculum rodentium	0.003469	0.008624	BDL+ABT2 > BDL+ABT = BDL = Control
71	Bacteria;Proteobacteria;Gammaproteobacteria;Burkholderiales;Sutterellaceae;Parasutterella;?	0.003671	0.008997	Control = BDL+ABT2 = BDL > BDL+ABT
72	Bacteria;Firmicutes;Clostridia;Lachnospirales;?;?;?	0.003861	0.009331	Control > BDL+ABT = BDL+ABT2 = BDL
73	Bacteria;Cyanobacteria;Vampirivibrionia;Gastranaerophilales;uncultured rumen bacterium;?;?	0.003943	0.009398	BDL+ABT = BDL+ABT2 = BDL>= Control
74	Bacteria;Firmicutes;Bacilli;?;?;?;?	0.004441	0.010442	Control = BDL = BDL+ABT = BDL+ABT2
75	Bacteria;Firmicutes;Bacilli;Lactobacillales;Enterococcaceae;Enterococcus;?	0.004658	0.010808	BDL > Control = BDL+ABT = BDL+ABT2
76	Bacteria;Proteobacteria;Alphaproteobacteria;Rhodospirillales;?;?;?	0.004853	0.011112	BDL+ABT > Control> = BDL+ABT2 = BDL
77	Bacteria;Actinobacteriota;Coriobacteriia;Coriobacteriales;Eggerthellaceae;DNF00809;?	0.005539	0.012518	BDL+ABT = BDL+ABT2 = BDL>= Control
78	Bacteria;Actinobacteriota;Actinobacteria;Corynebacteriales;Corynebacteriaceae;Corynebacterium;?	0.006234	0.01381	BDL = BDL+ABT2 = BDL+ABT > Control
79	Bacteria;Bacteroidota;Bacteroidia;Bacteroidales;Tannerellaceae;Parabacteroides;Parabacteroides goldsteinii	0.00627	0.01381	BDL+ABT = BDL+ABT2 = Control> > BDL
80	Bacteria;Firmicutes;Clostridia;Lachnospirales;Lachnospiraceae;A2;?	0.00647	0.014071	BDL = BDL+ABT2 = BDL+ABT = Control

81	Bacteria;Deferribacterota;Deferribacteres;Deferribacterales;Deferribacteraceae;Mucispirillum;Mucispirillum schaedleri	0.006648	0.01428	Control> = BDL = BDL+ABT = BDL+ABT 2
82	Bacteria;Actinobacteriota;Actinobacteria;Corynebacteriales;Corynebacteriaceae;Corynebacterium;Corynebacterium lowii	0.007346	0.015587	BDL > Control = BDL+ABT = BDL+ABT 2
83	Bacteria;Bacteroidota;Bacteroidia;Bacteroidales;Tannerellaceae;Parabacteroides;?	0.007753	0.016079	BDL+ABT 2 = BDL+ABT = Control> = BDL
84	Bacteria;Firmicutes;Clostridia;Lachnospirales;Lachnospiraceae;Lachnospiraceae NK4A136 group;?	0.007762	0.016079	BDL+ABT 2 = BDL+ABT = BDL > Control
85	Bacteria;Proteobacteria;Gammaproteobacteria;Enterobacterales;Pasteurellaceae;?;?	0.007875	0.01612	BDL+ABT >= Control = BDL = BDL+ABT 2
86	Bacteria;Proteobacteria;Gammaproteobacteria;Pseudomonadales;?;?;?	0.009973	0.020153	BDL = BDL+ABT 2>= Control = BDL+ABT
87	Bacteria;Firmicutes;Bacilli;Acholeplasmatales;Acholeplasmataceae;?;?	0.010076	0.020153	BDL>= Control = BDL+ABT = BDL+ABT 2
88	Bacteria;Firmicutes;Clostridia;Lachnospirales;Lachnospiraceae;[Eubacterium] xylanophilum group;?	0.010291	0.020349	BDL+ABT 2 = BDL = Control = BDL+ABT
89	Bacteria;Firmicutes;Clostridia;Oscillospirales;?;?;?	0.011575	0.022379	BDL+ABT 2 = BDL+ABT = BDL = Control
90	Bacteria;Firmicutes;Clostridia;Christensenellales;?;?;?	0.011575	0.022379	BDL+ABT 2 > Control = BDL = BDL+ABT
91	Bacteria;Firmicutes;Bacilli;Lactobacillales;Streptococcaceae;Streptococcus;?	0.012004	0.022691	BDL+ABT = BDL+ABT 2 = BDL>= Control
92	Bacteria;Actinobacteriota;Coriobacteriia;Coriobacteriales;Eggerthellaceae;?;uncultured Coriobacteriales bacterium	0.012091	0.022691	BDL+ABT = BDL+ABT 2 =

93	Bacteria;Actinobacteriota;Coriobacteriia;Coriobacteriales;Eggerthellaceae;Enterorhabdus;?	0.012131	0.022691	Control = BDL BDL+ABT = BDL+ABT 2 = BDL = Control
94	Bacteria;Actinobacteriota;?;?;?;?;?	0.012258	0.022691	Control = BDL+ABT 2 = BDL+ABT = BDL
95	Bacteria;Bacteroidota;Bacteroidia;Bacteroidales;Muribaculaceae;Muribaculum;Muribaculum intestinale	0.013756	0.024933	Control> = BDL = BDL+ABT = BDL+ABT 2
96	Bacteria;Firmicutes;Clostridia;Oscillospirales;Ruminococcaceae;Incertae Sedis;Acutalibacter muris	0.013756	0.024933	Control> = BDL = BDL+ABT = BDL+ABT 2
97	Bacteria;Firmicutes;Bacilli;Lactobacillales;Lactobacillaceae;HT002;Lactobacillus sp.	0.014068	0.025235	BDL = Control = BDL+ABT = BDL+ABT 2
98	Bacteria;Desulfobacterota;Desulfovibrionia;Desulfovibrionales;Desulfovibrionaceae;Desulfovibrio;?	0.014242	0.025287	BDL+ABT 2 = Control = BDL = BDL+ABT
99	Bacteria;Firmicutes;Clostridia;Lachnospirales;Lachnospiraceae;Lachnoclostridium;?	0.015408	0.02708	Control = BDL+ABT 2 = BDL = BDL+ABT
100	Bacteria;Firmicutes;Bacilli;Staphylococcales;Staphylococcaceae;Staphylococcus;Staphylococcus lentus	0.016645	0.028963	BDL > BDL+ABT = BDL+ABT 2 = Control
101	Bacteria;Bacteroidota;Bacteroidia;Bacteroidales;Muribaculaceae;Muribaculum;?	0.020499	0.035314	Control = BDL+ABT = BDL = BDL+ABT 2
102	Bacteria;Firmicutes;Clostridia;Oscillospirales;Ruminococcaceae;Harryflintia;?	0.022753	0.038815	BDL+ABT 2 = BDL+ABT >= Control = BDL
103	Bacteria;Firmicutes;?;?;?;?;?	0.024649	0.04164	Control = BDL+ABT 2 = BDL = BDL+ABT
104	Bacteria;Firmicutes;Clostridia;Lachnospirales;Lachnospiraceae;Anaerostipes;Anaerostipes caccae	0.026294	0.043991	Control = BDL = BDL+ABT =

				BDL+ABT 2
105	Bacteria;Firmicutes;Clostridia;Oscillospirales;Oscillospiraceae;UCG-005;?	0.027634	0.045794	BDL+ABT 2 > Control = BDL = BDL+ABT
106	Bacteria;Cyanobacteria;Vampirivibrionia;Gastranaerophilales;gut metagenome;?;?	0.036891	0.060407	BDL+ABT 2 = Control = BDL = BDL+ABT
107	Bacteria;Actinobacteriota;Coriobacteriia;Coriobacteriales;?;?;?	0.037147	0.060407	Control = BDL = BDL+ABT = BDL+ABT 2
108	Bacteria;Firmicutes;Clostridia;Oscillospirales;Ruminococcaceae;Anaerotruncus;?	0.051981	0.083742	Control = BDL+ABT 2 = BDL = BDL+ABT
109	Bacteria;Firmicutes;Bacilli;Lactobacillales;Carnobacteriaceae;Atopostipes;?	0.052941	0.083742	Control = BDL = BDL+ABT = BDL+ABT 2
110	noHit;	0.052941	0.083742	Control = BDL = BDL+ABT = BDL+ABT 2
111	Bacteria;Verrucomicrobiota;Verrucomicrobiae;Verrucomicrobiales;Akkermansiaceae;?;?	0.055264	0.08663	Control = BDL+ABT = BDL+ABT 2 = BDL
112	Bacteria;Bacteroidota;Bacteroidia;Bacteroidales;Bacteroidaceae;?;?	0.05787	0.089905	Control = BDL = BDL+ABT 2 = BDL+ABT
113	Bacteria;Firmicutes;Clostridia;Oscillospirales;Ruminococcaceae;?;?	0.058486	0.090058	BDL+ABT 2 = Control> = BDL+ABT = BDL
114	Bacteria;Firmicutes;Bacilli;Lactobacillales;Lactobacillaceae;Ligilactobacillus;?	0.061591	0.094007	BDL = BDL+ABT 2 = BDL+ABT > Control
115	Bacteria;Firmicutes;Clostridia;Oscillospirales;[Eubacterium] coprostanoligenes group;?;?	0.063794	0.095951	Control = BDL = BDL+ABT = BDL+ABT 2
116	Bacteria;Firmicutes;Bacilli;Lactobacillales;Lactobacillaceae;?;?	0.063967	0.095951	BDL = Control = BDL+ABT =

				BDL+ABT 2
117	Bacteria;Firmicutes;Clostridia;Oscillospirales;Oscillospiraceae;Colidextribacter;?	0.068462	0.101816	BDL+ABT 2 = BDL = Control = BDL+ABT
118	Bacteria;Firmicutes;Clostridia;Lachnospirales;Lachnospiraceae;Lachnospiraceae NK4A136 group;Clostridiales bacterium	0.076159	0.112302	BDL+ABT 2 = BDL = BDL+ABT = Control
119	Bacteria;Firmicutes;Clostridia;Lachnospirales;Lachnospiraceae;Lachnospiraceae UCG-001;?	0.078172	0.114302	BDL+ABT = BDL = BDL+ABT 2 = Control
120	Bacteria;Bacteroidota;Bacteroidia;Bacteroidales;Tannerellaceae;?;?	0.084302	0.122237	Control = BDL+ABT 2 = BDL+ABT = BDL
121	Bacteria;Firmicutes;Bacilli;Lactobacillales;?;?;?	0.103811	0.149283	BDL+ABT = BDL+ABT 2 = Control = BDL
122	Bacteria;Campylobacterota;Campylobacteria;Campylobacterales;Helicobacteraceae;Helicobacter;Helicobacter typhlonius	0.114862	0.16382	Control = BDL = BDL+ABT = BDL+ABT 2
123	Bacteria;Firmicutes;Bacilli;Staphylococcales;Staphylococcaceae;Staphylococcus;?	0.116866	0.165322	BDL = BDL+ABT 2 = Control = BDL+ABT
124	Bacteria;Verrucomicrobiota;Verrucomicrobiae;Verrucomicrobiales;?;?;?	0.124835	0.173163	BDL = Control = BDL+ABT 2 = BDL+ABT
125	Bacteria;Firmicutes;Clostridia;Oscillospirales;?;metagenome;?	0.125321	0.173163	BDL+ABT = BDL = Control = BDL+ABT 2
126	Bacteria;Firmicutes;Clostridia;Oscillospirales;Butyricicoccaceae;Butyricicoccus;?	0.125394	0.173163	BDL>= Control = BDL+ABT = BDL+ABT 2
127	Bacteria;Firmicutes;Clostridia;?;?;?;?	0.139614	0.191282	BDL+ABT 2 = Control = BDL+ABT = BDL
128	Bacteria;Firmicutes;Clostridia;Lachnospirales;Lachnospiraceae;Lachnospiraceae UCG-008;?	0.143081	0.194501	Control = BDL = BDL+ABT 2>= BDL+ABT

129	Bacteria;Bacteroidota;Bacteroidia;Bacteroidales;Muribaculaceae;uncultured Bacteroidales bacterium;?	0.148079	0.199735	Control = BDL = BDL+ABT2 = BDL+ABT
130	Bacteria;Firmicutes;Clostridia;Lachnospirales;Lachnospiraceae;Lachnospiraceae UCG-006;?	0.154395	0.206651	BDL+ABT = BDL = BDL+ABT2 = Control
131	Bacteria;Actinobacteriota;Coriobacteriia;Coriobacteriales;Eggerthellaceae;?;?	0.174333	0.227866	BDL+ABT2 = BDL+ABT = BDL = Control
132	Bacteria;Actinobacteriota;Actinobacteria;Micrococcales;Micrococcaceae;?;?	0.176793	0.227866	Control = BDL = BDL+ABT = BDL+ABT2
133	Bacteria;Actinobacteriota;Actinobacteria;Propionibacteriales;Propionibacteriaceae;Cutibacterium;Cutibacterium granulosum	0.176793	0.227866	Control = BDL = BDL+ABT = BDL+ABT2
134	Bacteria;Firmicutes;Clostridia;Lachnospirales;Lachnospiraceae;Lachnoclostridium;[Clostridium] bolteae	0.176793	0.227866	Control = BDL = BDL+ABT = BDL+ABT2
135	Bacteria;Proteobacteria;Gammaproteobacteria;Burkholderiales;Sutterellaceae;Parasutterella;Turicimonas muris	0.176793	0.227866	Control = BDL = BDL+ABT = BDL+ABT2
136	Bacteria;Firmicutes;Clostridia;Oscillospirales;UCG-010;?;?	0.178179	0.227965	Control = BDL = BDL+ABT = BDL+ABT2
137	Bacteria;Proteobacteria;Alphaproteobacteria;Rickettsiales;Mitochondria;?;?	0.199219	0.250445	BDL+ABT = Control = BDL = BDL+ABT2
138	Bacteria;Actinobacteriota;Actinobacteria;Corynebacteriales;Corynebacteriaceae;?;?	0.200727	0.250445	NA
139	Bacteria;Firmicutes;Clostridia;Lachnospirales;Lachnospiraceae;Marvinbryantia;Clostridiales bacterium	0.200727	0.250445	NA
140	Bacteria;Firmicutes;Bacilli;RF39;?;?;?	0.201507	0.250445	NA
141	Bacteria;Actinobacteriota;Coriobacteriia;Coriobacteriales;Atopobiaceae;?;?	0.204919	0.252879	NA
142	Bacteria;Firmicutes;Bacilli;Lactobacillales;Aerococcaceae;Facklamia;Facklamia tabacinasalis	0.209798	0.257077	NA
143	Bacteria;Proteobacteria;Gammaproteobacteria;Enterobacterales;?;?;?	0.237299	0.288357	NA
144	Bacteria;Firmicutes;Clostridia;Oscillospirales;Oscillospiraceae;Oscillibacter;?	0.23864	0.288357	NA
145	Bacteria;Bacteroidota;Bacteroidia;Bacteroidales;Muribaculaceae;uncultured Barnesiella sp.;?	0.245308	0.29437	NA

146	Bacteria;Cyanobacteria;Cyanobacteriia;Chloroplast;?;?;?	0.261923	0.312155	NA
147	Bacteria;Firmicutes;Clostridia;Lachnospirales;Lachnospiraceae;Marvinbryantia;?	0.269095	0.31852	NA
148	Bacteria;Actinobacteriota;Coriobacteriia;Coriobacteriales;Eggerthellaceae;Parvibacter;?	0.28675	0.337125	NA
149	Bacteria;Firmicutes;Bacilli;Lactobacillales;Carnobacteriaceae;Atopostipes;Firmicutes oral	0.301044	0.350216	NA
150	Bacteria;Bacteroidota;Bacteroidia;Bacteroidales;Prevotellaceae;Prevotellaceae UCG-001;?	0.301911	0.350216	NA
151	Bacteria;Actinobacteriota;Actinobacteria;Corynebacteriales;Corynebacteriaceae;Corynebacterium;Corynebacterium accolens	0.320762	0.355494	NA
152	Bacteria;Bacteroidota;Bacteroidia;Bacteroidales;Bacteroidaceae;Bacteroides;Bacteroides uniformis	0.320762	0.355494	NA
153	Bacteria;Bacteroidota;Bacteroidia;Bacteroidales;Rikenellaceae;Alistipes;Alistipes finegoldii	0.320762	0.355494	NA
154	Bacteria;Bacteroidota;Bacteroidia;Bacteroidales;Tannerellaceae;Parabacteroides;Parabacteroides merdae	0.320762	0.355494	NA
155	Bacteria;Firmicutes;Bacilli;RF39;uncultured Firmicutes bacterium;?;?	0.320762	0.355494	NA
156	Bacteria;Firmicutes;Clostridia;Lachnospirales;Lachnospiraceae;GCA-900066575;?	0.320762	0.355494	NA
157	Bacteria;Firmicutes;Clostridia;Oscillospirales;Oscillospiraceae;UCG-003;?	0.320762	0.355494	NA
158	Bacteria;Bacteroidota;Bacteroidia;Bacteroidales;Rikenellaceae;Alistipes;?	0.365631	0.402657	NA
159	Bacteria;Firmicutes;Clostridia;Clostridiales;?;?;?	0.368691	0.403473	NA
160	Bacteria;Firmicutes;Bacilli;Erysipelotrichales;Erysipelatoclostridiaceae;Erysipelatoclostridium;?	0.375905	0.408796	NA
161	Bacteria;Firmicutes;Clostridia;Peptococcales;Peptococcaceae;Peptococcus;?	0.394996	0.42689	NA
162	Bacteria;Actinobacteriota;Actinobacteria;Corynebacteriales;Corynebacteriaceae;Corynebacterium;Corynebacterium ammoniagenes	0.414179	0.44213	NA
163	Bacteria;Firmicutes;Bacilli;Staphylococcales;Staphylococcaceae;Jeotgalicoccus;?	0.414179	0.44213	NA
164	Bacteria;Firmicutes;Clostridia;Oscillospirales;Oscillospiraceae;Colidextribacter;Clostridiales bacterium	0.469436	0.49806	NA
165	Bacteria;Firmicutes;Bacilli;Staphylococcales;Staphylococcaceae;?;?	0.493795	0.507048	NA
166	Bacteria;Desulfobacterota;Desulfovibrionia;Desulfovibrionales;Desulfovibrionaceae;Bilophila;?	0.498305	0.507048	NA
167	Bacteria;Firmicutes;Clostridia;Lachnospirales;Lachnospiraceae;Anaerostipes;?	0.498305	0.507048	NA
168	Bacteria;Proteobacteria;Gammaproteobacteria;Burkholderiales;Burkholderiaceae;Burkholderia-Caballeronia-Paraburkholderia;Paraburkholderia ferrariae	0.498305	0.507048	NA
169	Bacteria;Proteobacteria;Gammaproteobacteria;Burkholderiales;Burkholderiaceae;Burkholderia-Caballeronia-Paraburkholderia;Paraburkholderia susongensis	0.498305	0.507048	NA
170	Bacteria;Proteobacteria;Gammaproteobacteria;Burkholderiales;Comamonadaceae;?;?	0.498305	0.507048	NA
171	Bacteria;Proteobacteria;Gammaproteobacteria;Burkholderiales;Comamonadaceae;Pelomonas;Pelomonas puraquae	0.498305	0.507048	NA
172	Bacteria;Proteobacteria;Gammaproteobacteria;Burkholderiales;Burkholderiaceae;Ralstonia;?	0.559864	0.566374	NA
173	Bacteria;Firmicutes;Clostridia;Peptostreptococcales-Tissierellales;?;?;?	0.61084	0.614371	NA
174	Bacteria;Firmicutes;Clostridia;Lachnospirales;Lachnospiraceae;[Eubacterium] ventriosum group;?	0.988288	0.988288	NA

Table S3. Bacterial taxa after 16s rRNA amplicon sequencing of mouse faecal samples. 16s rRNA sequencing of faecal samples from control, BDL/ABT and BDL/ABT2 mice. Analyses were done from n=5-7 mice. (control vs BDL vs BDL/ABT vs BDL/ABT2).

Supplementary references

[1] Blokker BA, Maijo M, Echeandia M, et al. Fine-Tuning of Sirtuin 1 Expression Is Essential to Protect the Liver From Cholestatic Liver Disease. Hepatology 2019;69:699-716.
[2] Demaria M, Ohtani N, Youssef SA, et al. An essential role for senescent cells in optimal wound healing through secretion of PDGF-AA. Dev Cell 2014;31:722-733.
[3] Schindelin J, Arganda-Carreras I, Frise E, et al. Fiji: an open-source platform for biological-image analysis. Nat Methods 2012;9:676-682.
[4] Isaacs-Ten A, Echeandia M, Moreno-Gonzalez M, et al. Intestinal Microbiome-Macrophage Crosstalk Contributes to Cholestatic Liver Disease by Promoting Intestinal Permeability in Mice. Hepatology 2020;72:2090-2108.
[5] Sato T, Vries RG, Snippert HJ, et al. Single Lgr5 stem cells build crypt-villus structures in vitro without a mesenchymal niche. Nature 2009;459:262-265.
[6] Allan C, Burel JM, Moore J, et al. OMERO: flexible, model-driven data management for experimental biology. Nat Methods 2012;9:245-253.
[7] Goldberg IG, Allan C, Burel JM, et al. The Open Microscopy Environment (OME) Data Model and XML file: open tools for informatics and quantitative analysis in biological imaging. Genome Biol 2005;6:R47.
[8] Ozkurt E, Fritscher J, Soranzo N, et al. LotuS2: an ultrafast and highly accurate tool for amplicon sequencing analysis. Microbiome 2022;10:176.
[9] Yilmaz P, Parfrey LW, Yarza P, et al. The SILVA and "All-species Living Tree Project (LTP)" taxonomic frameworks. Nucleic Acids Res 2014;42:D643-648.
[10] Bedarf JR, Beraza N, Khazneh H, et al. Much ado about nothing? Off-target amplification can lead to false-positive bacterial brain microbiome detection in healthy and Parkinson's disease individuals. Microbiome 2021;9:75.
[11] Hildebrand F, Moitinho-Silva L, Blasche S, et al. Antibiotics-induced monodominance of a novel gut bacterial order. Gut 2019.
[12] Saary P, Forslund K, Bork P, et al. RTK: efficient rarefaction analysis of large datasets. Bioinformatics 2017;33:2594-2595.
