# Supplementary material for: Regulation of intestinal senescence during cholestatic liver disease modulates barrier function and liver disease progression
Source: JHEP Rep. 2024 Jun 29;6(10):101159. doi: 10.1016/j.jhepr.2024.101159 (PMC11418120; doi:10.1016/j.jhepr.2024.101159)

Fig 3F (EtOH/DCA)

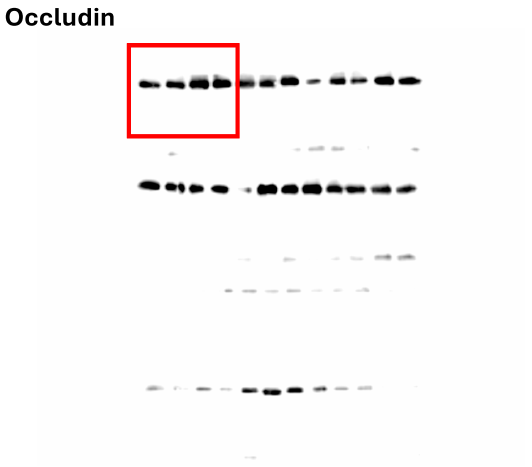

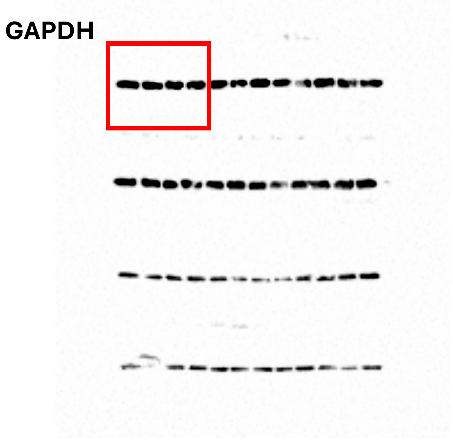

Fig 3F (Ctrl/LPS)

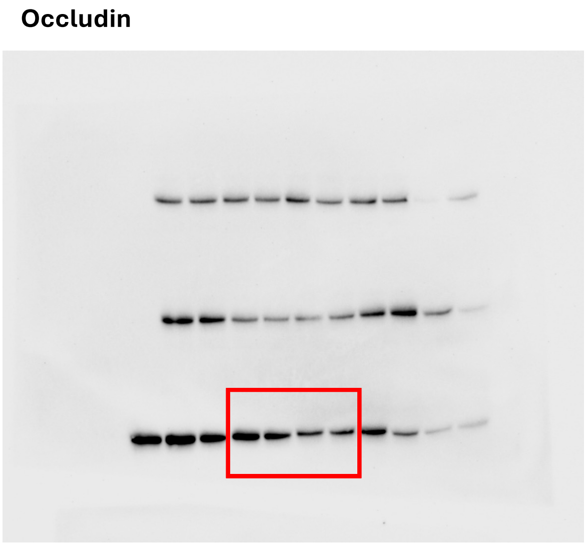

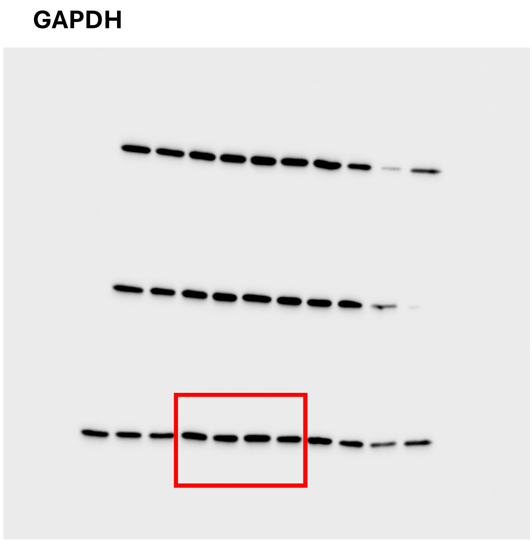

Fig 3I (EtOH/DCA)

p-p38

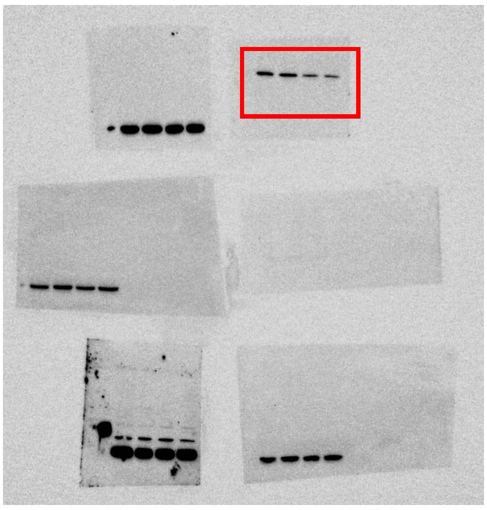

GAPDH

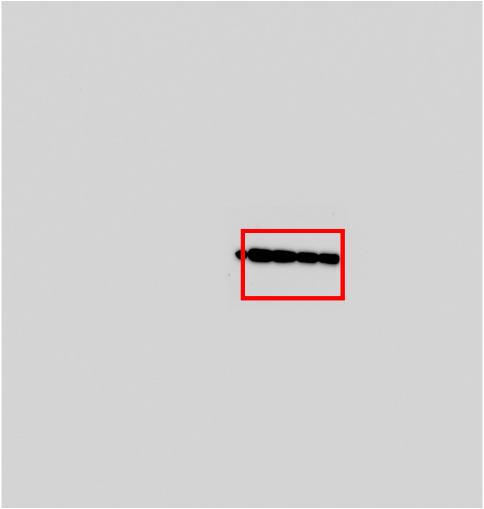

Fig 3I (Ctrl/LPS)

p-p38

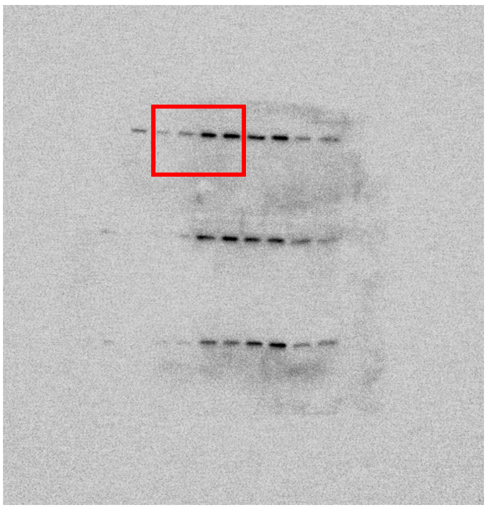

GAPDH

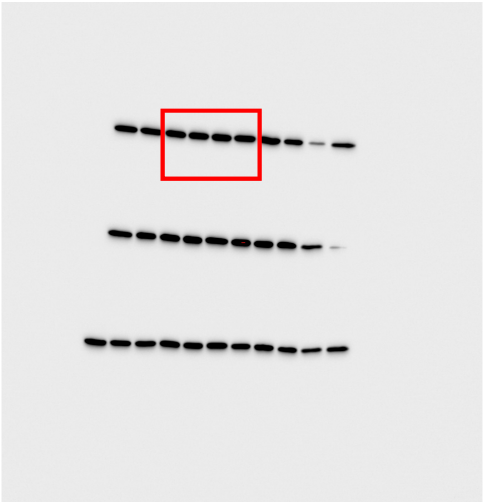

Supplement: Multimedia component 2 [file mmc2.pdf]
