# Supplementary material for: Regulation of intestinal senescence during cholestatic liver disease modulates barrier function and liver disease progression
Source: JHEP Rep. 2024 Jun 29;6(10):101159. doi: 10.1016/j.jhepr.2024.101159 (PMC11418120; doi:10.1016/j.jhepr.2024.101159)
Supplement: Multimedia component 3 [file mmc3.docx]

**Journal of Hepatology**

**CTAT methods**

Tables for a “Complete, Transparent, Accurate and Timely account” (CTAT) are now mandatory for all revised submissions. The aim is to enhance the reproducibility of methods.

- Only include the parts relevant to your study
- Refer to the CTAT in the main text as ‘Supplementary CTAT Table’
- Do not add subheadings
- Add as many rows as needed to include all information
- Only include one item per row

**If the CTAT form is not relevant to your study, please outline the reasons why:**

|  |
| --- |

- 1. **Antibodies**

| **Name** | **Citation** | **Supplier** | **Cat no.** | **Clone no.** |
| --- | --- | --- | --- | --- |
| Anti-Ki67 antibody | PMID: 34420159 | Abcam | AB15580 | N/A  Polyclonal |
| Anti-4 Hydroxynonenal antibody (4HNE) | PMID: 36633130 | Abcam | AB46545 | N/A Polyclonal |
| Anti-CDKN2A/p16INK4a antibody | PMID: 35159350 | Abcam | AB54210 | 2D9A12 |
| EnVision^+^polymer HRP labelled anti-mouse | N/A | Dako | K4001 | N/A |
| EnVision^+^polymer HRP labelled anti-rabbit | N/A | Dako | K4003 | N/A |
| LGR5 Monoclonal Antibody | PMID: 35008827 | Thermo Fisher | MA5-25644 | OTI2A2 |
| Phospho-p38 MAPK (Thr180/Tyr182) Antibody | PMID: 36475068 | Cell Signaling Technologies | 9211 | N/A Polyclonal |
| Recombinant Anti-Occludin antibody | PMID: 20152177 | Abcam | AB216327 | EPR20992 |
| Anti-GAPDH antibody | PMID: 36414374 | Abcam | ab8245 | 6C5 |
| Anti-rabbit IgG-HRP-linked | PMID: 32913449 | Cell Signaling Technologies | 7074S | N/A |
| Anti-mouse IgG-HRP-linked | PMID: 34107251 | Cell Signaling Technologies | 7076S | N/A |

- 1. **Cell lines**

| **Name** | **Citation** | **Supplier** | **Cat no.** | **Passage no.** | **Authentication test method** |
| --- | --- | --- | --- | --- | --- |
| Human CaCo-2 cells | PMID: 32117106 |  |  | **40** |  |

- 1. **Organisms**

| **Name** | **Citation** | **Supplier** | **Strain** | **Sex** | **Age** | **Overall n number** |
| --- | --- | --- | --- | --- | --- | --- |
| WT mice | PMID: 32168395 | Disease Modelling Unit (University of East Anglia, UK). | C57BL/6 | M | 8-12 weeks | 112 |
| P16 mice | **PDMI: 25499914** | UNITY Biotechnology, Inc. (USA). | P16-3MR om C57/BL6 | M | 8-12 weeks | 16 |

- 1. **Sequence based reagents**

| **Name** | **Sequence** | **Supplier** |
| --- | --- | --- |
| Mouse TBP1 primer | Forward: GAAGCTGCGGTACATTCCAG  Reverse: CCTTGTACCCTTCACCAAT | Sigma |
| E.coli primer | Forward: CGATAATCGCCAGATGGC  Reverse: CCTAAGTTGCAGGAGATGG | Sigma |
| Mouse LGR5 primer | Forward: TCACTCCAGTCCACTGGTTC  Reverse: GGCACCTGTGTCATCATCCA | Sigma |
| Mouse p16 primer | Forward: TCTCACCTCGCTTGTCACAG  Reverse: CGGCCCTCTTCTCAAGATCC | Sigma |
| 16S Uni primer | Forward: GTGSTGCAYGGYYGTCGTCA  Reverse: ACGTCRTCCMCNCCTTCCTC | Sigma |

- 1. **Biological samples**

| **Description** | **Source** | **Identifier** |
| --- | --- | --- |
| Colonic tissue from PSC patients | Norwich Research Park (NRP) BioRepository |  |
| Colonic tissue from healthy patients | MOTION study, Quadram Institute |  |

- 1. **Deposited data**

| **Name of repository** | **Identifier** | **Link** |
| --- | --- | --- |
| Norwich Research Park (NRP) BioRepository |  | https://biorepository.org.uk/ |

- 1. **Software**

| **Software name** | **Manufacturer** | **Version** |
| --- | --- | --- |
| Fiji / Image J | National Institutes of Health | 2.1.051 |
| Image Lab | Biorad | 6.1 |
| ZenBlue Software | Zeiss | 3.1 |
| Wave Seahorse analysis | Agilent | 2.6.3 |
| ViiA7 Real-time PCR detection system | Applied Biosystems | V1.6.1 |
| OMERO web-client | 2005-2023 University of Dundee & Open Microscopy Environment | 5.19.0 |
| Waters TargetLynx software | WATERS | 4.2 |
| R statistical language | The R Foundation | 3.00 |
| GraphPad Prism | Dotmatics | 10.0.3 |

- 1. **Other (e.g. drugs, proteins, vectors etc.)**

| 3,5-diethoxycarbonyl-1,4-dihydrocollidine (DDC) | Ssniff Diets | S9883-P720 |
| --- | --- | --- |
| Ganciclovir (GCV) | Sigma | PHR1593 |
| Fexaramine | Cymitquimica | AN-AG0038ME |
| Ampicillin | Sigma | A0166 |
| Vancomycin | Sigma | 94747 |
| Neomycin | Sigma | N6386 |
| Metronidazole | Sigma | M1547 |
| ABT-263 | Stratech | A3007-APE |
| Aspartate aminotransferase (AST) | Randox | AS8306 |
| Alanine aminotransferase (ALT) | Randox | AL8304 |
| Alkaline Phosphatase (AP) | Randox | AP8303 |
| 10% Neutral buffered formalin | Sigma | HT501320-9.5L |
| Antibody diluent | Dako | S0809 |
| terminal deoxynucleotidyl transferase dUTP nick end labeling (TUNEL) assay | Roche | 11684809910 |
| DAPI-mounting solution | Vector Laboratories | H-1200 |
| DAB^+^ chromogen system | Dako | K3468 |
| NeoMount mounting solution | Sigma | 109016 |
| D4 internal standard: Deoxycholic Acid-D4 | Steraloids | C1070-015 |
| D4 internal standard: Lithocholic Acid-D4 | Steraloids | C1420-015 |
| D4 internal standard: Cholic Acid-D4 | Steraloids | C1900-015 |
| D4 internal standard: Glycocholic Acid-D4 | Steraloids | C1925-015 |
| D4 internal standard: Chenodeoxycholic Acid-D4 | Steraloids | C0940-015 |
| D4 internal standard: Glycochenodeoxycholic Acid-D4 | Steraloids | C0960-015 |
| Supelco Ascentis Express C18 150 x 4.6, 2.7µm column | Sigma | 53829-U |
| Supelco 2.7 μm particle size, L × I.D. 5 mm × 4.6 mm guard column | Sigma | 53508-U |
| Oasis PRiME HLB 30 milligram µElution 96-well plate | Waters | 186008054 |
| Qiazol | Qiagen | 79306 |
| M-MLV Reverse Transcriptase | Invitrogen | 28025013 |
| SYBR Green reagent | Life Technologies | 10187094 |
| RPMI 1640 media | Sigma | R8758 |
| Glutamine | Sigma | G7513 |
| Pen/Strep | Lonza | DE17-602E |
| Lipopolysaccharides (LPS) from *Escherichia coli* O55:B5 | Sigma | L2637 |
| Deoxycholic acid (DCA) | Sigma | D2510 |
| Senescence β-Galactosidase Staining kit | Cell signaling | 9860S |
| FITC-Dextran | Sigma | FD4 |
| LPS-Binding Protein ELISA | Abcam | AB279407 |
| P7 and P5 of Nextera XT Index Kit v2 index primers | Illumina | FC-131-2001 to 2004 |

- 1. **Please provide the details of the corresponding methods author for the manuscript:**

| Naiara Beraza  Quadram Institute Bioscience  Rosalind Franklin Road,  Norwich Research Park,  Norwich NR4 7UQ  T:  +44 (0)1603 251413  Naiara.beraza@quadram.ac.uk |
| --- |

**2.0 Please confirm for randomised controlled trials all versions of the clinical protocol are included in the submission. These will be published online as supplementary information.**

|  |
| --- |
